# Tryptophan derivatives regulate the transcription of Oct4 in stem-like cancer cells

**DOI:** 10.1038/ncomms8209

**Published:** 2015-06-10

**Authors:** Jie Cheng, Wenxin Li, Bo Kang, Yanwen Zhou, Jiasheng Song, Songsong Dan, Ying Yang, Xiaoqian Zhang, Jingchao Li, Shengyong Yin, Hongcui Cao, Hangping Yao, Chenggang Zhu, Wen Yi, Qingwei Zhao, Xiaowei Xu, Min Zheng, Shusen Zheng, Lanjuan Li, Binghui Shen, Ying-Jie Wang

**Affiliations:** 1 State Key Laboratory for Diagnosis and Treatment of Infectious Diseases, Collaborative Innovation Center for Diagnosis and Treatment of Infectious Diseases, The First Affiliated Hospital, School of Medicine, Zhejiang University, Hangzhou, Zhejiang 310003, China; 2 College of Life Sciences, Zhejiang University, Hangzhou, Zhejiang 310058, China; 3 AhR Pharmaceuticals Inc., Madison, Wisconsin, 53719, USA; 4 Department of Hepatobiliary and Pancreatic Surgery, The First Affiliated Hospital, School of Medicine, Zhejiang University, Hangzhou, Zhejiang 310003, China; 5 Department of Pharmacy, The First Affiliated Hospital, School of Medicine, Zhejiang University, Hangzhou, Zhejiang 310003, China; 6 Department of Pathology and Laboratory Medicine, Perelman School of Medicine, University of Pennsylvania, Philadelphia, Pennsylvania 19104, USA; 7 Department of Radiation Biology, City of Hope National Medical Center and Beckman Research Institute, Duarte, California 91010, USA

## Abstract

The aryl hydrocarbon receptor (AhR), a ligand-activated transcription factor that responds to environmental toxicants, is increasingly recognized as a key player in embryogenesis and tumorigenesis. Here we show that a variety of tryptophan derivatives that act as endogenous AhR ligands can affect the transcription level of the master pluripotency factor Oct4. Among them, ITE enhances the binding of the AhR to the promoter of Oct4 and suppresses its transcription. Reduction of endogenous ITE levels in cancer cells by tryptophan deprivation or hypoxia leads to Oct4 elevation, which can be reverted by administration with synthetic ITE. Consequently, synthetic ITE induces the differentiation of stem-like cancer cells and reduces their tumorigenic potential in both subcutaneous and orthotopic xenograft tumour models. Thus, our results reveal a role of tryptophan derivatives and the AhR signalling pathway in regulating cancer cell stemness and open a new therapeutic avenue to target stem-like cancer cells.

## Abstract (web summary)

The aryl hydrocarbon receptor, AhR, can regulate Oct4, which is often expressed in cancer stem cells and promotes pluripotency and tumorigenesis. Here, in cancer stem cells, AhR is shown to be activated by the tryptophan derivative ITE, which causes transcriptional repression of Oct4 and reduced tumorigenesis.

## Graphical abstract

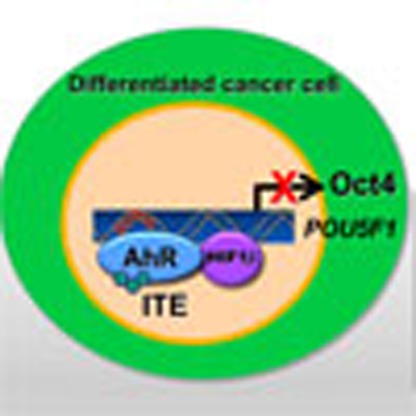

The aryl hydrocarbon receptor (AhR), a ligand-activated transcription factor originally identified and characterized as a key factor responding to environmental toxicants, is now gaining increasing attention for its critical roles in immune responses^1^,^2^ and carcinogenesis^3^,^4^. It has either oncogenic or tumour-suppressive activities, depending on each specific ligand that can distinctly bind to its promiscuous ligand-binding pocket^3^,^4^,^5^. The best characterized high-affinity ligands for the AhR are synthetic halogenated aromatic hydrocarbons and polycyclic aromatic hydrocarbons^3^,^4^,^5^, and a variety of its natural ligands with remarkably different structures and physicochemical characteristics have also been identified and characterized^3^,^4^,^5^,^6^,^7^.

More recently, potential roles of the AhR and its synthetic ligands in stem cell and cancer stem cell biology start to be appreciated. For instance, tranilast, a small-molecule drug for treating allergic and fibrotic diseases, and the synthetic agonist of the AhR, can downregulate the master pluripotency factor Oct4 in stem-like breast cancer cell lines and inhibit their proliferation and metastasis by an unidentified mechanism^8^. Yen and co-workers^9^ reported that retinoic acid (RA)-induced differentiation of leukaemia cells correlated with increased AhR levels and decreased Oct4 levels, implicating a negative correlation between these two factors in cancer stem cells; however, the underlying mechanism remained unexplored. Moreover, AhR's synthetic antagonist StemRegenin 1 (refs 10, 11) can induce the self-renewal and expansion of haematopoietic stem cells and leukaemic stem cells. However, thus far, it remains unknown whether any natural or endogenously produced AhR ligands can control the expression of Oct4 in normal stem cells or stem-like cancer cells, and what the underlying mechanisms might be. Among AhR's natural ligands, tryptophan derivatives such as 6-formylindolo[3,2-b]carbazole (FICZ)^12^, Kynurenine (Kyn)^6^ and 2-(1′H-indole-3′-carbonyl)-thiazole-4-carboxylic acid methyl ester (ITE)^13^ have received increasing attention for their emerging roles in cancer immunology. Overly consumption or deprivation of tryptophan represents the key features of tumour microenvironment, and consequent accumulation of the low-affinity AhR agonist Kyn is associated with tumour progression^14^. In contrast, ITE, the endogenous high-affinity AhR agonist^13^,^15^, possesses potent anticancer activity but the mechanism of action remains unclear^16^. Here we reveal a transcriptional link between the tryptophan metabolites (particularly ITE) and Oct4 that is mediated by the AhR. Endogenous ITE can stimulate the binding of AhR to the promoter of Oct4 and suppress its transcription. Reduction of endogenous ITE levels in cancer cells by tryptophan deprivation or hypoxia led to Oct4 elevation, which can be reverted by administration with synthetic ITE. Consequently, synthetic ITE induced the differentiation of stem-like cancer cells and reduced their tumorigenic potential in mouse xenograft tumour models.

## Results

### AhR binds directly to the Oct4 promoter

**Figure 1:**
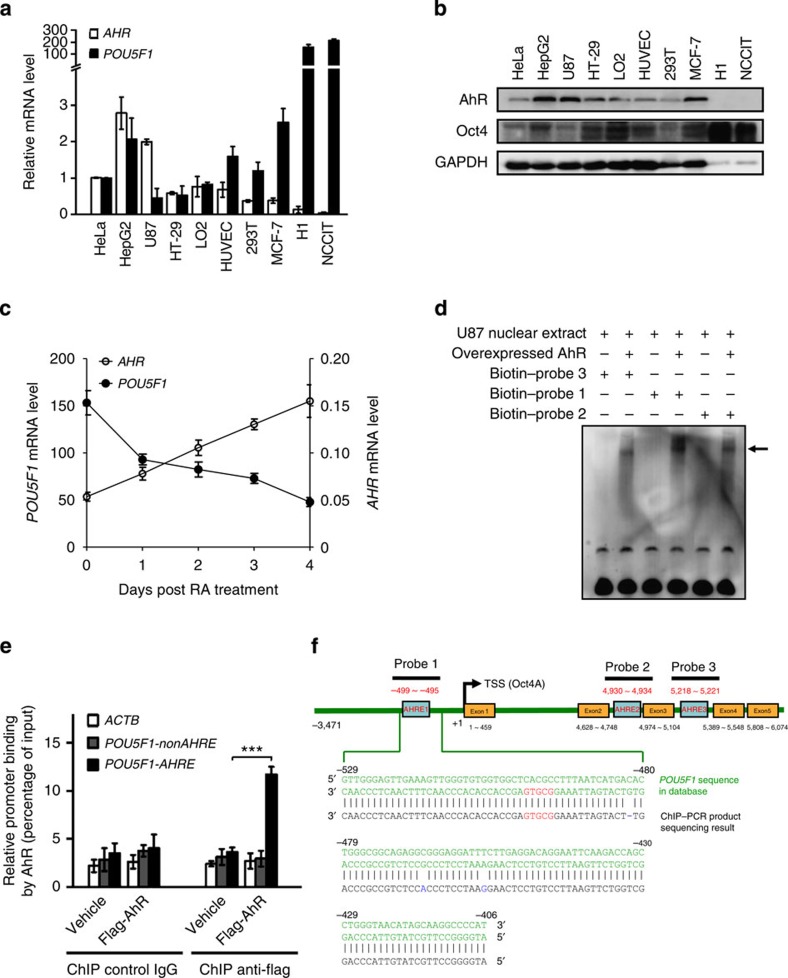
AhR directly binds to the *POU5F1* promoter. (**a**) *AHR* and *POU5F1* mRNA levels in 10 human cell lines determined using qRT–PCR with their levels in HeLa cells being set as 1. (**b**) AhR and Oct4 protein levels in the above cell lines determined by immunoblotting, for H1 and NCCIT cells, the amount of total protein loaded was only 1/40 of the other cells. (**c**) Increased *AHR* and decreased *POU5F1* mRNA levels during 10 μM RA-induced differentiation of NCCIT cells over 4 days, determined using qRT–PCR. Values were normalized to those of HeLa cells. (**d**) EMSA of three sets of biotinylated *POU5F1* probes (relative positions shown in **f**) incubated with nuclear extracts of U87 cells or U87 cells ectopically expressing *Flag-AhR*. The arrow indicates AhR-bound probe. (**e**) U87 cells were infected with lentiviruses harbouring *Flag-AHR* for 3 days, and ChIP-based analysis was carried out with anti-Flag M2 beads. The data in **a**,**c**,**e** were expressed as mean±s.d. of triplicate measurements from one of three independent experiments that gave similar results. The statistical significance of compared measurements was evaluated using the two-tailed unpaired Student's *t*-test. ***P<0.001. (**f**) Alignment of partial *POU5F1* promoter sequence in database (green) and that obtained by sequencing the ChIP–PCR product (black) in **e**, using the PCR primers detailed in Supplementary Fig. 9. The mismatched or missing nucleotides are marked in blue, and the putative AHREs are marked in red.

To explore the potential correlation between AhR and Oct4 (encoded by the *POU5F1* gene) expression, we compared their mRNA levels in two human pluripotent stem cell lines (embryonic stem cell (ESC) H1, embryonal carcinoma cell (ECC) NCCIT), five human cancer cell lines (HeLa, HepG2, U87, HT-29 and MCF-7) and three human non-tumour cell lines (HUVEC, LO2 and 293T; Fig. 1a). The two pluripotent stem cells showed the highest Oct4 (*POU5F1*) mRNA levels, yet the lowest *AHR* mRNA levels; however, in general there was no significant correlation between *POU5F1* and *AHR* mRNA levels in the cell lines examined, nor were there correlations between the mRNA levels of AhR and its two hallmark target genes *CYP1A1* and *CYP1B1* (Supplementary Fig. 1). When an expanded panel of human cancer cells was analysed, the correlation between the *POU5F1* and *AHR* mRNA levels was still not obvious (Supplementary Fig. 2). The AhR protein levels in most examined cell lines correlated well with their mRNA levels (Fig. 1b versus Fig. 1a), while the Oct4 protein levels in all non-stem cell lines were much lower than those of the two pluripotent stem cells and did not correlate with the corresponding mRNA levels (Fig. 1b versus Fig. 1a). Nevertheless, the Oct4 proteins in non-stem cell lines can be specifically reduced by an short hairpin RNA (shRNA) targeting the 3′-untranslated region of the *POU5F1* (Supplementary Fig. 3). Among various normal human tissues, although placenta derived from the Oct4-deficient trophectoderm exhibited the highest level of *AHR* mRNA, in general there was a positive correlation between the *POU5F1* and *AHR* mRNA levels (Supplementary Fig. 4). To explore whether AhR expression is associated with Oct4 expression during stem cell differentiation, we determined the mRNA levels of these two genes in ESCs (Supplementary Fig. 5) and ECCs (Fig. 1c) subject to RA treatment. Consistent with previous studies^17^,^18^, pluripotent stem cells treated with 10 μM RA for 4 days progressively resulted in differentiation (Supplementary Fig. 6). The *POU5F1* mRNA level decreased over time, which was correlated with an increase in the *AHR* mRNA level (Fig. 1c, Supplementary Fig. 7), suggesting that there is a strong negative correlation between the AhR and Oct4 expression during stem cell differentiation.

To determine whether AhR could bind to the *POU5F1* gene and regulate its transcription, we first conducted an *in silico* search for canonical AhR-responsive elements (AHREs) at the regulatory and coding regions of the *POU5F1* gene. Although no potential AHRE can be picked up in the *POU5F1* pseudogenes, three putative AHREs were identified in the *POU5F1* gene (Supplementary Fig. 8). Since U87 cells contain relatively high levels of AhR (Fig. 1a,b), we synthesized biotin-labelled DNA probes spanning the above three AHREs and tested their binding with AhR in U87 nuclear extracts using electrophoretic mobility shift assay (EMSA). While all the three probes can bind to AhRs in EMSA (Fig. 1d), we focused on the probe spanning the AHRE numbered −499∼−495 relative to the transcription start site because it bound with the highest affinity and corresponded to the promoter region of the *POU5F1*. Next, we examined the AhR–*POU5F1* promoter interaction in U87 cells using chromatin immunoprecipitation (ChIP) assays. When ectopically expressed, Flag-AhR was specifically pulled down by anti-Flag M2 beads together with the *POU5F1–AHRE* DNA fragments (Fig. 1e). Sequencing of the DNA fragments amplified with specific AHRE-targeting primers (Supplementary Fig. 9) confirmed that they truly contained the presumed AHRE sequence (Fig. 1f). In comparison, neither the DNA fragments of β-actin gene (*ACTB*) nor those of a nonAHRE region in the *POU5F1* (+752∼+849, *POU5F1-nonAHRE*, Supplementary Fig. 9) could be pulled down substantially by anti-Flag (Fig. 1e), indicating that the binding of AhR to the AHRE (−499∼−495) in the *POU5F1* promoter is specific.

### ITE-activated AhR inhibits Oct4 transcription

**Figure 2:**
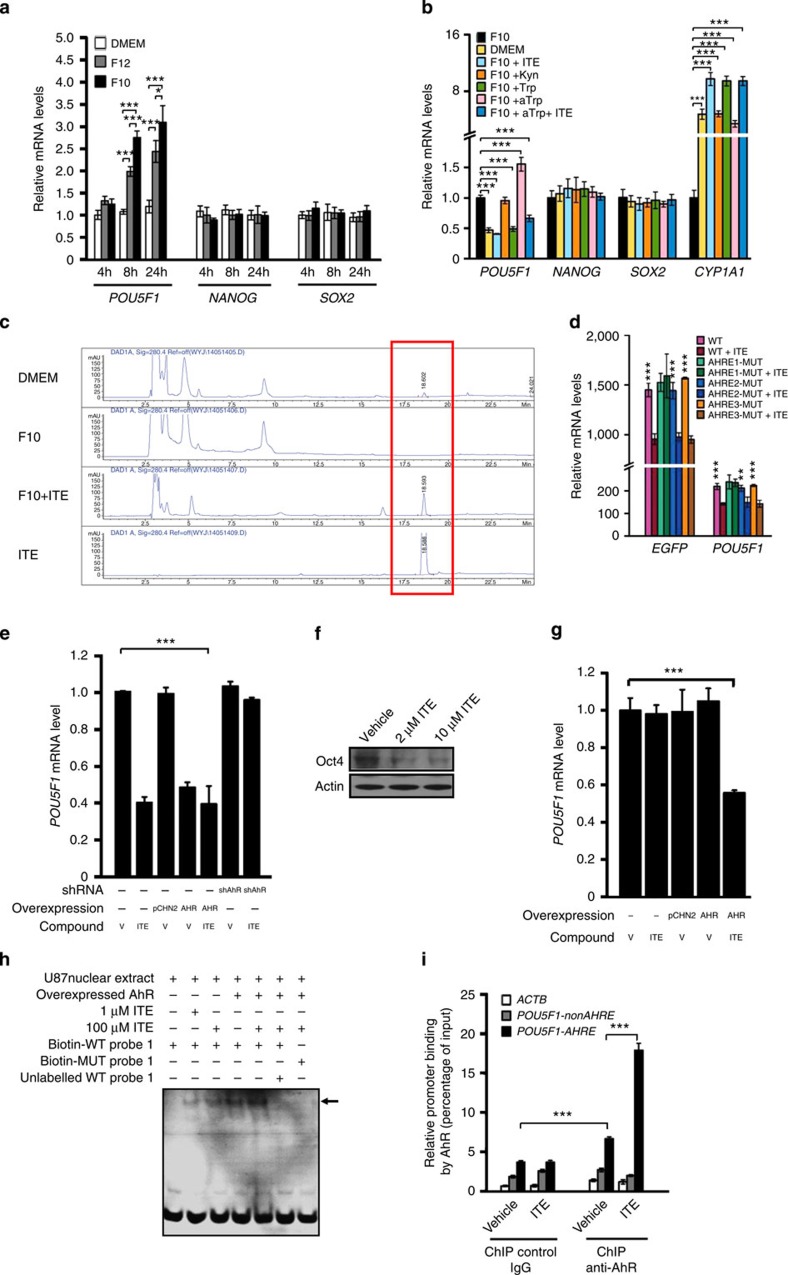
Tryptophan derivative ITE promotes the binding of AhR to the *POU5F1* promoter and suppresses Oct4 transcription. (**a**) U87 cells grown in tryptophan-rich medium (DMEM) or low-tryptophan medium (F12 and F10) for varying time were harvested and analysed using qRT–PCR for mRNA levels of the indicated genes. (**b**) U87 cells were cultured in DMEM, F10 alone or F10 supplemented with either ITE (10 μM), Kyn (50 μM), Trp (1 μM), aTrp or aTrp plus 10 μM ITE for 8 h. Cells were harvested and analysed using qRT–PCR for mRNA levels of the indicated genes. (**c**) Identification of endogenous ITE in U87 cell cultures. Reverse phase HPLC elution profile of major metabolites of U87 cells cultured in the DMEM (DMEM) or F10 medium supplemented with vehicle (F10) or 10 μM ITE (F10+ITE) for 8 h. Purified synthetic ITE (ITE) was added as a reference sample. The peak at 18.6 min of retention time presumably corresponds to ITE (marked by red box). (**d**) U87 cells were transfected with phOCT4-EGFP reporter-based plasmids harbouring normal AHREs (WT) or one of the three AHRE mutants, and treated with or without 10 μM ITE for 24 h. Cells were harvested and analysed using qRT–PCR for mRNA levels of the indicated genes. (**e**) U87 cells were infected with lentiviruses harbouring an empty vector pCHN2 (pCHN2), the *Flag-AHR* (AHR) or an shRNA against *AHR* (shAhR) for 3 days, treated with vehicle (V) or 10 μM ITE (ITE) for 8 h, and analysed using qRT–PCR for the mRNA levels of the *POU5F1*. (**f**) Immunoblots showing Oct4 protein levels in U87 cells treated with varying concentrations of ITE for 5 days. (**g**) NCCIT cells were infected with lentiviruses harbouring an empty vector pCHN2 (pCHN2) or the *Flag-AHR* (AHR) for 3 days, treated with vehicle (V) or 10 μM ITE (ITE) for 2 h, and analysed using qRT–PCR for the mRNA levels of the *POU5F1*. (**h**) EMSA of biotinylated WT or mutant (MUT) *POU5F1* probe 1 incubated with nuclear extracts of U87 cells or U87 cells ectopically expressing Flag-AhR, supplemented with or without ITE. The arrow indicates AhR-bound probe. (**i**) U87 cells were treated with 10 μM ITE for 2 h, and ChIP-based analysis was carried out with anti-AhR. The data in **a**,**b**,**d**,**e**,**g**,**i** were expressed as mean±s.d. of triplicate measurements from one of three independent experiments, which gave similar results. The statistical significance of compared measurements was evaluated using the one-way analysis of variance (ANOVA). *P<0.05, **P<0.01, ***P<0.001.

The binding of AhR to the *POU5F1* promoter *in vitro* (Fig. 1d) and *in vivo* (Fig. 1e,f) in the absence of any exogenously added ligands prompted us to search for its endogenous ligand(s) that controls *POU5F1*-binding. Since tryptophan derivatives (Supplementary Fig. 10) emerged to be important endogenous ligands of the AhR^6^,^12^,^13^, we compared the mRNA levels of the *CYP1A1*, a hallmark target gene of AhR, in U87 cells cultured in media with varying concentrations of L-tryptophan that may differentially activate or inhibit the AhR via its endogenous derivatives. Remarkably, culture in DMEM medium that contains a higher content of L-tryptophan (16 mg l^−1^ compared with 2.04 and 0.6 mg l^−1^ in F12 and F10 media, respectively) yielded a much higher *CYP1A1* transcription (Supplementary Fig. 11) while a much lower *POU5F1* transcription (Fig. 2a). Adding synthetic L-tryptophan (Trp), ITE (Fig. 2b) or FICZ (Supplementary Fig. 12), but not L-Kyn or light-activated tryptophan (aTrp) (Fig. 2b), into the F10 medium brought the *POU5F1* level back to that in DMEM, indicating that some of them could be the sought-for endogenous AhR ligands that suppress *POU5F1* transcription in U87 cells. Although it is difficult to see a dramatic translocation of AhRs from the cytoplasm to the nucleus on ITE addition (Supplementary Figs 13 and 14), the significant upregulation of the *CYP1A1* transcription (Fig. 2b, Supplementary Fig. 15) strongly confirmed the effectiveness of this compound. Importantly, the presence of endogenous ITE in U87 cells cultured in DMEM but not in F10 was detected using HPLC analysis (Fig. 2c, Supplementary Fig. 16). Our peak quantitation (Supplementary Fig. 16) showed that the apparent endogenous ITE peak area in the DMEM group and the synthetic ITE peak area in the F10+ITE group were ∼1/100 and 1/20 of that of the synthetic ITE standard, which corresponded to actual values of 1/20 and 1/4, respectively, given the ∼20% recovery rate of the HPLC sample preparation. As the synthetic ITE standard concentration was 10 μM, the actual endogenous ITE concentration in U87 cells grown in the DMEM medium was thus estimated to be ∼0.5 μM. Since the estimated Ki of ITE for AhR was 3 nM (ref. 13), and the estimated IC_50_ of ITE for OVCAR-3 cell proliferation was 0.2 nM (ref. 16), such a level of endogenous ITE is presumably high enough in exerting its biological functions. Remarkably, synthetic ITE decayed rapidly in F10 cell culture and only 1/4 of the initially added 10 μM ITE remained after 8 h (Supplementary Fig. 16). A dose response study with U87 cells cultured in the F10 medium showed that an initially added 0.5 μM synthetic ITE (which was supposed to decay over time) was able to reduce the Oct4 mRNA level to the same extent as the DMEM medium did (Supplementary Fig. 17), indicating that the endogenous ITE produced by U87 cells in DMEM (at ∼0.5 μM) is likely to be sufficient to maintain the Oct4 mRNA at a low ‘basal' level (as opposed to the ‘elevated' Oct4 mRNA level in F10 culture). Increasing the concentrations of synthetic ITE added into the F10 medium further lowered the Oct4 mRNA levels by ∼30%, and such ‘additional' Oct4-suppressing effect reached maximum at 10 μM ITE, regardless of being replenished at an 8-h interval (Supplementary Fig. 17) or a 2-h interval (Supplementary Fig. 18). When cells were grown in DMEM, the ‘additional' Oct4-suppressing effect of synthetic ITE became even more prominent. However, such an effect can be reverted if no ITE was replenished after 8 h (Supplementary Fig. 19) possibly due to its fast metabolism by cellular enzymes^13^,^15^, and, therefore, in the following compound treatment studies that lasted for longer duration synthetic ITE was routinely added in excess (at 10 μM) into the culture medium every 8 h.

When U87 cells were transfected with the phOCT4-EGFP reporter (Supplementary Fig. 20) and treated with synthetic ITE, the levels of both endogenous *POU5F1* and overexpressed *EGFP* fused with *POU5F1* (designated as *EGFP*, Supplementary Fig. 21) decreased by ∼40%, implicating a common inhibitory mechanism. To explore the mechanism, U87 cells were transfected with phOCT4-EGFP reporter-based plasmids harbouring normal AHREs (wild type, WT) or one of the three AHRE mutants (Supplementary Fig. 22), and treated with 10 μM ITE for 24 h. Strikingly, the AHRE1 mutant, but not the other two AHRE mutants, almost completely abolished ITE-induced suppression of *POU5F1* transcription (Fig. 2d), suggesting that ITE regulates the *POU5F1* transcription via AhRs that specifically bound to the AHRE1 motif. To directly examine whether the AhR is necessary for the suppression of *POU5F1* by ITE, the endogenous AhR level in U87 cells was reduced by 50% with a specific shRNA (Supplementary Fig. 23). Under such a condition, synthetic ITE lost its suppressive effect on *POU5F1* transcription (Fig. 2e), validating that ITE did function via the AhR. When Flag-AhR was overexpressed (Supplementary Fig. 23), the *POU5F1* mRNA level was significantly reduced comparing to the pCHN2 vector-expressing cells; however, the supplemented ITE only caused marginal additional reduction (Fig. 2e), indicating that most overexpressed AhRs may have been occupied by elevated endogenous ITE. As expected, when treated with ITE for 3 days, the protein level of Oct4 in U87 cells decreased significantly (Fig. 2f). To consolidate the above finding in a different cellular context, we analysed the transcription of *POU5F1* in AhR-overexpressing NCCIT cells treated with or without ITE. In consistent with the above data (Fig. 1a,b), the endogenous AhR level in NCCIT was very low (Supplementary Fig. 24), and thus ITE treatment did not change the *POU5F1* mRNA level (Fig. 2g). Unlike in U87 cells (Fig. 2e), however, ectopically overexpressing AhR in NCCIT cells *per se* did not lower the *POU5F1* transcription but its suppressive effect was manifested when ITE was added (Fig. 2g), confirming that the *POU5F1*-suppressing effect of AhR is controlled by ITE. EMSA (Fig. 2h) and ChIP (Fig. 2i, Supplementary Fig. 25) experiments demonstrated that both endogenous and overexpressed AhRs bound to the *POU5F1* promoter in an ITE-dependent manner. Collectively, these results indicated that the AhRs that are constitutively activated by endogenous ITE produced in differentiated cancer cells, specifically bind to the AHRE1 of the *POU5F1* promoter *in vivo*, thereby suppressing the transcription of Oct4.

### Synthetic ITE inhibits hypoxia-induced Oct4 elevation

**Figure 3:**
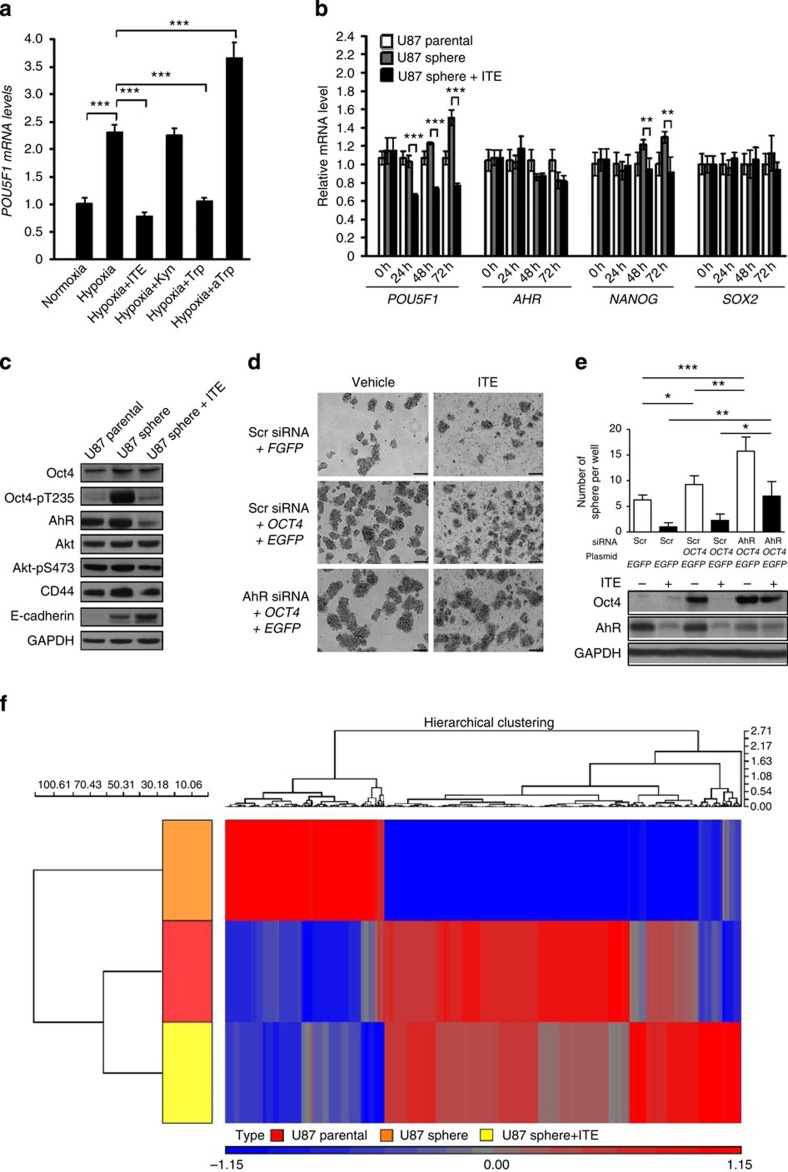
ITE inhibits hypoxia- and tumour sphere-induced Oct4 elevation and leads to the differentiation of stem-like cancer cells. (**a**) U87 cells were seeded at a density of 2 × 10^5^ per well of the six-well plate, cultured under normoxia (21% O_2_) in DMEM for 24 h before being switched to the F10 medium and treated for 8 h with either 21% O_2_ (normoxia) or 1% O_2_ (hypoxia) in the presence of ITE (10 μM), Kyn (50 μM), Trp (1 μM) or aTrp. Cells were harvested and analysed using qRT–PCR for the mRNA levels of the *POU5F1*. The data were expressed as mean±s.d. of triplicate measurements from one of three independent experiments. (**b,c**) Adherent parental U87 cells (U87 parental), U87 tumour sphere cells treated with vehicle (U87 sphere) or 10 μM ITE (U87 sphere+ITE) for varying duration were harvested and analysed using qRT–PCR for the mRNA levels of the indicated genes (**b**) and by immunoblotting (samples treated for 72 h) for the indicated proteins (**c**). (**d**) U87 cells grown in DMEM were transfected with a Scr siRNA or an AhR siRNA in combination with a plasmid containing *EGFP* only or *EGFP*+*OCT4*. The transfected cells were simultaneously treated with DMSO (vehicle or −) or 10 μM ITE. Twenty-four hours after transfection, cells were cultured in a neural stem cell medium to induce the formation of tumour spheres. The pictures were taken 3 days after transfection. Scale bars, 100 μm. (**e**) The cultures described in **d** were quantified for the numbers of tumour spheres formed and analysed for the indicated proteins by immunoblotting. The data were expressed as mean±s.d. of three independent experiments. (**f**) Heat map generated from DNA microarray data for adherent parental U87 cells (U87 parental), U87 tumour sphere cells treated with vehicle (U87 sphere) or 10 μM ITE (U87 sphere+ITE) for 7 days. In **a**,**b**,**e**, The statistical significance of compared measurements was evaluated using the one-way ANOVA. *P<0.05, **P<0.01, ***P<0.001.

As hypoxia represents another key feature of the tumour microenvironment that can induce the expression of a variety of ESC signature genes including *POU5F1* (ref. 19), we wondered whether that may be due to reduced ITE to de-repress the Oct4 transcription. To this end, we determined the endogenous ITE level in U87 cells cultured under 1% O_2_ hypoxic condition. Unlike the normoxia condition where endogenous ITE was detectable when cells were grown in tryptophan-rich DMEM (Fig. 2c, Supplementary Fig. 16), the ITE peak was completely missing in hypoxia-exposed cells grown in DMEM and profoundly shifting in the F10 medium supplemented with synthetic ITE (Supplementary Fig. 26), indicating a faster metabolism of ITE under hypoxic conditions. In parallel, the *POU5F1* transcription level in hypoxia-treated cells was elevated by 2.3-fold compared with normoxia-treated cells, which can be blocked by supplemented ITE and tryptophan (Fig. 3a).

### Synthetic ITE induces stem-like cancer cell differentiation

Given considerable evidence for the critical roles of Oct4 in stem-like cancer cells^20^, we went on to determine the effect of ITE on Oct4 expression in glioblastoma tumour spheres (or neurospheres) that are rich in glioblastoma stem-like cells. During the formation and expansion of the U87 tumour spheres (Supplementary Fig. 27), there was a gradual increase in the *POU5F1* transcript levels starting from 24 h (Supplementary Fig. 28, Fig. 3b), a point at which no endogenous ITE from the tumour spheres can be detected (Supplementary Fig. 29). It was accompanied by a slight decrease in the *AHR* transcript levels (Fig. 3b). ITE (10 μM), when replenished at an 8-h interval (Supplementary Fig. 29), blocked the increase in the *POU5F1* transcripts in U87 tumour spheres while had minimal effect on other pluripotency factors in 3 days (Fig. 3b). Under such conditions, however, there were similar levels of total Oct4 protein among adherent U87 parental cells, U87 tumour spheres and ITE-treated U87 tumour spheres (Fig. 3c), and only prolonged treatment with ITE could reduce the Oct4 protein levels in tumour spheres (Supplementary Fig. 30), indicating that the Oct4 proteins in stem-like cancer cells are subjected to multiple layers of regulation and are probably more stable than Oct4 in pluripotent stem cells. We have recently shown that human Oct4 can be phosphorylated directly by Akt at threonine 235 (Oct4-pT235), which promotes the self-renewal and survival of ECCs^17^. Here we found that the level of Oct4-pT235 was rapidly and dramatically increased during the formation of U87 tumour spheres and such an increase was largely blocked by the ITE treatment (Fig. 3c). Remarkably, ITE reduced the total AhR protein level (Fig. 3c) but not its mRNA level (Fig. 3b), indicating its direct action at the post-translational level or indirect regulation via other targets. Since AhR plays differential or even opposing roles in tumorigenesis depending on its binding ligands^3^,^4^,^5^, the reduction of oncogenic factors (such as Akt-pS473 and Oct4-pT235) and the increase in tumour-suppressive factors (such as E-cadherin; Fig. 3c) indicated that ITE may specifically reduce the oncogenic pool of AhR while maintaining and activating the tumour-suppressive pool of AhR that transcriptionally repressed Oct4.

Consequently, ITE inhibited the formation of U87 tumour spheres (Fig. 3d,e, Supplementary Fig. 31) and the proliferation of sphere cells (Supplementary Fig. 31), resembling the phenotypes of glioblastoma cells treated with Oct4 short interfering RNA (siRNA)^21^. To determine whether in the context of tumour spheres the Oct4-suppressing effect of ITE is also mediated through AhR, U87 cells grown in DMEM were transfected with a scrambled (Scr) siRNA or an AhR siRNA in combination with a plasmid containing *EGFP* only or *EGFP*+*OCT4*. The transfected cells were simultaneously treated with vehicle or 10 μM ITE. Twenty-four hours later, cells were cultured in a neural stem cell (NSC) medium to induce the formation of tumour spheres. Seventy-two hours after transfection and compound treatment, the Oct4 overexpression group had a significantly increased number of tumour spheres (Fig. 3e), confirming that Oct4 can promote the formation of tumour spheres. Adding ITE to this group brought the Oct4 mRNA level down to the same degree as that seen with the nontransfected group, suggesting that supplemented ITE was able to suppress the overexpressed Oct4. Interestingly, the group with overexpressed Oct4 and its endogenous AhR knocked down simultaneously had an even greater number of tumour spheres that were accompanied by a significantly higher level of overexpressed Oct4 protein (Fig. 3e), implicating a negative regulation of Oct4 expression by AhR in the context of tumour spheres. Under such AhR knockdown conditions, the Oct4-suppressing effect of ITE was significantly diminished (Fig. 3e), suggesting that ITE did function through the AhR. Further genome-wide microarray analysis revealed that several stemness-related signalling pathways (such as the transforming growth factor-β, Notch, Wnt and fibroblast growth factor (FGF) signalling pathways) appeared to be activated in U87 tumour spheres, which were downregulated by synthetic ITE (Supplementary Fig. 32 and Supplementary Data set 1). In contrast, there was no global upregulation of the AhR target genes in tumour spheres comparing with their parental cells (Supplementary Fig. 33). ITE-treated tumour sphere cells had transcriptional profiles that were much more similar to those of parental cells than untreated sphere cells (Fig. 3f, Supplementary Data set 1), indicating that ITE induced the differentiation of stem-like cancer cells towards parental cancer cells.

### ITE reduces the tumorigenicity of stem-like cancer cells

**Figure 4:**
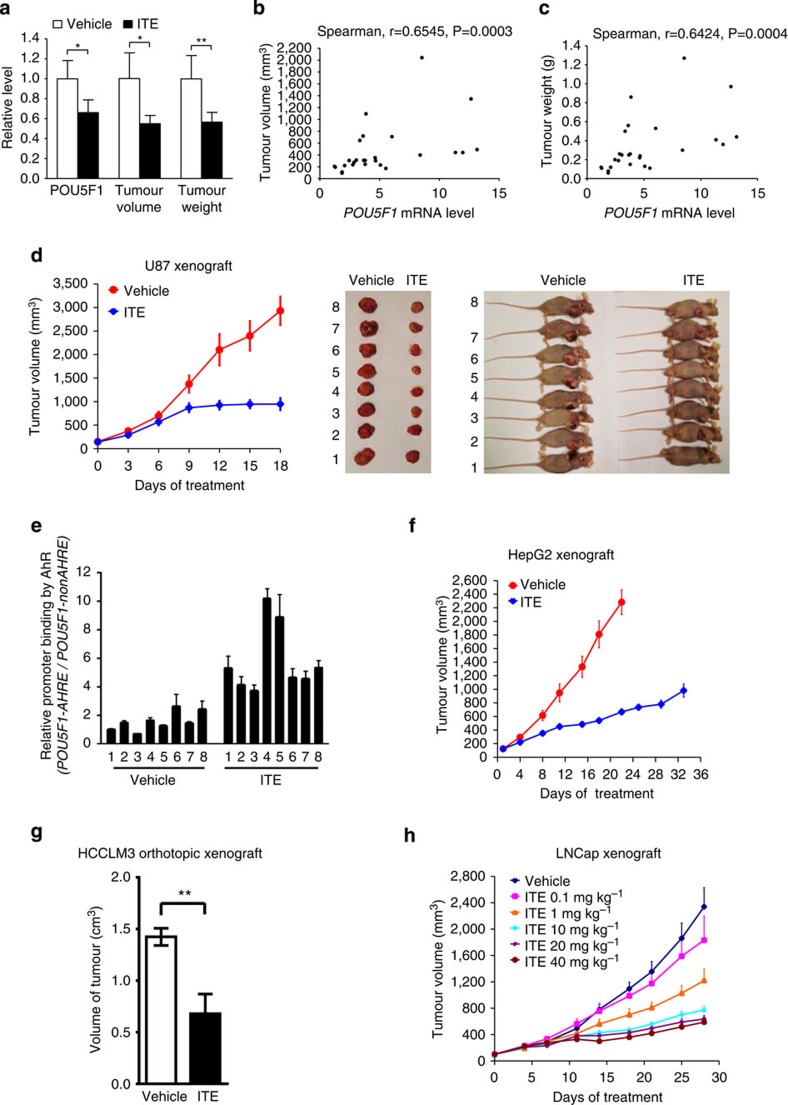
ITE effectively suppresses tumour growth in mice. (**a–c**) U87 cells were inoculated subcutaneously into 26 nude mice. After tumour formation, the mice were divided into 13 pairs based on the apparent tumour volume, and each pair of mice was administered with vehicle (DMSO) or ITE intratumorally for three consecutive days. The xenograft tumours were excised and measured for tumour volumes and weights, and samples were assayed for *POU5F1* mRNA levels (**a**). The data were expressed as mean±s.e.m. The statistical significance of compared measurements was evaluated using the two-tailed paired Student's *t*-test. *P<0.05, **P<0.01. The correlations between the *POU5F1* mRNA level and tumour volume (**b**) *POU5F1* mRNA level and tumour weight (**c**) were analysed with the Spearman's correlation analysis method using the SPSS 19.0 statistical software package. (**d**) U87 cells were inoculated subcutaneously into 16 nude mice. After tumour formation, the mice were randomly grouped and intraperitoneally injected with DMSO (vehicle) or ITE at a dose of 80 mg kg^−1^ per day for 18 consecutive days. The averaged tumour volumes of eight mice in each group were calculated every 3 days and plotted (left). The killed mice bearing the tumours (right) and the excised tumours (middle) were also shown. (**e**) The tumour tissues of the eight mice in each group as described in **d** were harvested, and the DNA fragments were immunoprecipitated with anti-AhR, amplified with primers described in Supplementary Fig. 9 and quantified using qPCR. The ratios of *POU5F1–AHRE*/*POU5F1-nonAHRE* were taken as a measure for specific binding of the *POU5F1* promoter by the AhR, which were expressed as mean±s.d. of triplicate measurements from one of three independent experiments. (**f**) HepG2 cells were inoculated subcutaneously into nude mice. The mice were intraperitoneally injected with DMSO (vehicle) or ITE at a dose of 80 mg kg^−1^ per day for 22–33 consecutive days. The averaged tumour volumes of eight mice in each group were calculated every 3–4 days and plotted. (**g**) HCCLM3-RFP cells were orthotopically transplanted into the livers of nude mice. After 20 days, the mice were intraperitoneally injected with DMSO (vehicle) or ITE at a dose of 80 mg kg^−1^ per day for 21 consecutive days, and were then killed. The excised tumour tissues were shown in Supplementary Fig. 39, and the averaged tumour volumes of four mice in each group were calculated and plotted. The statistical significance of compared measurements was evaluated using the two-tailed unpaired Student's *t*-test. **P<0.01. (**h**) LNCap cells were inoculated subcutaneously into nude mice. The mice were intraperitoneally injected with DMSO (vehicle) or ITE at different doses (ranging from 0.1 to 40 mg kg^−1^ per half day) for 28 consecutive days. The averaged tumour volumes of eight mice in each group were calculated every 3–4 days and plotted.

To examine the effect of ITE on Oct4 expression in an *in vivo* setting, U87 cells were inoculated subcutaneously into nude mice. When the xenografted tumours reached relatively small volumes (∼100 mm^3^), ITE was administered intratumorally for three consecutive days, immediately followed by tumour excision (Supplementary Fig. 34) and analyses. ITE significantly reduced the *POU5F1* mRNA levels in tumours, the tumour volumes as well as the tumour weights (Fig. 4a), and there was a positive correlation between the *POU5F1* mRNA level/tumour volume (Fig. 4b) and the *POU5F1* mRNA level/tumour weight when all the mouse samples were analysed together (Fig. 4c). When the mice were divided into the vehicle and ITE groups for separate analyses, both correlations were higher in the vehicle group than in the ITE group (Supplementary Fig. 35), indicating that besides lowering the Oct4 expression, ITE may suppress tumour growth via additional mechanisms. In contrast to the *POU5F1*, the correlations between the *AHR* mRNA level and the tumour volume/tumour weight were not significant in the vehicle group but remarkably increased in the ITE group (Supplementary Fig. 36). When dosed at 80 mg kg^−1^ per day for 18 consecutive days, ITE effectively inhibited the growth of U87 xenografts (Fig. 4d). ChIP assay showed that the binding of AhR to the *POU5F1* promoter in U87 xenografts of vehicle-treated mice was lower than that in parental U87 cells (Supplementary Fig. 37 versus Fig. 2i). ITE administration significantly increased such binding (Fig. 4e, Supplementary Fig. 37), which was accompanied by a general decrease in the *POU5F1* mRNA level (Supplementary Fig. 38). In contrast to the differentiated U87 cells where a relatively high AhR level is accompanied by a low Oct4 level (Fig. 1a,b), there appeared to be a positive correlation between them in U87 xenografts both at the mRNA and protein levels (Supplementary Fig. 38). The dramatic increase in the *POU5F1* and *AHR* mRNA levels in U87 xenografts over parental U87 cells and the suppressive effects of ITE were well reflected at the protein level revealed by immunoblotting (Supplementary Fig. 38). Importantly, with similar AhR protein levels (Supplementary Fig. 38, lower right, Vehicle 4 versus ITE4, Vehicle 5, Vehicle 6 versus ITE 7, Vehicle 7 versus ITE 6, Vehicle 8 versus ITE 5), ITE-treated xenografts mostly had lower Oct4 levels than vehicle-treated xenografts, indicating that it was the ITE treatment but not the AhR level *per se* that affected the tumour growth in such an *in vivo* context.

ITE also significantly inhibited the proliferation of xenografted hepatocellular carcinoma HepG2 cells (Fig. 4f). To better mimic the organ and tissue microenvironments in which the tumours grow, we orthotopically transplanted the reporter-containing HCCLM3-RFP cells derived from another hepatocellular carcinoma line into the livers of nude mice and assessed the anticancer efficacy of ITE. After 21 days of ITE administration, compared with the vehicle-treated mice, there was a significant inhibition in the growth of the transplanted tumours (Fig. 4g, Supplementary Fig. 39 and Supplementary Fig. 40), and this was accompanied by a general reduction in the Oct4 level (Supplementary Fig. 40). Further work with xenografted LNCap cells demonstrated that the anticancer efficacy of ITE was clearly dose-dependent (Fig. 4h).

## Discussion

Our findings reveal a previously unidentified connection between AhR and Oct4 in human pluripotent stem cells and cancer cells. We discovered that AhR can specifically bind to the AHRE motif in the *POU5F1* promoter *in vivo* and act as a transcriptional repressor of *POU5F1*. In human ESCs and ECCs, the extremely low levels of AhR were associated with very high levels of Oct4, consistent with the hypothesis that relieving from the transcriptional suppression by AhR may account for the upregulated Oct4 levels during differentiation. However, recent evidence was provided in mouse ESCs that Oct4/Sox2/Nanog complexes can bind to the AhR distal promoter region to repress AhR expression^22^, raising the possibility of a reciprocal regulatory mechanism. Thus, it is important to investigate in future studies the exact causal relationship between AhR and Oct4 in pluripotent stem cells, and if reciprocal suppression between them may regulate the homeostasis of stem cells during embryogenesis. Since the interplay between AhR and Oct4 could be regulated at multiple levels (for example, transcriptional, post-transcriptional, translational and post-translational) and in different modes (for example, unidirectional or bidirectional, feedback or feed-forward), it is conceivable that in different experimental systems the AhR–Oct4 correlation may be different, and this may explain why the levels of AhR and Oct4 are clearly negatively correlated in pluripotent stem cells, variably correlated in differentiated cells including adherent U87 cells but apparently positively correlated in U87 xenografts. Future studies are required to fully elucidate such differential regulatory mechanisms.

Methylation of the *POU5F1* promoter and enhancer regions has been proposed as the primary mechanism in shutting down Oct4 expression in differentiated cells^23^. Our findings raise the possibility that additional factors (such as certain transcriptional repressors including AhR) may also contribute to such persistent silencing. We found that ITE, an endogenous derivative of L-tryptophan, at its physiologically achievable concentrations, can be engaged with high affinity to the AhR and facilitate its binding to and suppression of the *POU5F1* promoter *in vivo*. Reduction of endogenous ITE levels in cancer cells by tryptophan deprivation or hypoxia led to Oct4 elevation and augmented cancer cell stemness (Supplementary Fig. 41), indicating that the silenced expression of Oct4 in differentiated cancer cells is still (at least partially) activatable and that stemness may be a rather general and flexible quality of tumour cells^24^,^25^. Endogenous tryptophan metabolites may thus serve as priming factors for reversible stemness, and, therefore, their potential roles in regulating the de-differentiation of normal or aberrant somatic cells and the reprogramming of differentiated cells into pluripotency warrant further studies.

Tryptophan is known to metabolize through two major pathways: the generation of serotonin and the formation of Kyn derivatives. The Kyn pathway is mediated primarily by the catalytic activities of indoleamine 2,3-dioxygenase (IDO) found in many immune cells and tryptophan 2,3-dioxygenase (TDO) expressed constitutively in certain types of cancer such as malignant glioma and hepatocellular carcinoma^26^. IDO and TDO in tumours and immune cells can cause depletion of tryptophan from the local microenvironment while resulting in the accumulation of its metabolites such as Kyn. As tryptophan depletion leads to T-cell apoptosis and Kyn suppresses their differentiation via AhR, the overall outcome is immunosuppression. The immunomodulatory role of ITE has also been documented in the literature where it suppresses the Th17 response and induces the formation of the regulatory T cells^27^. Thus, tryptophan metabolism is now recognized as an important factor that controls local antitumour immune responses^26^. However, none of the tryptophan metabolites or endogenous AhR ligands have so far been reported to regulate the Oct4 expression, and our work may offer important clues for potential connections among tryptophan metabolites, tumour microenvironment and cancer stem cell formation. A deeper understanding of how the biosynthesis and metabolism of ITE and other tryptophan metabolites are regulated *in vivo* may shed light on their pathophysiological roles in the whole body as well as at the local tumour microenvironment. Given its efficacy in inducing the differentiation of stem-like cancer cells and reducing their tumorigenic potential in both xenograft and allograft tumour models, ITE and its chemically improved derivatives may hold great promise of combating human cancers.

It remains to be controversial whether different ligands bound to the AhR can lead to different or even opposite biological outcomes, presumably due to varied biochemical or structural properties of the AhRs from different species, different isoforms or differential conformational changes and so on^28^. For ITE, a more confounding factor comes from the observation that it can rapidly and persistently reduce the AhR protein level, most likely by post-translational modifications^16^. Nevertheless, as long as there is a small population (or ‘pool') of AhR that is still activatable by ITE, that ITE-bound AhR pool is able to bind to the Oct4 promoter and suppress its transcription (Fig. 4e, Supplementary Fig. 38). While ITE-bound AhRs bind to many other target genes besides *POU5F1* and its anticancer effects certainly cannot be solely attributed to Oct4 suppression, our results demonstrated that the AhR-mediated Oct4-suppressing effect of ITE contributed significantly to inhibiting the formation and propagation of stem-like cancer cells. In the tumour sphere model, on ITE treatment, the reduction of oncogenic factors (such as Akt-pS473 and Oct4-pT235) and the increase in tumour-suppressive factors (such as E-cadherin; Fig. 3c) indicated that ITE may specifically reduce the oncogenic pool of AhR while maintaining and activating the tumour-suppressive pool of AhR, which transcriptionally repressed Oct4. Thus, the opposing roles of AhR in tumorigenesis documented in the literature may be reconciled by the hypothesis that different natural or artificial AhR ligands may activate or suppress distinct AhR pools governing different target genes, of which some are oncogenic and some are tumour-suppressive^3^,^4^,^5^. Such an assumption is consistent with the observations that a specific AhR antagonist or an AhR siRNA/shRNA that can only inactivate or reduce a portion of the AhR gives rise to different cellular phenotypes from those of Ahr knockout (Ahr^−/−^) mice in which all the AhR proteins are removed^4^. Further studies will be required to test this hypothesis in different experimental settings.

Taken together, our work reveals a novel role of tryptophan derivatives (in particular ITE) and the AhR pathway in regulating cell stemness and opens a new therapeutic avenue to target stem-like cancer cells.

## Methods

### Antibodies and reagents

The monoclonal (sc-5279) and polyclonal (sc-9081) anti-Oct4 antibodies were purchased from Santa Cruz Biotechnology. The polyclonal anti-Oct4-pT235 antibody was custom-made by GenScript (order ID: 134164-1) and verified as previously described^17^. Monoclonal anti-AhR antibody (RPT1) and protein A/G agarose beads (20421) were purchased from Thermo Scientific. Anti-E-cadherin (ab1416) and anti-CK19 (ab52625) were from Abcam. Anti-Akt-pT308 antibody (2965), anti-Akt-pS473 antibody (4060), anti-pan Akt antibody (9614L), anti-CD44 (3570S), peroxidase-conjugated anti-mouse secondary antibody (7076), peroxidase-conjugated anti-rabbit secondary antibody (7074), anti-biotin antibody (7075), mouse IgG conjugated with magnetic beads (5873) and the biotinylated protein ladder detection pack (7727) were all purchased from Cell Signaling Technology. Anti-GAPDH (horseradish peroxidase, HRP; A00192) was from GenScript, and anti-GAPDH (AG019) was from Beyotime Institute of Biotechnology. The dilutions of the above antibodies ranged between 1:1,000 and 1:2,000 when used for immunoblotting. Epidermal growth factor (EGF; E5036), leukaemia inhibitory factor (L5283), anti-Flag M2 magnetic beads (M8823), RA (R2625), dimethylsulphoxide (DMSO; D5879), L-tryptophan (93659) and L-Kyn (K8625) were from Sigma-Aldrich. 6-Formylindolo[3,2-b] carbazole (BML-GR206) was purchased from Enzo Life Sciences, Farmingdale, NY. The Ambion FirstChoice Human Total RNA Survey Panel (AM6000), B-27 supplement minus Vitamin A (12587-010), basic fibroblast growth factor (bFGF; PHG0266), Hoechst 33342 (H3570) and TRIzol (15596-026) were all purchased from Life Technologies. Akti-1/2 (124018) was obtained from Calbiochem. ITE was chemically synthesized by KNC laboratories Co., Ltd. (Tokyo, Japan) and used previously^16^. All chemicals were of AR grade and most were purchased from Sigma. Most laboratory consumables and supplies were from Fisher Scientific. Unless specified, the sources of all the plasmids were described previously^17^.

### Cell culture and treatment

The 293T, U87, HepG2, HeLa, HT-29, HUVEC, MCF-7, NCCIT and LNCap cells were obtained from ATCC, LO2 cells and HCCLM3 cells were from the Cell Bank of the Chinese Academic of Sciences, Shanghai, China. In most cases, cells were cultured and treated in DMEM (Invitrogen 21063-029, containing 16 mg l^−1^ L-tryptophan) supplemented with 10% fetal bovine serum (GIBCO 10099 or Pufei 1101-500), and in some cases cultured in low-tryptophan media Ham's F12 Nutrient Mix (Hyclone 11765, designated as ‘F12') or Ham's F10 Nutrient Mix (Hyclone 31550, designated as ‘F10') that contains 2.04 and 0.6 mg l^−1^ L-tryptophan, respectively. The source and culture conditions for the hESC line H1 (WA01) were as previously described^29^.

For tumour sphere assay^30^, U87 cells were plated at a density of 30,000 cells ml^−1^ in 96-well (for cell proliferation assays) or 6-well plates (for western blot analysis) with NSC medium, which contains DMEM/F12 (Corning R10-092-CV) supplemented with 1% (v/v) penicillin/streptomycin, 2% (v/v) B-27 Supplement Minus Vitamin A, 20 ng ml^−1^ EGF, 20 ng ml^−1^ bFGF and 10 ng ml^−1^ leukaemia inhibitory factor to allow tumour sphere formation. Typical tumour spheres can form after cells were cultured in the NSC medium for 3–5 days.

### phOCT4-EGFP reporter assays

The human *POU5F1* promoter (3,471 bp) and five exons (hOCT4pr, from 64,603 to 74,145 in human DNA sequence with accession number AP000509) was amplified using PCR with primers: hOCT4pr-F (5′-GGGGTACCATTTGTCTAAATTGCTATTAAGG-3′) and hOCT4pr-R (5′-CTAGCTAGCGGTGACCACTTCCCCATC-3′) using the genomic DNA of LO2 cell as a template. The fragment was cloned into the pEASY vectors (TransGen Biotech, CT111-01), and the fidelity of the DNA sequence was confirmed by bidirectional sequencing. The correct *POU5F1* fragment was subsequently cloned into vector pGL4.10 (Addgene, E6651) by insertion into KpnI and BglII restriction enzyme sites upstream of EGFP, so that the expression of the EGFP reporter gene was only driven by the OCT4 promoter. The derived plasmid was designated as the ‘phOCT4-EGFP'. Three mutants of the ‘phOCT4-EGFP' each targeting an AHRE motif at the *POU5F1* gene were further generated using reverse PCR amplification, using the following primers (with the mutated nucleotides underlined):

AHRE1-MUT F: 5′-AGTTGAAAGTTGGGTGTGGTGGCTGTGCCCTTTAATCATGA-3′,

AHRE1-MUT R: 5′-TGTCATGATTAAAGGGCACAGCCACCACACCCAACTTTCA-3′;

AHRE2-MUT F: 5′-GCAGTGGTTCTCAGTGGGATGCTCAGAAATTCCTCA-3′,

AHRE2-MUT R: 5′-CAGCAGAACTGAGGAATTTCTGAGCATCCCACTGA-3′.

AHRE3-MUT F: 5′-ACAAGGGCCCTCGCACCTCCCTCACTTTG-3′,

AHRE3-MUT R: 5′-AGTGAGGGAGGTGCGAGGGCCCTTGTGAC-3′.

The wild-type phOCT4-EGFP plasmid as well as its mutants were transfected into U87 cells according to the standard procedures using the SuperFectin II DNA Transfection Reagent (Shanghai Pufei Biotech, 2102-100, China). The treatment with ITE (10 μM) or RA (10 μM) started at the same time as the transfection. The *EGFP* transcripts were quantified using quantitative reverse transcription–PCR (qRT–PCR).

### U87 tumour sphere growth and transfection

For Oct4 overexpression and AHR siRNA assays, U87 cells were seeded in 6-cm dishes at a density of 200,000 cells per dish and allowed to attach overnight, and then transiently co-transfected with pEGFP plasmid plus Scr siRNA, phOCT4-EGFP plasmid plus Scr siRNA and phOCT4-EGFP plasmid plus AHR siRNA via the GenEscort™ transfection reagent. Twenty-four hours later, the transfected cells were trypsinized and plated into 6-cm dishes and 96-well plates in the NSC medium at a density of 200,000 cells per dish and 10,000 cells per well, respectively, and 10 μM ITE or 0.01% DMSO (vehicle) were replenished every 8 h for 72 h. After treatment, U87 tumour spheres in 6-cm dishes were harvested and subjected to immunoblot analysis and U87 tumour spheres in 96-well plates were counted under an Olympus IX81 microscope with an Olympus IX-TVAD camera (the cell aggregate with a diameter over 100 μm was counted as a typical tumour sphere).

### Photoactivation of L-tryptophan

L-tryptophan (Trp) was dissolved in distilled water at 100 × (1 × was defined as the concentration of Trp in DMEM, that is, 16 mg l^−1^), and the Trp solution was exposed to sunlight passing through a large east-facing window for 7 days in a 50-ml polypropylene tube as described^31^. Light-activated Trp is referred to as aTrp. The 100 × aTrp stock solution was wrapped in aluminium foil, kept at 4 °C and used within a week.

### Analysis of tryptophan derivatives using HPLC

L-tryptophan, ITE and other tryptophan derivatives in 8 × 10^7^ cells grown in Ham's F10, DMEM or NSC media were measured as described^6^,^32^. After centrifugation, 100 ml of the supernatant was dried under N_2_ and resuspended in 10 ml of methanol followed by washing with 5 ml methanol. The extract was evaporated to dryness using a rotavapor and further dissolved in 500 μl methanol. HPLC analyses and fractionations were performed using an Agilent 1200 series HPLC with SB-C18 column (250 mm × 4.6 mm ID, 5 μm, Agilent technologies). A 40-min linear mobile phase gradient from 60% B to 0% B (A: MeOH and B: 0.1% TFA in acetonitrile) was used at a flow rate of 0.8 ml min^−1^. Dual-wavelength ultraviolet detection was at 280 and 380 nm. Standard curves were generated with synthetic ITE and L-tryptophan in aqueous solutions.

### Lentiviral vector construction and viral infection

The open reading frame cDNA of *AHR* was cloned using PCR from the *AHR* plasmid (CCSB, Internal ID: 13956) and inserted into SacI–HindIII restriction sites of the plasmid PCHN2, modified from plasmid LV-rtTA-IRES-EGFP, generating the viral vector-based plasmid *Flag-AHR*. An shRNA fragment targeting the CDS of *AHR* was generated using a pair of primers (forward primer: 5′-CCGGGCAACAAGATGAGTCTATTTACTCGAGTAAATAGACTCATCTTGTTGCTTTTTG-3′ where the target sequence was underlined; reverse primer: 5′-AATTCAAAAAGCAACAAGATGAGTCTATTTACTCGAGTAAATAGACTCATCTTGTTGC-3′) and cloned into the plasmid pLKO.1-TRC (Addgene 10878) as described in the TRC protocols http://www.broadinstitute.org, and the resulting plasmid was designated as ‘*AHR* shRNA'.

Viral production and infection were performed following the TRC protocols (The RNAi Consortium, http://www.broadinstitute.org). Briefly, 293T cells were co-transfected with the above viral vector-based *AHR* plasmid together with lentivirus-packaging vectors pMD2.G (Addgene 12259) and psPAX2 (Addgene 12260) using SuperFectin II. Forty-eight and seventy-two hours after transfection, the culture supernatants containing the released viral particles were collected, filtered through 0.45-μm membranes (Millipore SCHVU01RE) and used freshly or stored at 4 °C for less than 2 days. For viral infection, cells were plated and cultured overnight, and viral supernatants were added with polybrene (Sigma AL-118) at a final concentration of 8 μg ml^−1^. In most cases, the multiplicity of infection was estimated to be between 0.5 and 2. After 6–10 h, the media were replaced with fresh viral-free medium to allow further growth until use.

### Electrophoretic mobility shift assay

U87 cells were treated with 1 or 100 μM ITE or DMSO (vehicle) in culture medium for 2 h, and/or infected with lentivirus harbouring *Flag-AHR* for 3 days. Nuclear extracts were prepared by a modified method described in ref. 33. Briefly, 2 × 10^6^ U87 cells were harvested, washed with phosphate-buffered saline (PBS) and pelleted at 500 g for 5 min. The supernatant was removed, and then 100 μl ice-cold CE buffer (10 mM HEPES, pH7.9, 1.5 mM MgCl_2_ and 10 mM KCl, with freshly added 0.075% NP40) was added to the cell pellet sequentially and centrifuged at 15,000*g* for 5 min at 4 °C. The resulting supernatant was designated as the cytoplasmic extract. The insoluble fraction containing the nuclei was resuspended with 50 μl ice-cold NE buffer and placed on ice for 60 min with intermittent shaking. The fraction was centrifuged at 15,000*g* for 10 min and resulting supernatant was designated as the nuclear extract and used for the following experiment.

EMSA was conducted using the LightShift chemiluminescent EMSA kit (Thermo 20148) according to the manufacturer's instructions. Nuclear extract (15 μg) was incubated with biotin-labelled WT or mutant (MUT) probe (4 μl, 50 nM). The sequences of double-strand biotin-labelled *POU5F1* probes (with the putative AHRE motifs being underlined and the mutated nucleotides marked in bold and italics) were as follows:

WT probe 1: 5′-Biotin-GTGTGGTGGCTCACGCCTTTAATCATGACA-3′

3′-CACACCACCGAGTGCGGAAATTAGTACTGT-5′

MUT probe 1: 5′-Biotin-GTGTGGTGGCTC***TGC***CCTTTAATCATGACA-3′

3′-CACACCACCGAG***ACG***GGAAATTAGTACTGT-5′

Probe 2: 5′-Biotin-CTCAGTGGGATGGAGTGAAATTCCTCAGTTCTGC-3′

3′-GAGTCACCCTACCTCACTTTAAGGAGTCAAGACG-5′

Probe 3: 5′-Biotin-GGGTCACAAGGGCCCTGCGTGCTCCCTCACTTT-3′

3′-CCCAGTGTTCCCGGGACGCACGAGGGAGTGAAA-5′

200-fold molar excess of unlabelled wild-type *POU5F1* probes (8 μl, 5 μM) was added as a competitor for the biotin-labelled probes. Overall, 2 μl 10 × binding buffer and 1 μl poly (dI·dC; 1 μg μl^−1^) were added to each reaction, and then ultrapure water added to give a total volume of 20 μl. Binding reactions were incubated at room temperature for 30 min. Five microlitres of nondenaturing 5 × loading buffer was then added to each binding reaction, and loaded on a 6% native polyacrylamide gel in 0.5 × TAE. The gel and a polyvinylidene difluoride (PVDF) membrane (Millipore IPVH00010) were sandwiched in a clean transfer unit and transferred at 380 mA for 45 min with cooled 0.5 × TAE. The PVDF membrane was dried by blotting with paper towels, and then crosslinked for 15 min with 312 nm ultraviolet light. The membrane was blocked with 20 ml pre-warmed 1 × blocking buffer for 45 min with gentle shaking, followed by 15 min incubation with 1:300 diluted streptavidin–horseradish peroxidase conjugate. The membrane was washed four times with 1 × washing buffer, incubated with the substrate equilibration buffer and substrate working solution for 5 min, respectively, and then detected using the Chemiluminescent Imagine System (Tanon-5200).

### Chromatin immunoprecipitation

ChIP analysis was performed as described previously^34^. U87 cells were treated with DMSO (vehicle) or 10 μM ITE in culture medium for 2 h, or infected with lentiviruses harbouring *Flag-AHR* for 3 days. Overall, 2 × 10^7^–5 × 10^7^ of the above treated cells were chemically crosslinked by the addition of 1% formaldehyde solution for 10 min at room temperature. The reaction was stopped by adding glycine to a final concentration of 125 mM. Cells were rinsed twice with cold PBS, harvested by a silicon scraper and resuspended in 1 ml FA lysis buffer (50 mM HEPES-KOH, pH 7.5, 140 mM NaCl, 1 mM EDTA, 1% Triton X-100, 0.1% sodium deoxycholate, 0.1% SDS, with freshly added 1% protease inhibitor cocktails). For xenograft tumour samples, tumours were excised, washed briefly with ice-cold PBS, chopped into small pieces with a razor blade and transferred into a tube containing 1–2 ml of cold PBS. The tissue clumps were further disaggregated, followed by crosslinking in 1% formaldehyde solution for 10 min at room temperature and stopping with 125 mM glycine. The crosslinked tissue samples were washed twice by low-speed centrifugation (∼6,000*g*, 4 °C, 5 min), ground in liquid nitrogen and centrifuged to remove the supernatant. The resulting tissue pellets were resuspended in 1 ml FA lysis buffer. Next, the above treated cell or tissue samples were sonicated to solubilize and shear crosslinked DNA to an average size of 300–800 bp. An aliquot of the lysates (100 μl) was used as a control for the amount of input DNA after being purified using the PCR clean-up kit (Axygen AP-PCR-50). Immunoprecipitation was carried out with 5 μg rabbit anti-AhR together with 100 μl protein A/G agarose beads (Pierce 20421) or 50 μl anti-Flag M2 magnetic beads (with 0.1 μg normal mouse IgG or 50 μl mouse IgG conjugated with magnetic beads being the negative control, respectively) for each sample at 4 °C overnight. Beads were washed three times with the RIPA buffer and finally with the TE buffer containing 50 mM NaCl. DNA fragments from each reaction were eluted with 200 μl elution buffer (1% SDS, 100 mM NaHCO_3_) supplemented with 5 μl of 10 mg ml^−1^ protease K and 2 μl of 100 mg ml^−1^ RNase A by incubation at 65 °C overnight, and purified by using the PCR clean-up kit. The primers used to amplify the DNA fragments are listed in Supplementary Information (Supplementary Table 1). qRT–PCR was performed with SYBR Green (Platinum SYBR Green qPCR SuperMix-UDG, Invitrogen 11733-038) using an MJ Chromo 4 (Bio-rad DNA Engine). The PCR cycle parameters were as follows: 94 °C for 2 min; 40 cycles with denaturation at 94 °C for 20 s, annealing at 60 °C for 20 s and extension at 72 °C for 20 s. All the PCR amplification was performed in triplicate and reproduced three times. The data were analysed using the SAS 9.1 software. The precipitated DNA fragments corresponding to specific genes were quantified using qRT–PCR and expressed as percentages of their total input DNA fragments. Besides the control IgG, the amount of *ACTB* and *POU5F1-nonAHRE* DNA fragment that was precipitated and analysed under same conditions served as an additional control for specificity of the binding between the ChIP antibodies and their target genes.

### qRT–PCR analysis

Total mRNA was isolated using TRIzol reagent. Reverse transcription reaction was performed using the PrimeScript RT reagent kit (TaKaRa RR037A) according to the manufacturer's instructions. All the primers used were listed in Supplementary Information (Supplementary Table 2). qRT–PCR was performed in a MJ Chromo 4 (Bio-Rad DNA Engine) by using a reaction mixture with Platinum SYBR qPCR SuperMix-UDG (Invitrogen 11733-038). The PCR cycle parameters were as follows: 94 °C for 2 min; 40 cycles with denaturation at 94 °C for 20 s, annealing at 60 °C for 20 s and extension at 72 °C for 20 s. All the PCR amplification was performed in triplicate and repeated in three independent experiments. The relative quantities of selected mRNAs were normalized to that of GAPDH, and the derived values of ITE-treated or AhR-infected samples were further normalized to those of vehicle-treated samples, with the latter being set as 1, or in other cases, relative quantities of selected mRNAs in the 10 cell lines were normalized to that of *GAPDH*, and the derived values of nine cell lines were further normalized to that of HeLa cells, with the latter being set as 1. In most cases, the data were expressed as mean±s.d. of triplicate measurements from one of three independent experiments, which gave similar results. The statistical analyses were performed with either the Student's *t*-test or analysis of variance using the SAS 9.1 software, and *P*≤0.05 was considered statistically significant (*P<0.05, **P<0.01, ***P<0.001).

### Immunoblot analysis

Cultured cells with or without treatment were lysed on ice in Lysis Buffer (20 mM Tris-HCl, pH 7.5, 150 mM NaCl, 1 mM Na_2_EDTA, 1 mM EGTA, 1% Triton X-100, 2.5 mM sodium pyrophosphate, 1 mM beta-glycerophosphate, 1 mM Na_3_VO_4_, 1 μg ml^−1^ leupeptin, Cell Signaling Technology 9803) supplemented with Complete Protease Inhibitor Cocktail (Santa Cruz Biotechnology sc-29130) and phosphatase inhibitors (PhosSTOP, Roche 04906845001). For xenografted tumour samples, ∼1 g of the tumour tissue was ground in liquid nitrogen and transferred to a clean Eppendorf tube. The tissue pellets were resuspended in 600 μl of the Lysis Buffer, mixed gently on ice for 30 min and centrifuged at 14,000*g* for 10 min at 4 °C. The supernatant containing the whole-tumour tissue lysate was transferred to a fresh tube for further analyses. The protein concentrations of the lysates were measured using a BCA Assay kit (Thermo 23227). The whole-cell/tissue lysates were boiled with SDS–PAGE sample loading buffer, separated by SDS–PAGE, blotted on PVDF or nitrocellulose membranes and probed with the indicated antibodies. The signals were visualized using the Immobilon Western Chemiluminescent HRP Substrate (Millipore WBKLS0100). The uncropped blots for main figures were presented in Supplementary Fig. 42.

### Cell proliferation assays

Cell proliferation was determined with the Cell Proliferation Kit I (MTT; Roche 11465007001) according to the manufacturer's instructions. Briefly, U87 cells grown in a 96-well plate (3,000–10,000 cells per well) were treated with DMSO (vehicle) or reagents for specified durations. Ten microlitres of the MTT labelling reagent (final concentration 0.5 mg ml^−1^) was added into each well, and the plate was incubated for 4 h at 37 °C with 5% CO_2_. The absorbance was measured using a microplate reader (Beckman Coulter DTX880) at the wavelength of 570 nm.

### Mouse xenograft tumour models

BALB/c nude mice (female, 3–4 weeks old) were purchased from Shanghai Experimental Animal Centre, Chinese Academy of Science. Experimental animals were kept in the central animal facility of the Zhejiang University School of Medicine and housed in laminar-flow cabinets under specific pathogen-free conditions with a 12 h light/dark cycle. All studies on mice were conducted in accordance with the National Institute Guide for the Care and Use of Laboratory Animal. The animal protocol has been approved by the Committee of the Ethics of Animal Experiments of the Zhejiang University.

For subcutaneous xenografting experiments, U87 cells (2.5 × 10^6^), HepG2 cells (1 × 10^7^) or LNCap cells (2 × 10^6^) were inoculated subcutaneously into each mouse. When the tumour volume reached ∼100 mm^3^, the mice were divided into homogenous blocks based on their tumour volumes followed by randomly assigning each block into the vehicle control and ITE treatment groups (*n*=8–13 per group). The vehicle (DMSO) or ITE was administered to the mice by intratumoral injection (5 μl of 80 mg ml^−1^ ITE diluted in 10 μl of PBS) once daily for 3 consecutive days in short-term experiments, or by intraperitoneal injection (80 mg kg^−1^ ITE in most cases) once daily for 18–33 consecutive days in long-term experiments. The external diameter of the tumours was measured as described previously^17^.

For orthotopic xenograft model, the HCCLM3-RFP cell line was established by transfecting the HCCLM3 cells with LV-7 RFP plasmid (GenePharma, Shanghai). HCCLM3-RFP cells (1 × 10^7^ cells in 0.1 ml PBS) were inoculated to form ectopic transplanted carcinoma in 4-week-old male BALB/c nude mice by subcutaneous injection. Subcutaneous tumours were harvested when they reached 1 cm in diameter and were cut into pieces under aseptic conditions. After washing with PBS, the tumours were rinsed in DMEM. Only intact pieces were chosen for further study. After removing thanatosis tissues, the tumours were cut into cubes 1 mm^3^ in size. One piece was then implanted into the left liver lobe of each mouse as previously described^35^. ITE administration started 20 days after implantation, at which time the sizes of implanted HCCLM3-RFP cells were roughly estimated by the *in vivo* imaging system. Each treatment group consisted of four tumour-bearing mice. DMSO (vehicle) or ITE dissolved in the vehicle at 80 mg per kg body weight were administered to the mice by intraperitoneal injection once daily for 21 consecutive days. After *in vivo* images were acquired, the mice were then killed. Following tumour excision from the killed mice, a portion of the tumour tissue was dissected and analysed using qRT–PCR and immunoblotting.

### Immunofluorescence microscopy

Cells were washed twice with PBS and fixed with 4% formaldehyde (Sigma F8775-25ML) in PBS for 30 min at room temperature, washed again three times with PBS and permeabilized with 0.1% Triton X-100 in PBS at room temperature for 10 min. They were then washed three times with PBS and blocked with 3% BSA (Sigma A9418) in PBS for 1 h. Thereafter, cells were incubated with a primary antibody and a secondary antibody for 1 h with three washes in between. Nuclei were counterstained with 4,6-diamidino-2-phenylindole or Hoechst 33342. Images were acquired using an Olympus IX81 inverted fluorescence microscope equipped with an Olympus 1 × 2-UCB camera or the Zeiss LSM 710 system. The AhR fluorescence intensity ratio between the nucleus and cytoplasm was analysed with ImageJ.

### DNA microarray

Briefly, RNA was extracted from U87 parental and tumour sphere cells using the TRIzol method according to the manufacturer's instructions. Reverse transcription to the first-strand cDNA was primed with T7 oligo(dT) primer to synthesize cDNA containing a T7 promoter sequence. Second-strand cDNA synthesis converts the single-stranded cDNA into a double-stranded DNA template for transcription. The reaction employs DNA polymerase and RNase H to simultaneously degrade the RNA and synthesize the second-strand cDNA. *In vitro* transcription to synthesize biotin-modified aRNA with IVT Labeling Master Mix generates multiple copies of biotin-modified aRNA from the double-stranded cDNA templates. aRNA purification removes unincorporated NTPs, salts, enzymes and inorganic phosphate to improve the stability of the biotin-modified aRNA. Fragmentation of the labelled aRNA prepares the target for hybridization to GeneChip 3′ expression arrays. Above procedures were carried out using GeneChip IVT express Kits (Affymetrix: 901229). The hybridization was carried out using GeneChip Hybridization, Wash and Stain kit (Affymetrix: 900720). The arrays were scanned at 570 nm with a confocal scanner from Affymetrix. Analysis of the arrays was performed using the Partek GS 6.5. Normalization of the array was performed using a robust multiarray analysis. A *P-*value cut off of 0.05 was used to filter genes that were significantly expressed between the two samples. A fold change of ≥2 was used as a criterion for primary selection for differentially expressed genes. The selected genes were further filtered by the following formula to get the final hits: the absolute value of (ratio of U87 sphere versus U87 parental−1)/(ratio of U87 sphere ITE versus U87 parental−1)≥1.5. Histograms of upregulated genes were prepared using the GraphPad Prism 5.0 software. The primary data can be accessed at the Gene Expression Omnibus with the accession code of GSE67986.

### Oncomine data acquisition and analysis

To explore the correlation of *AHR* and *POU5F1* mRNA levels in an expanded panel of human cancer cell lines, we conducted an *in silico* search of the Oncomine database ( www.oncomine.org) filtered by ‘Data Type: mRNA' and ‘Data set Type: Cell Line Panel Data sets'. Among the available data sets, the Garnett Cell Line (732) data set that contains data of 732 cell line samples' transcription profile was selected as the data source. The data of *AHR*, *POU5F1*, *CYP1A1* and *CYP1B1* mRNA levels (log2 median-centred intensity) in 24 human cancer cell lines were collected and analysed using Spearman's correlation analysis method using the SPSS 19.0 statistical software package (IBM Corporation, NY, USA).

## Additional information

**Accession codes**: The microarray data have been deposited in the Gene Expression Omnibus under accession code GSE67986.

**How to cite this article**: Cheng, J. *et al.* Tryptophan derivatives regulate the transcription of Oct4 in stem-like cancer cells. *Nat. Commun.* 6:7209 doi: 10.1038/ncomms8209 (2015).

## Supplementary Material

Supplementary Information Supplementary Figures 1-42, Supplementary Tables 1-2

Supplementary Dataset 1 Comparison of gene expression levels in parental U87 cells and U87 tumor spheres treated with or without ITE
