# Supplementary material for: Tryptophan derivatives regulate the transcription of Oct4 in stem-like cancer cells
Source: Nat Commun. 2015 Jun 10;6:7209. doi: 10.1038/ncomms8209 (PMC4490363; doi:10.1038/ncomms8209)
Supplement: Supplementary Information — Supplementary Figures 1-42, Supplementary Tables 1-2 [file ncomms8209-s1.pdf]

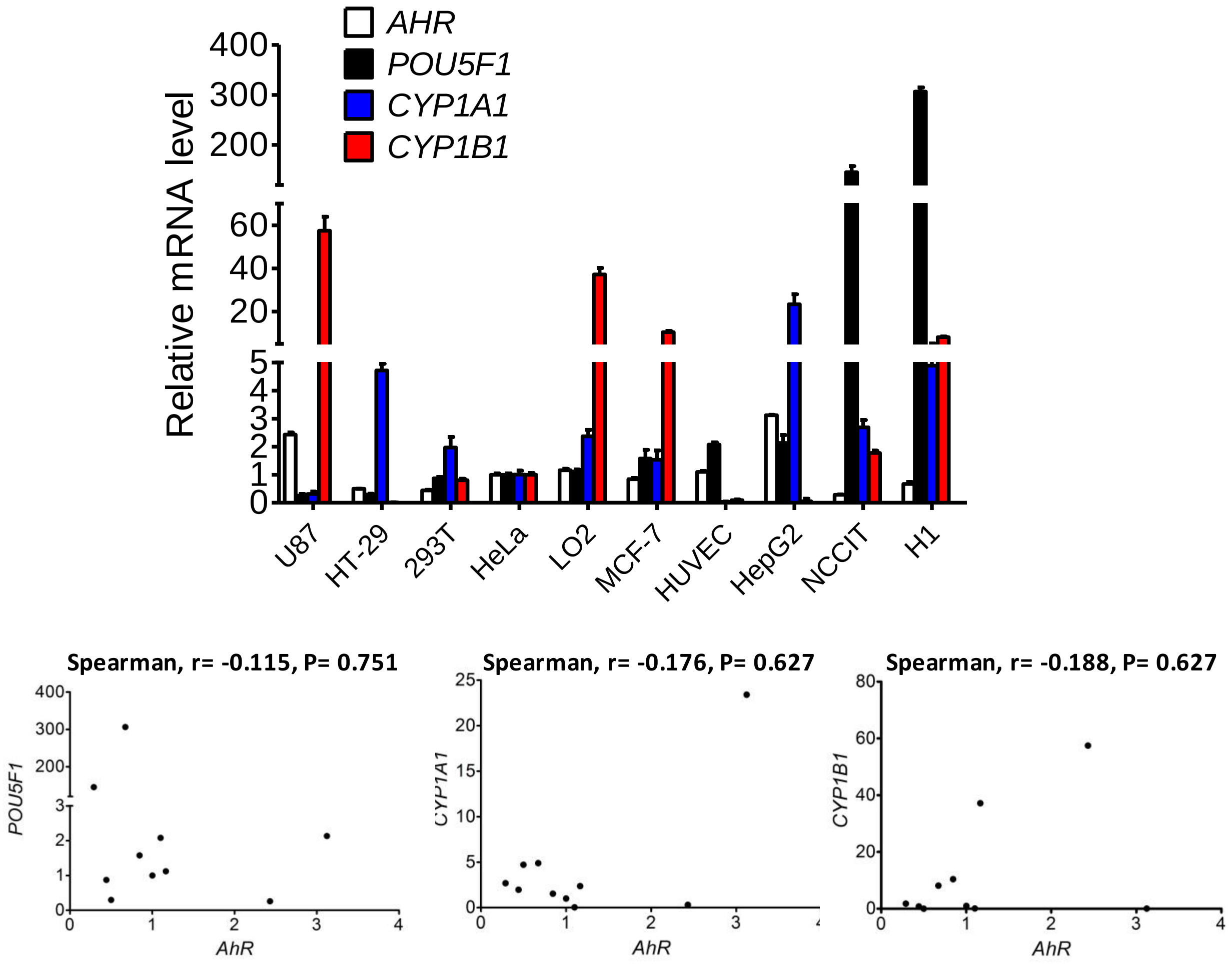

Supplementary Fig. 1. The mRNA levels of *AHR* and *POU5F1* and their correlations in human cell lines.

Upper panel: the mRNA levels of *AHR*, *POU5F1*, *CYP1A1* and *CYP1B1* in 10 human cell lines were determined by qRT-PCR with their levels in HeLa cells being set as 1. The data were expressed as mean ± SD of triplicate measurements from one of three independent experiments which gave similar results. Lower panels: the correlations between *AHR* and *POU5F1*, *AHR* and *CYP1A1*/*CYP1B1* were analyzed with the Spearman's correlation analysis method using SPSS 19.0 statistical software package.

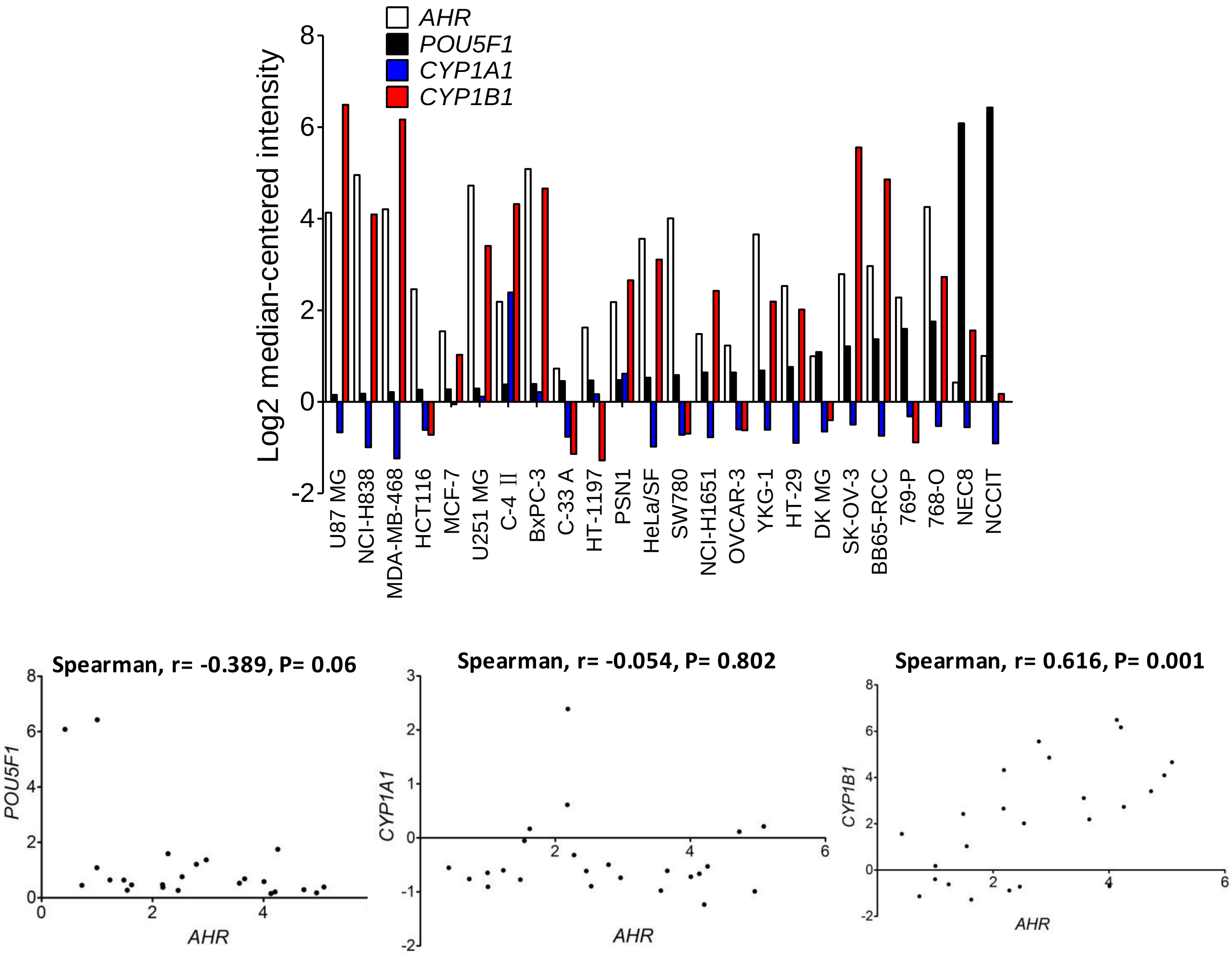

Supplementary Fig. 2. The mRNA levels of *AHR* and *POU5F1* and their correlations in expanded human cancer cell lines.

The mRNA levels of *AHR*, *POU5F1*, *CYP1A1* and *CYP1B1* (expressed in the form of log2 median-centered intensity) of 24 human cancer cell lines were obtained from the Garnett Cell line dataset (732) of Oncomine. The correlations between *AHR* and *POU5F1*, *AHR* and *CYP1A1*/*CYP1B1* were analyzed with the Spearman's correlation analysis method using SPSS 19.0 statistical software package.

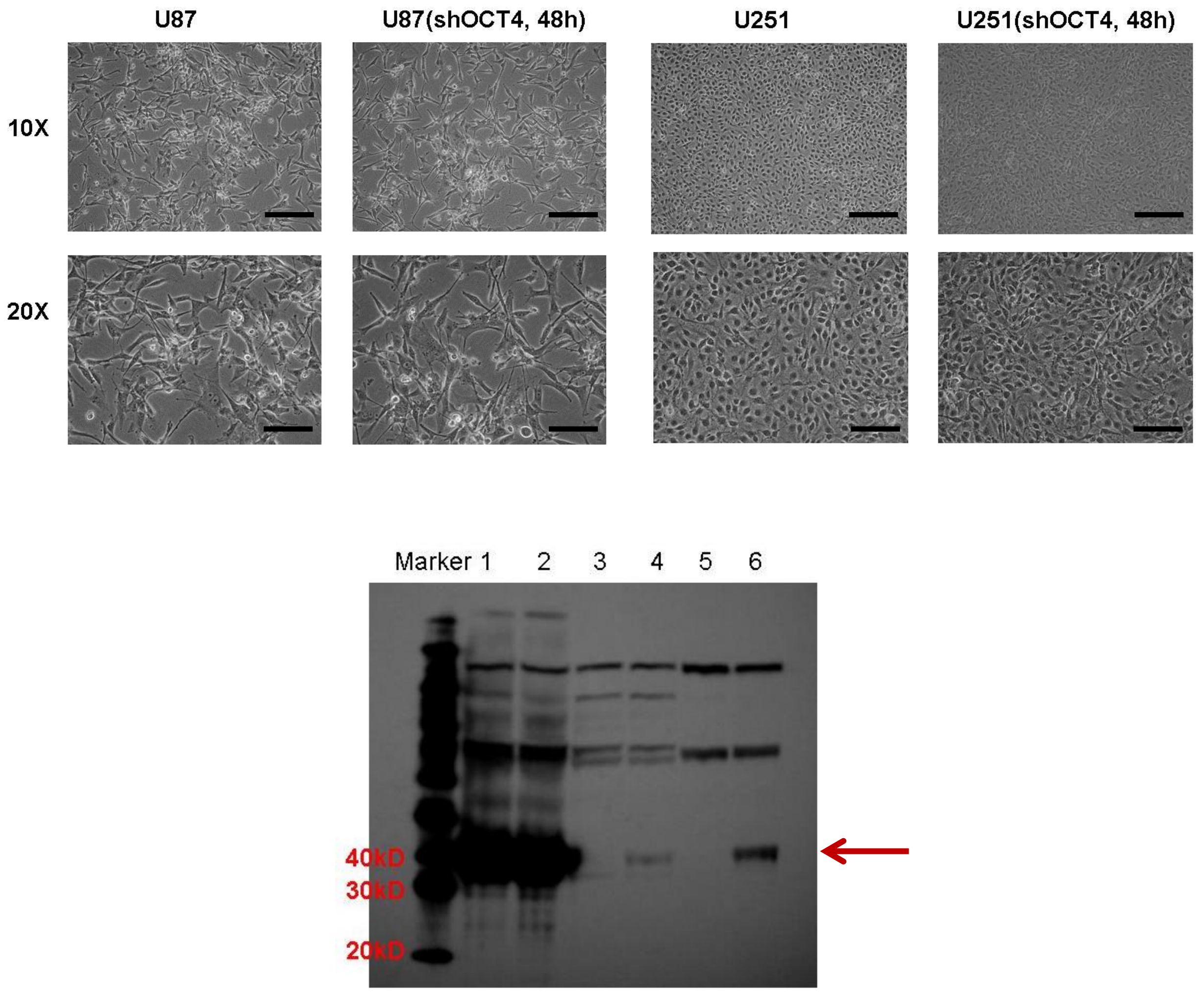

Supplementary Fig. 3. Specific knock-down of endogenous Oct4 in U87 and U251 cells.

U87 or U251 cells were seeded at a density of 2 x10^5 per well of the 6-well plate, and infected with lentiviruses harboring an shRNA against *POU5F1*. Cells were visualized by light microscopy 48 h post infection (upper panels, scale bars 100 and 50 μm, respectively), and harvested 72 h post infection for immunoblotting with anti-Oct4 (lower panel). Lanes 1 and 2 were NCCIT cells as positive control, lanes 4 and 6 were U251 and U87 cells with mock treatment respectively, lanes 3 and 5 were U251 and U87 cells treated with *POU5F1* shRNA respectively. The Oct4 band is indicated by the arrow.

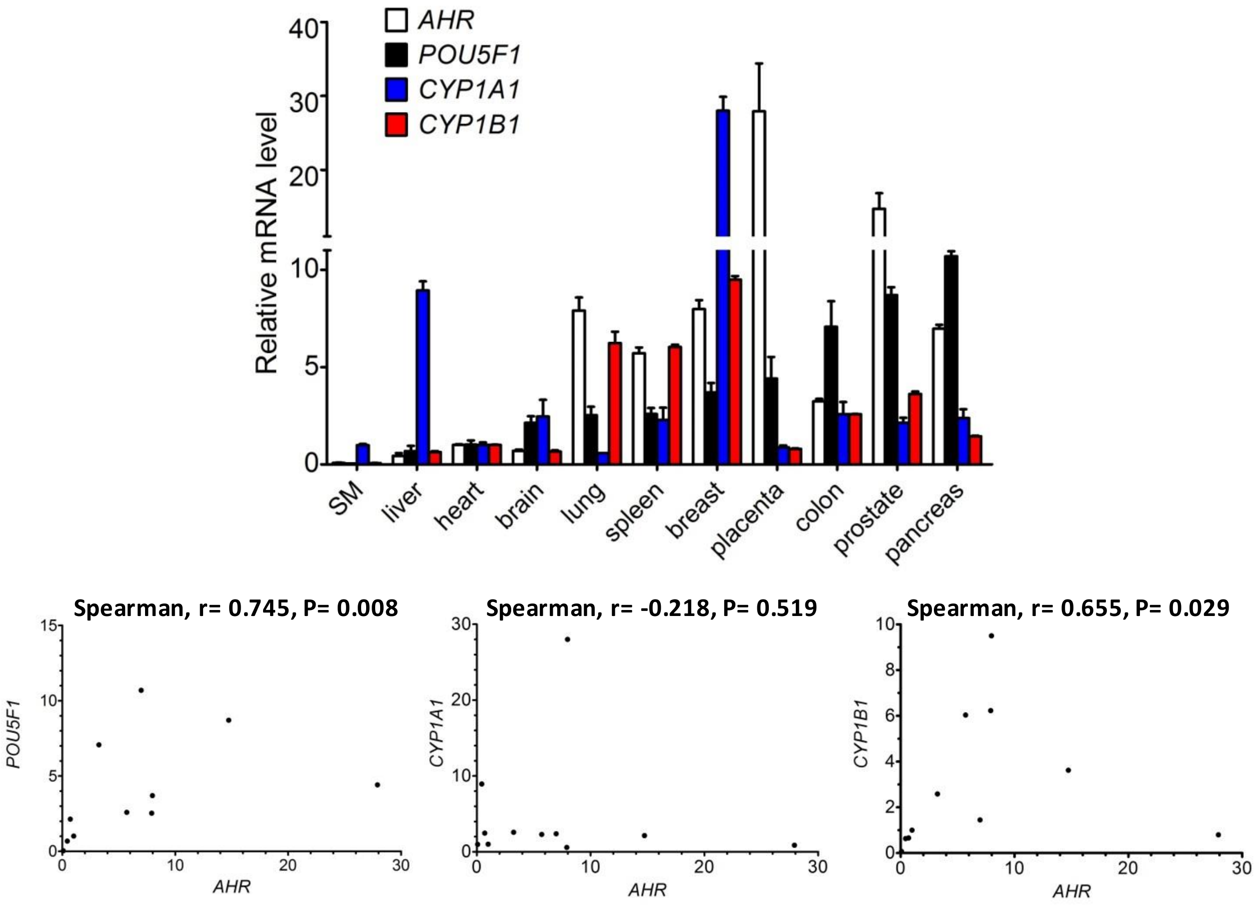

Supplementary Fig. 4. The mRNA levels of *AHR* and *POU5F1* and their correlations in normal human tissues. Upper panel: the mRNA levels of *AHR*, *POU5F1*, *CYP1A1* and *CYP1B1* in normal human tissues (SM: smooth muscle) were determined by qRT-PCR with their levels in heart being set as 1. The data were expressed as mean ± SD of triplicate measurements from one of three independent experiments that gave similar results. Lower panels: the correlations between *AHR* and *POU5F1*, *AHR* and *CYP1A1*/*CYP1B1* were analyzed with the Spearman's correlation analysis method using SPSS 19.0 statistical software package.

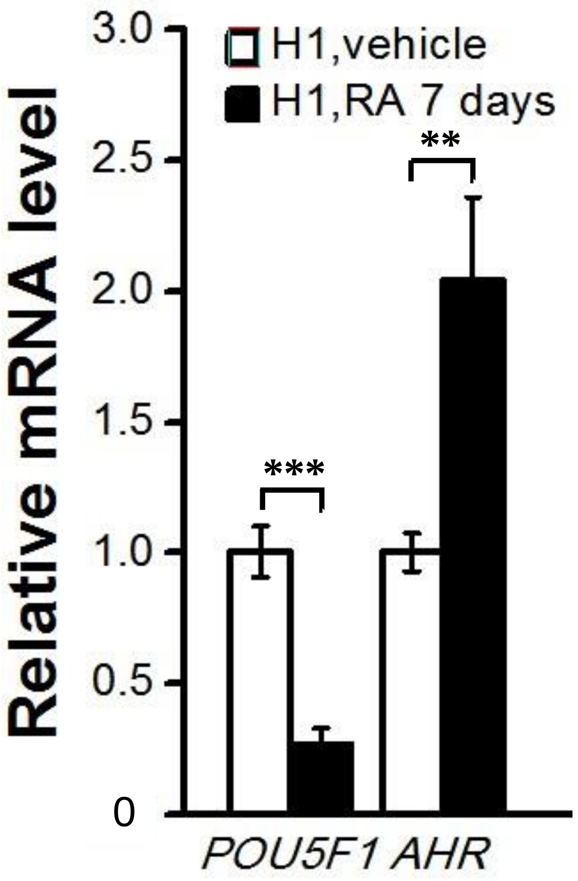

Supplementary Fig. 5. Changes of *AHR* and *POU5F1* mRNA levels during the differentiation of human ESCs. Increased *AHR* and decreased *POU5F1* mRNA levels during 10 μM RA-induced differentiation of H1 cells over 7 days, determined by qRT-PCR. The data were expressed as mean ± SD of triplicate measurements from one of three independent experiments which gave similar results. $^{**}P<0.01$, $^{***}P<0.001$.

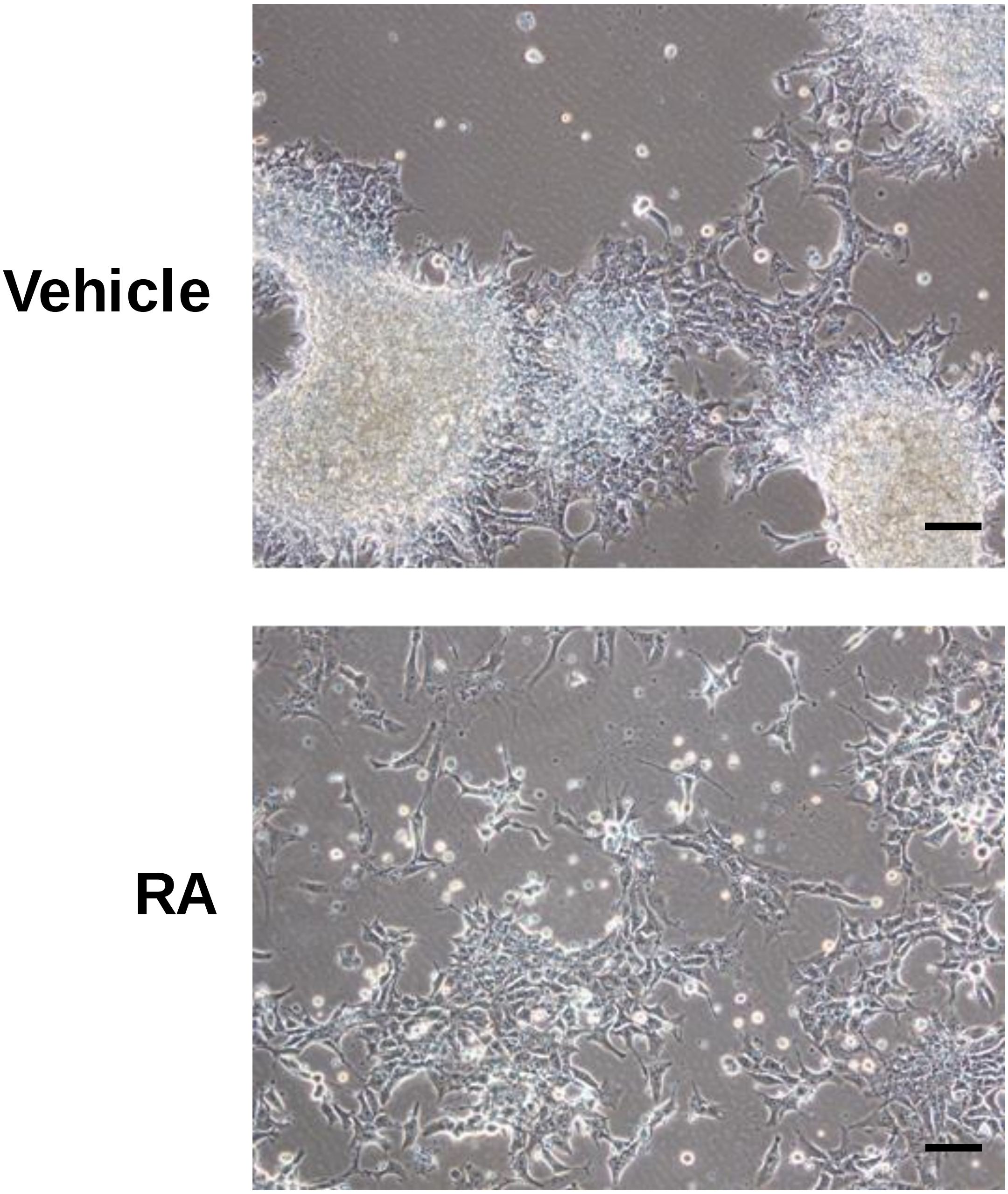

Supplementary Fig. 6. Morphological changes during the differentiation of NCCIT cells.

Morphologies of NCCIT cells treated with vehicle (DMSO) or 10 μM retinoic acid (RA) for 4 days, respectively. The compact colonies widely present in vehicle-treated cells disappeared largely in RA-treated cells. Scale bars, 100 μm.

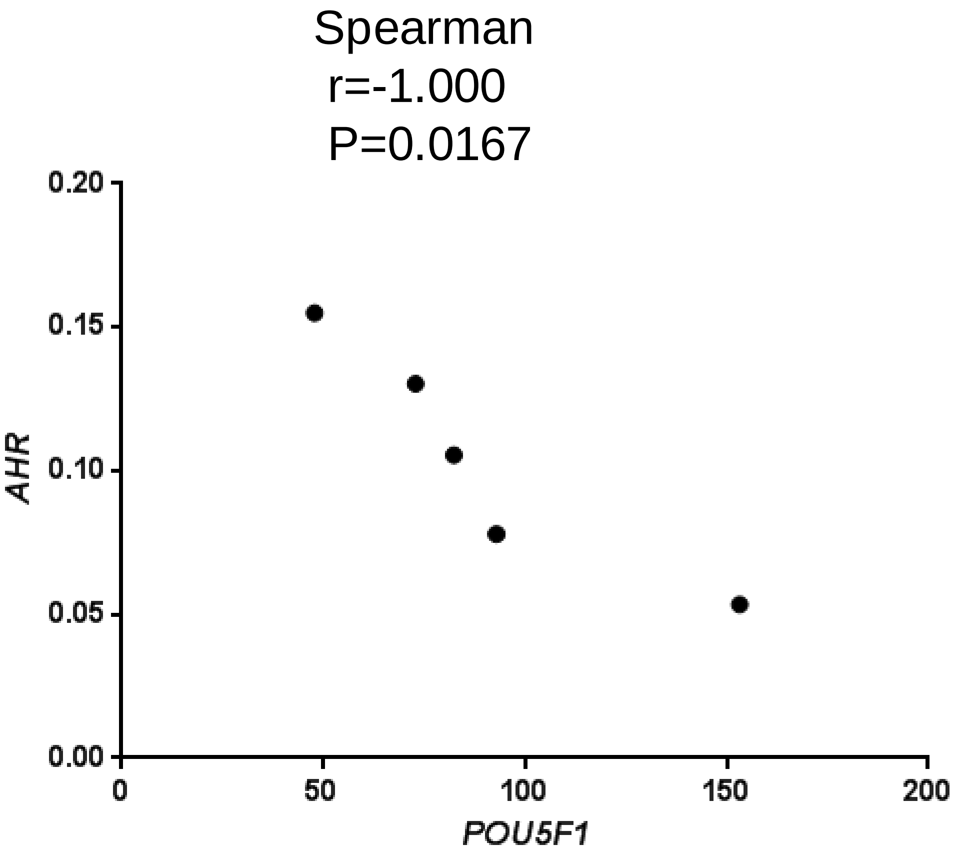

Supplementary Fig. 7. Negative correlation between the *AHR* and *POU5F1* mRNA levels during RA-induced differentiation of NCCIT cells.

The correlation between *AHR* and *POU5F1* was analyzed with the Spearman's correlation analysis method using SPSS 19.0 statistical software package.

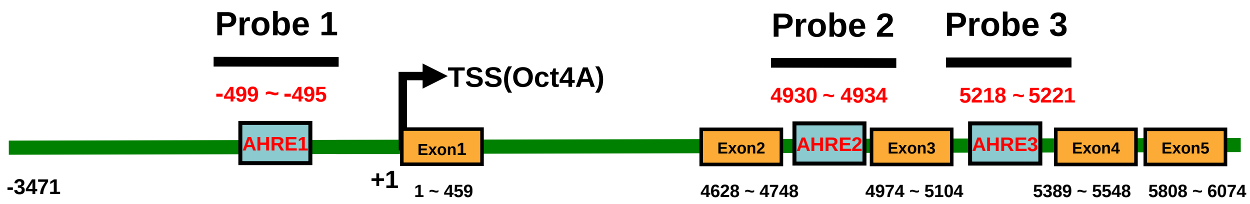

Supplementary Fig. 8. The schematic representation of three predicted AHREs in human *POU5F1* gene.
AHRE: AhR-responsive element. Three biotin-labeled probes spanning each of the AHREs respectively were used for EMSA shown in Fig. 1d.

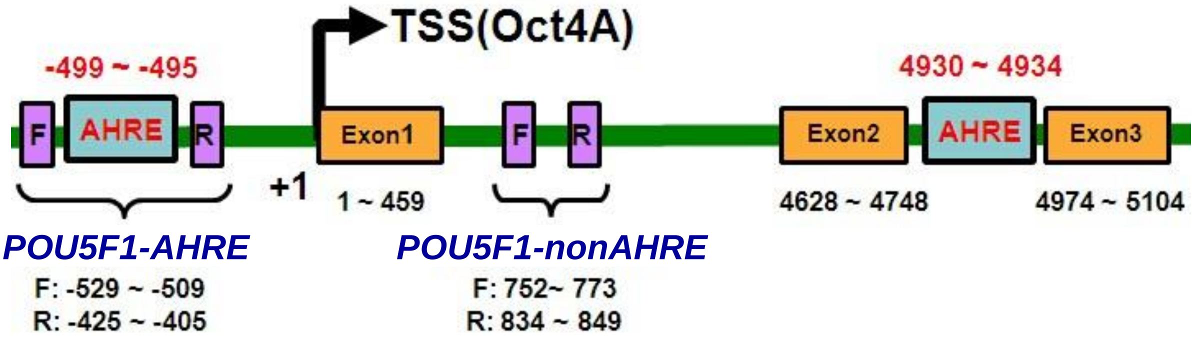

Supplementary Fig. 9. Primers for ChIP experiments.
The schematic representation of PCR primers and their amplified DNA fragments for ChIP experiments in Figures 1e, 2i, 4e, Supplementary Figures 25 and 37. DNA fragments immunoprecipitated by either anti-Flag or anti-AhR are PCR amplified with a pair of primers (purple frames, F: forward primer, R: reverse primer) amplifying a target region (-529 ~ -405 relative to the transcription start site (TSS)) of the *POU5F1* that harbors the presumed AHRE sequence, and the PCR products are designated as "*POU5F1-AHRE*". Meanwhile, the same DNA fragments are amplified with another pair of primers (752 ~ 849 relative to TSS) that do not target any AHRE and are distant from the three predicted AHREs. The corresponding PCR products are designated as "*POU5F1-nonAHRE*" and served as a negative internal control for the "*POU5F1-AHRE*" signals. More details about the *POU5F1* gene are given in Supplementary Fig. 8.

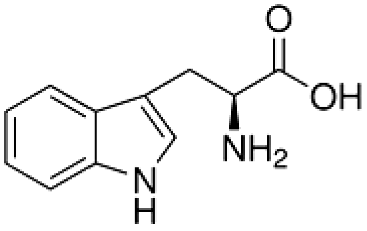

Typtophan

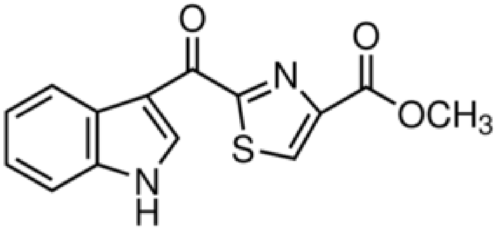

ITE

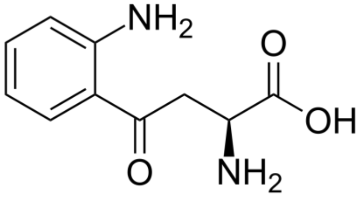

Kyn

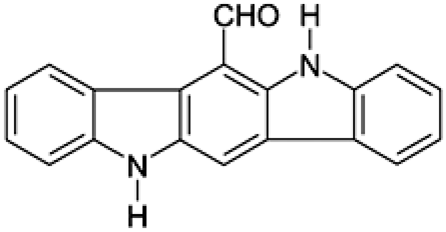

FICZ

Supplementary Fig. 10. Structures of tryptophan and its endogenous derivatives.

ITE: 2-(1’ H-indole-3’ -carbonyl)-thiazole-4-carboxylic acid methyl ester, Kyn: L-Kynurenine, FICZ: 6-formylindolo [3,2-b]carbazole

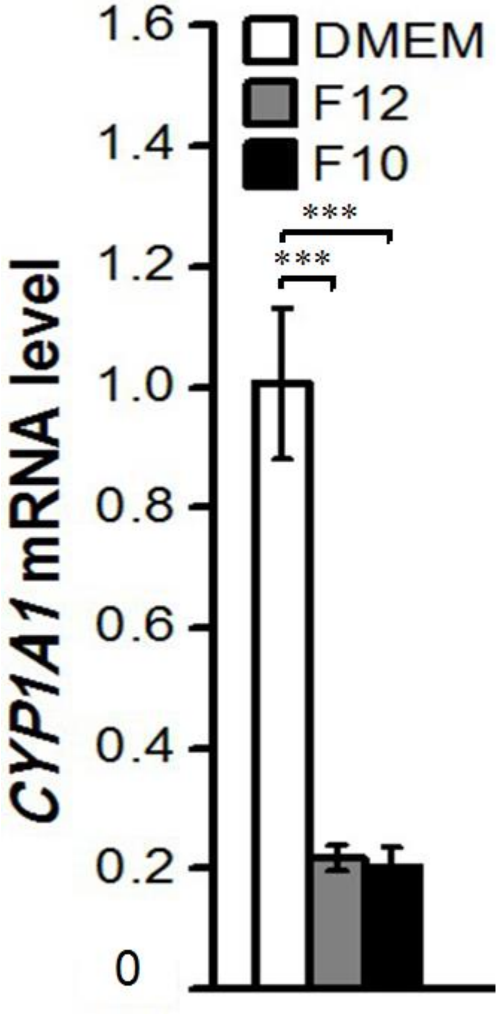

Supplementary Fig. 11. ***CYP1A1*** **mRNA levels in U87 cells cultured with different tryptophan concentrations.** U87 cells grown in tryptophan-rich medium (DMEM) or low-tryptophan medium (F12 and F10) for varying time were harvested. *CYP1A1* mRNA levels were determined by qRT-PCR and expressed relative to *GAPDH* mRNA. The data were expressed as mean ± SD of triplicate measurements from one of three independent experiments which gave similar results. ****P*<0.001.

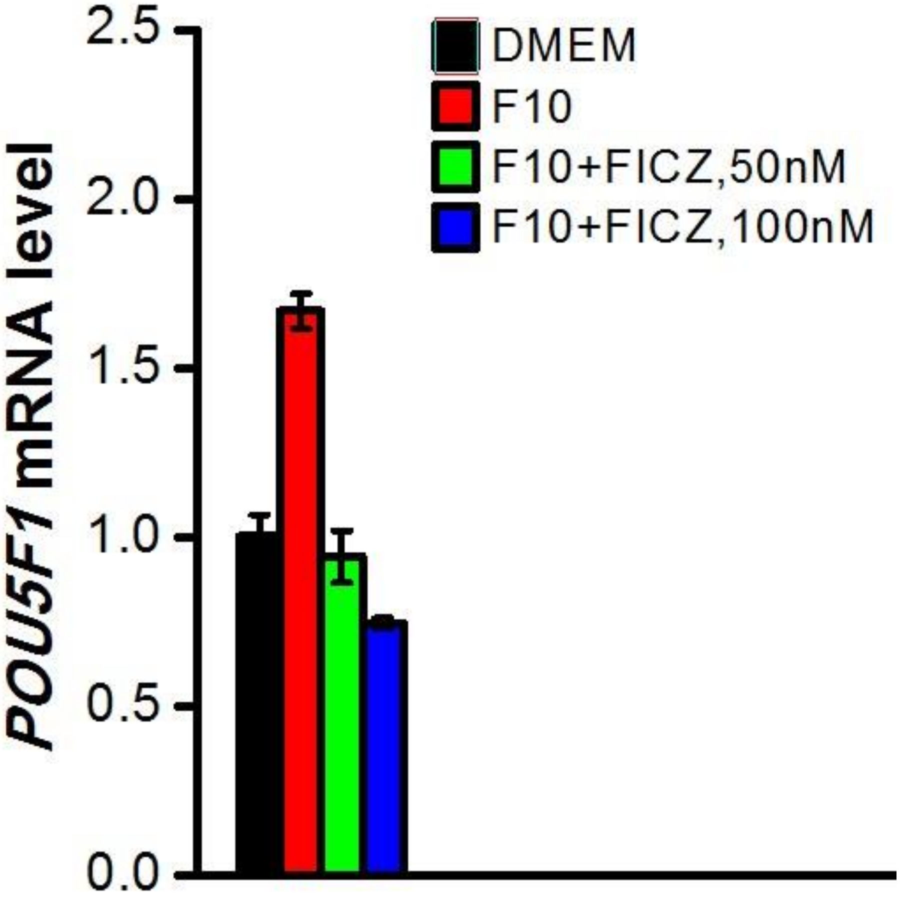

Supplementary Fig. 12. Effects of FICZ on *POU5F1* mRNA levels in U87 cells.

POU5F1 mRNA abundance in U87 cells cultured in DMEM or F10 medium supplemented with vehicle, 50 nM or 100 nM FICZ for 8 hours, respectively. *POU5F1* mRNA levels were determined by qRT-PCR and expressed relative to *GAPDH* mRNA. The data were expressed as mean ± SD of triplicate measurements from one of three independent experiments which gave similar results.

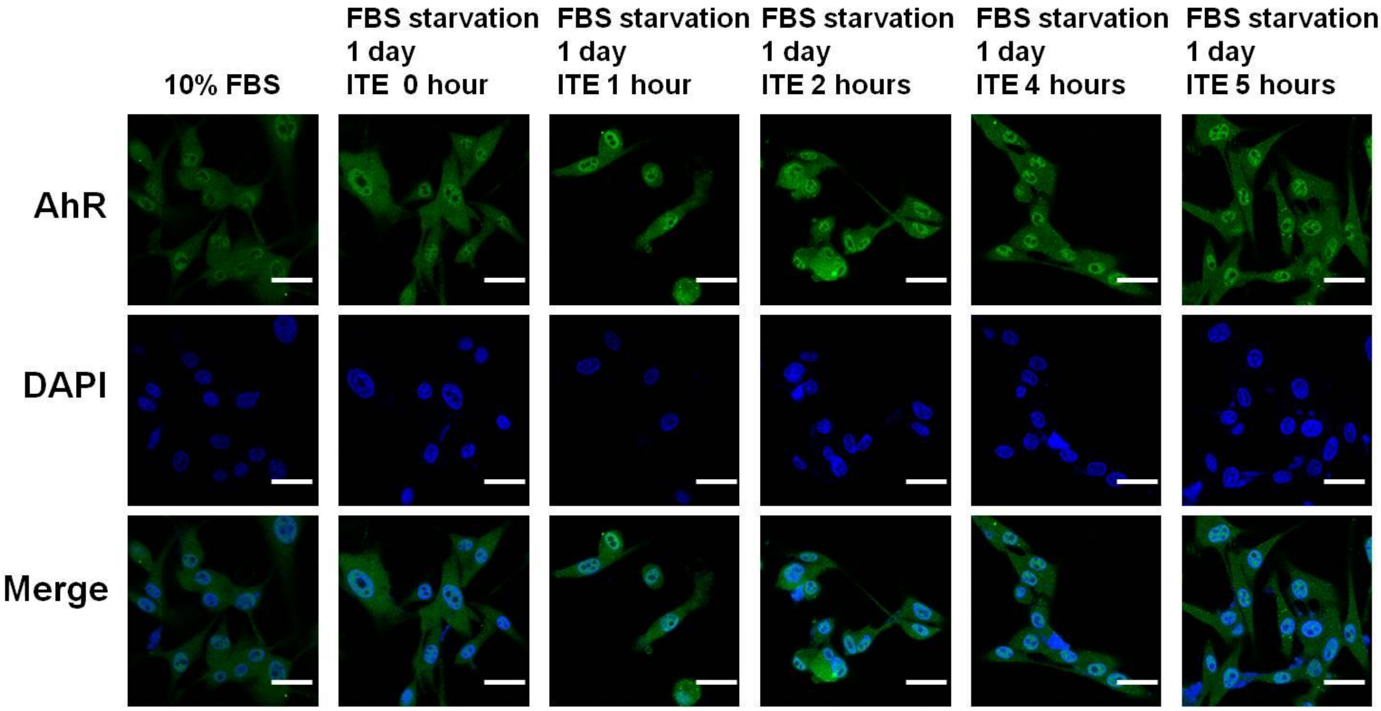

Supplementary Fig. 13. Localization of endogenous AhR in U87 cells.

U87 cells were grown in culture medium with or without 10% FBS for 1 day, followed by treatment with 10 μM ITE for 0, 1, 2, 4, and 5 hours, respectively. U87 cells were immunostained by anti-AhR antibody, and counterstained with DAPI. Scale bars, 50 μm.

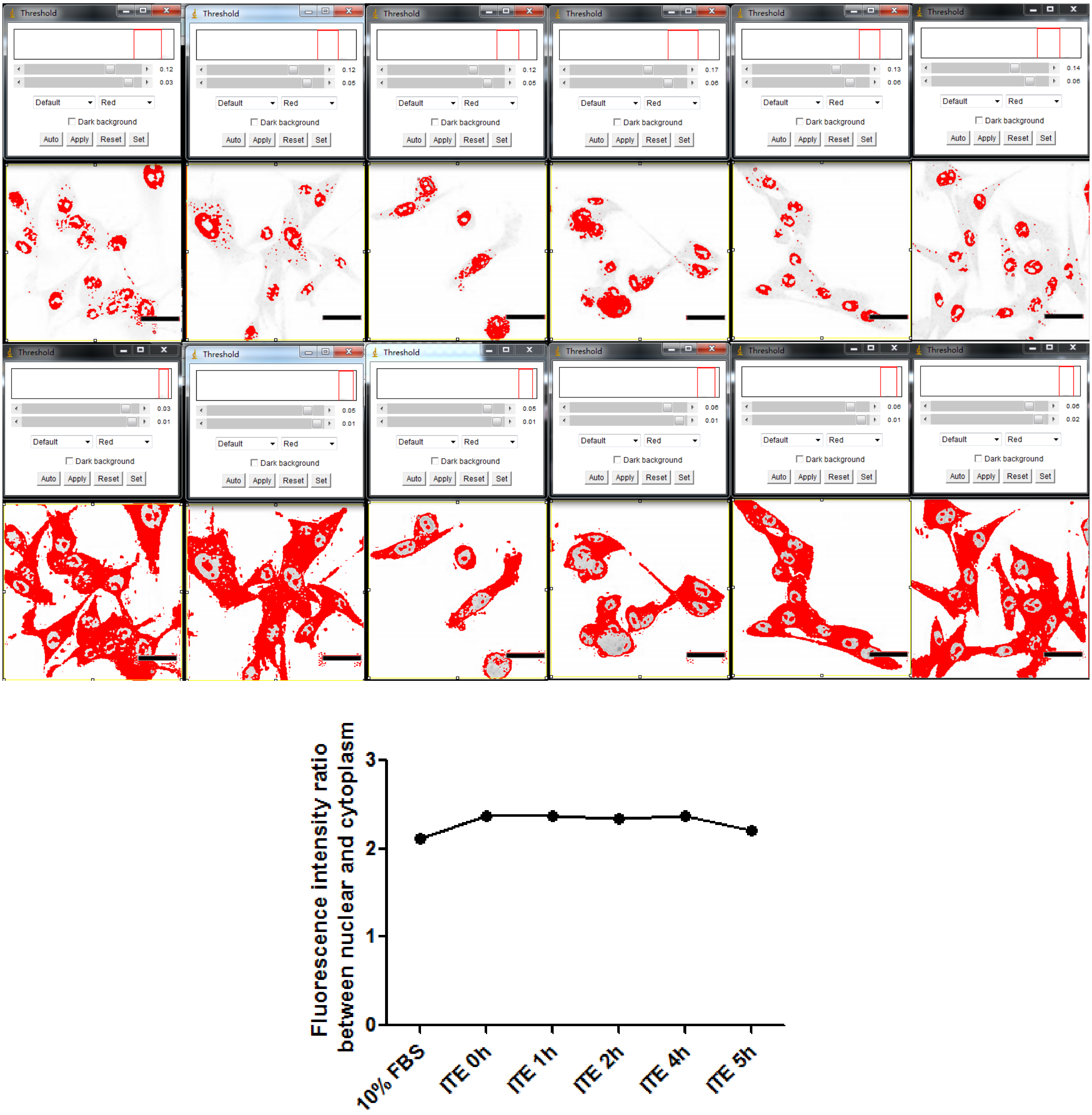

Supplementary Fig. 14. Determination of nuclear vs. cytoplasmic AhR levels.

AhR fluorescence intensity ratio between nucleus and cytoplasm was analyzed by ImageJ (upper panels). The lower panel is the quantification of AhR fluorescence intensity ratio between nucleus and cytoplasm.

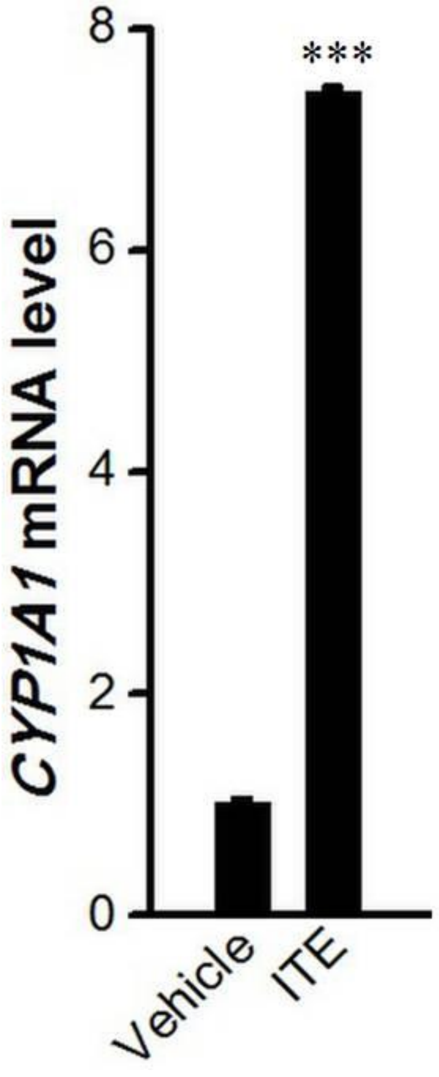

Supplementary Fig. 15. Effects of ITE on *CYP1A1* mRNA levels in U87 cells.

CYP1A1 mRNA abundance in U87 cells treated with vehicle or 10 μM ITE for 8 hours. *CYP1A1* mRNA levels were determined by qRT-PCR and expressed relative to *GAPDH* mRNA. The data were expressed as mean ± SD of triplicate measurements from one of three independent experiments which gave similar results. ***P<0.001.

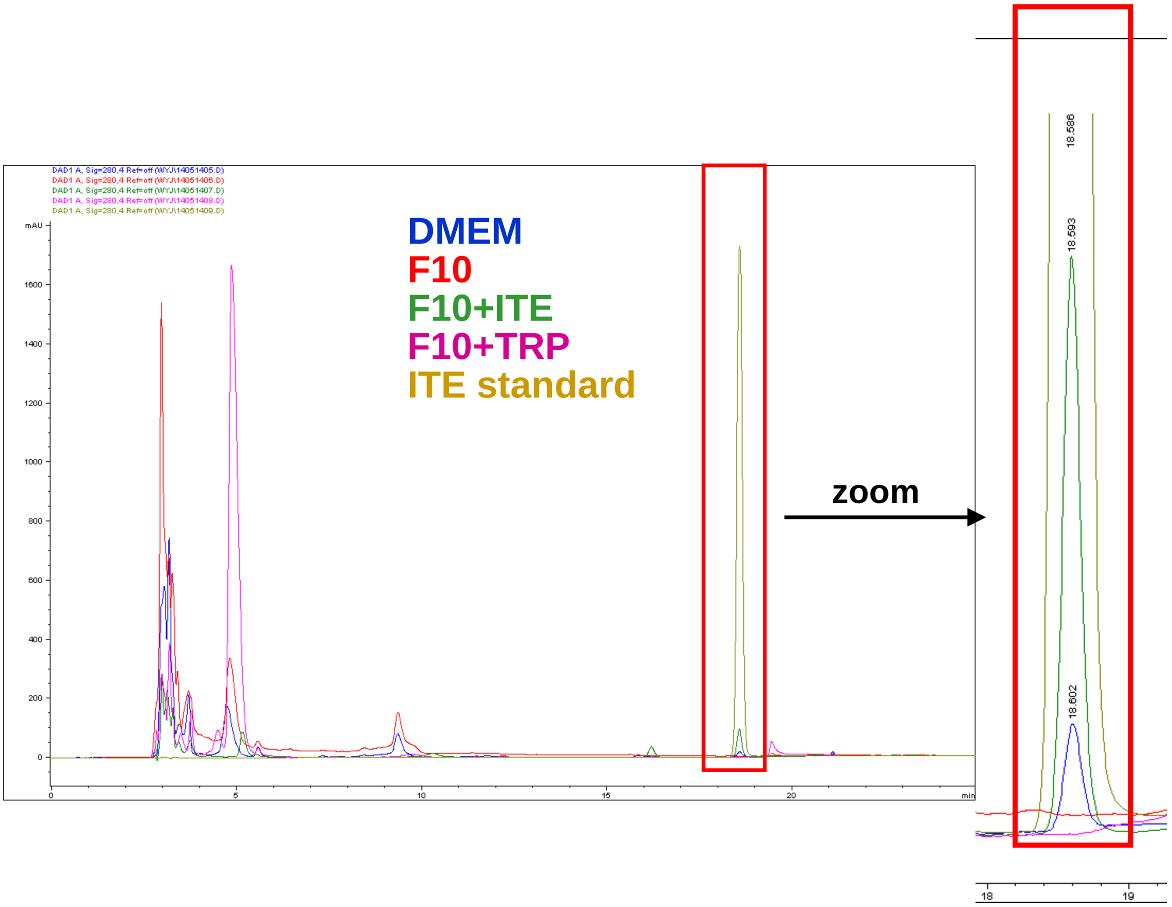

Supplementary Fig. 16. Identification of ITE and its derivatives in U87 cell cultures.

Reverse phase HPLC elution profile of metabolites of U87 cells cultured in DMEM or F10 medium supplemented with vehicle, 10 μM ITE, or 1 μM tryptophan (TRP) for 8 hours. Purified synthetic ITE was added as a reference sample. The peak at 18.6 min of retention time presumably corresponds to ITE. The apparent endogenous ITE peak area in DMEM group (blue line) and the synthetic ITE peak area in F10+ITE group (green line) were approximately 1/100 and 1/20 of that of the synthetic ITE standard (10 μM, brown line). Since the recovery rate during HPLC sample preparation was ~ 20%, the actual ITE concentration in DMEM and F10+ITE group was therefore 0.5 μM and 2.5 μM, respectively.

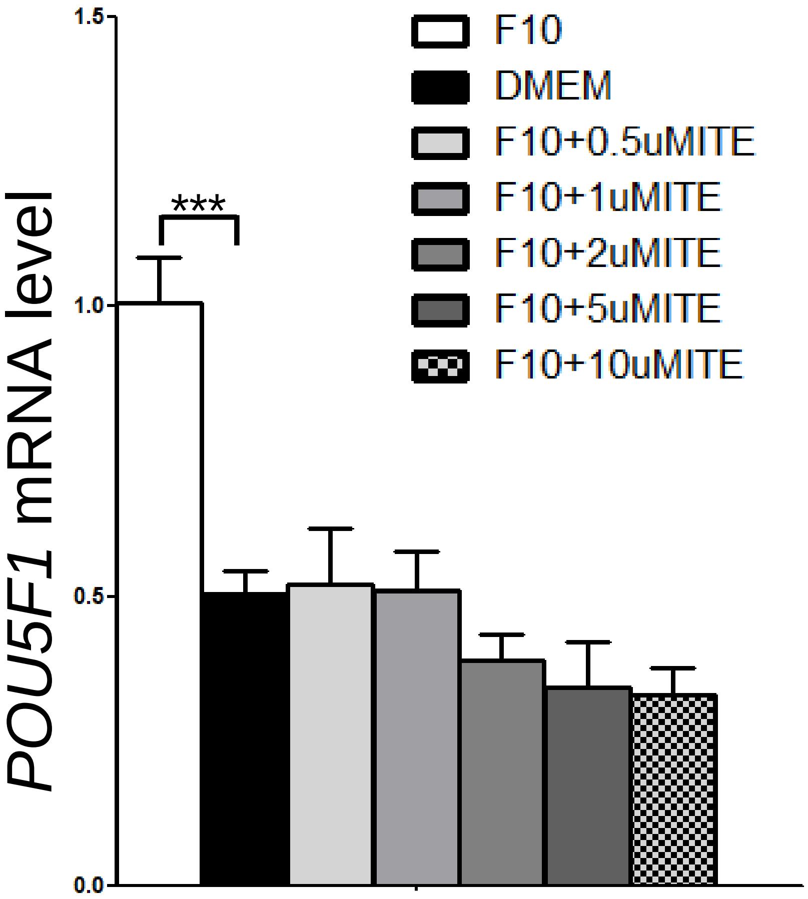

Supplementary Fig. 17. Dose effects of ITE on *POU5F1* mRNA levels in U87 cells.

U87 cells grown in tryptophan-rich medium (DMEM) or low-tryptophan medium (F10) supplemented with increasing concentrations of ITE for 8 hours. Cells were harvested, and analyzed by qRT-PCR for *POU5F1* mRNA levels. The data were expressed as mean ± SD of triplicate measurements from one of three independent experiments. ***P<0.001.

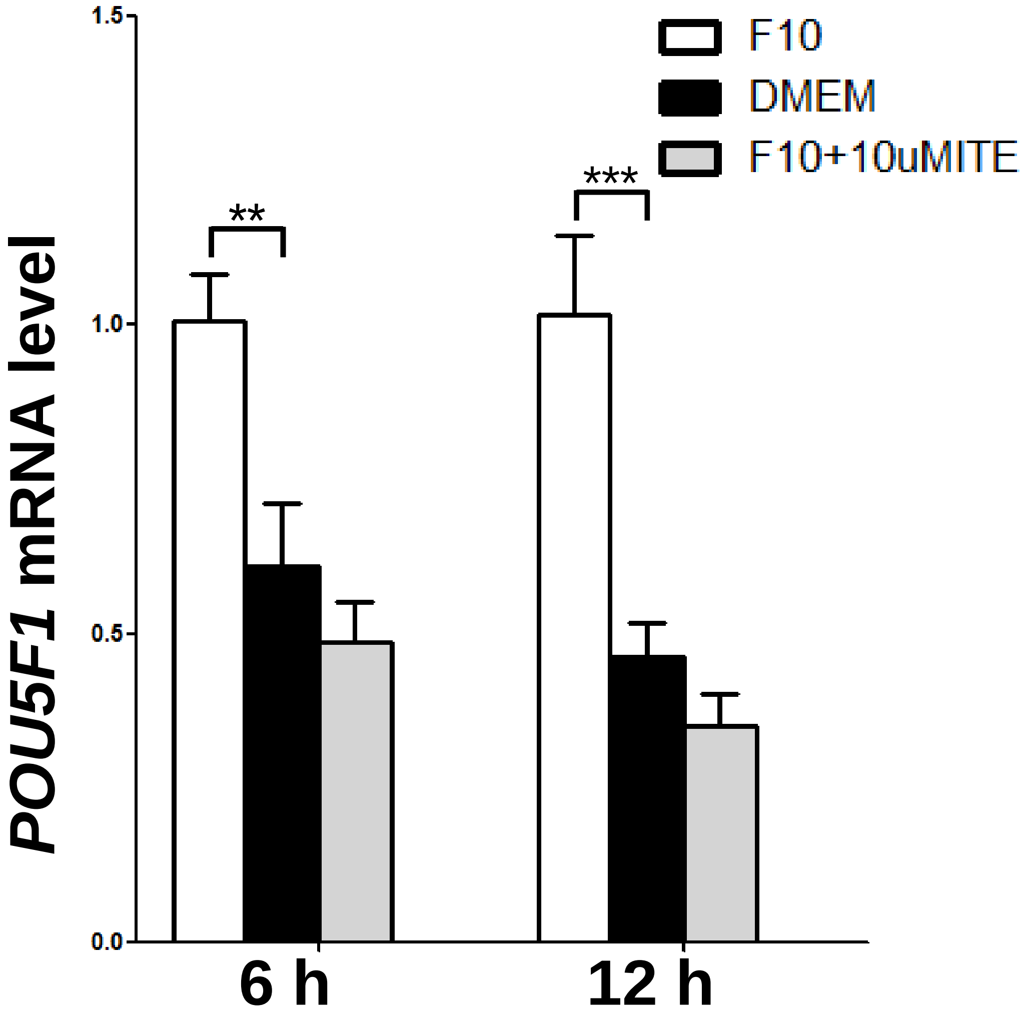

Supplementary Fig. 18. Time course of the effects of ITE on *POU5F1* mRNA levels in U87 cells.

U87 cells grown in tryptophan-rich medium (DMEM) or low-tryptophan medium (F10) supplemented with 10 μM ITE for 6 hours or 12 hours with the compound being replenished every 2 hours. Cells were harvested, and analyzed by qRT-PCR for *POU5F1* mRNA levels. The data were expressed as mean ± SD of triplicate measurements from one of three independent experiments. ***P*<0.01, ****P*<0.001

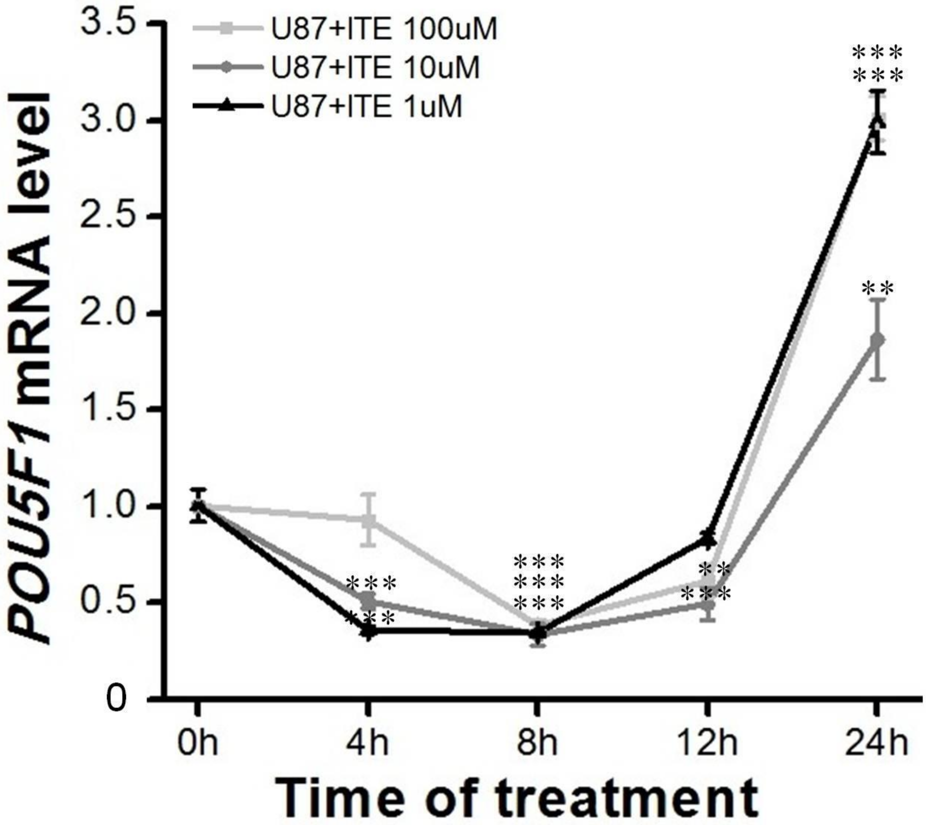

Supplementary Fig. 19. Combined dose- and time-dependent effects of ITE on *POU5F1* mRNA levels in U87 cells.

POU5F1 mRNA abundance in U87 cells grown in DMEM and treated with 1 μM, 10 μM and 100 μM ITE for 0, 4, 8, 12, 24 hours, respectively. *POU5F1* mRNA levels were determined by qRT-PCR and expressed relative to *GAPDH* mRNA. The data were analyzed by ANOVA, and expressed as mean ± SD of triplicate measurements from one of three independent experiments which gave similar results. *$P<0.05$, **$P<0.01$, *** $P<0.001$.

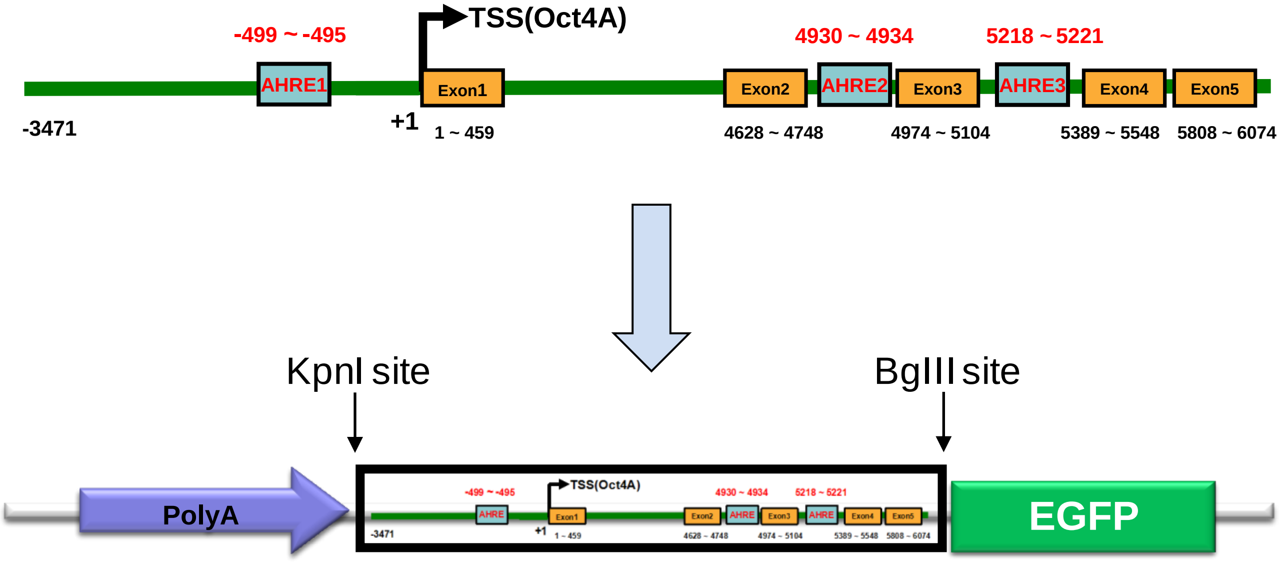

Supplementary Fig. 20. The schematic representation of key elements of phOct4-EGFP reporter plasmid.

A PCR-amplified DNA fragment containing the human *POU5F1* (*OCT4A*) promoter, five exons and all introns in between (with a total length of approximately 13 kb, marked by the black frame) was cloned into the KpnI/BglII restriction sites of a modified vector pGL4.10 in which the original *luc2* is replaced by the *EGFP*, so that the expression of the *EGFP* reporter gene is driven by the *POU5F1* promoter. Three AhR responsive elements (AHREs) are marked with red letters.

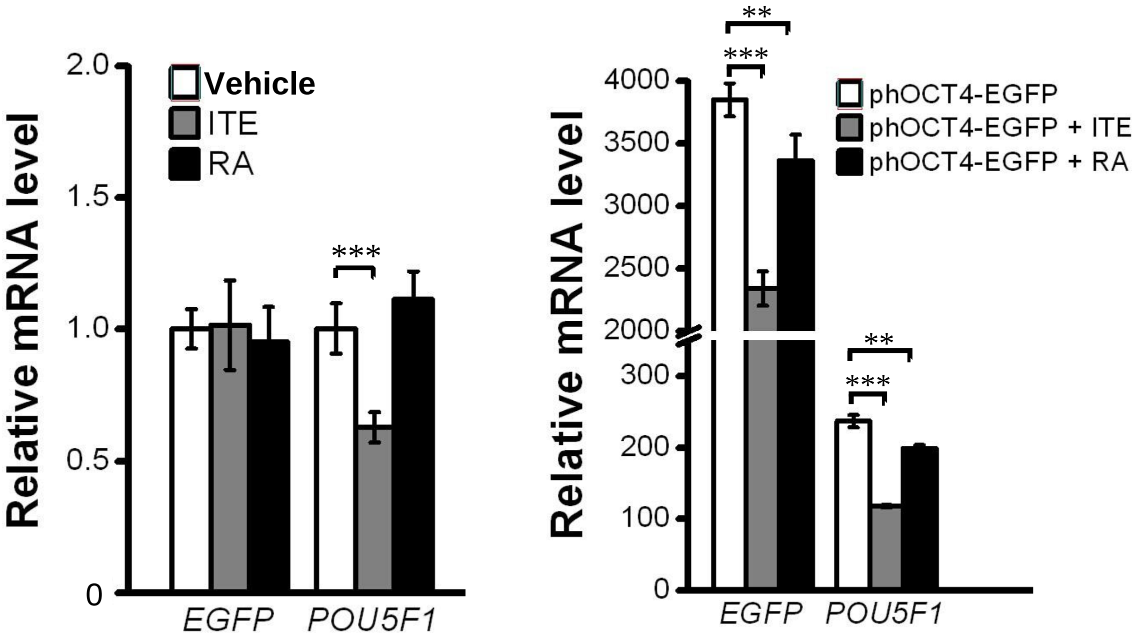

Supplementary Fig. 21. Effects of ITE and RA on endogenous and over-expressed Oct4 mRNA levels.

Left, U87 cells were treated with vehicle (DMSO), 1 μM ITE or 10 μM RA for 8 hours. Cells were harvested, and analyzed by qRT-PCR for mRNA levels of the indicated genes. Right, U87 cells were transfected with phOCT4-EGFP reporter plasmid and treated with ITE (1 μM) or RA (10 μM) for 24 h. Cells were harvested, and analyzed by qRT-PCR for mRNA levels of the indicated genes. The data were expressed as mean ± SD from three independent experiments. **P<0.01, *** P<0.001.

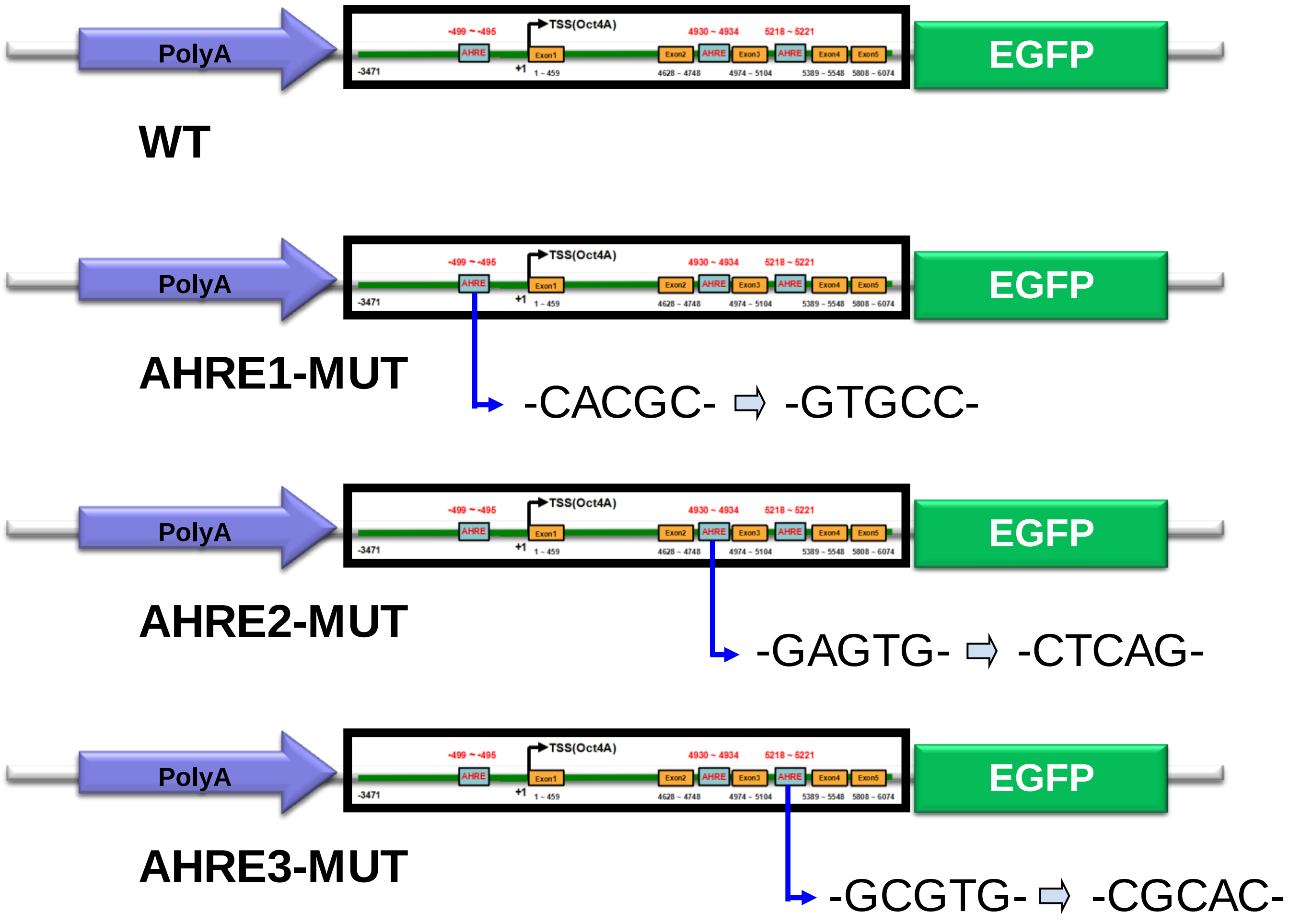

Supplementary Fig. 22. The schematic representation of key elements of phOct4-*EGFP* reporter-based constructs harboring AHRE mutants.

The expression of the *EGFP* reporter gene is driven by the *POU5F1* promoter that harbors either normal AHRE (WT) or one of the AHRE mutants whose positions and nucleic acid sequences were indicated.

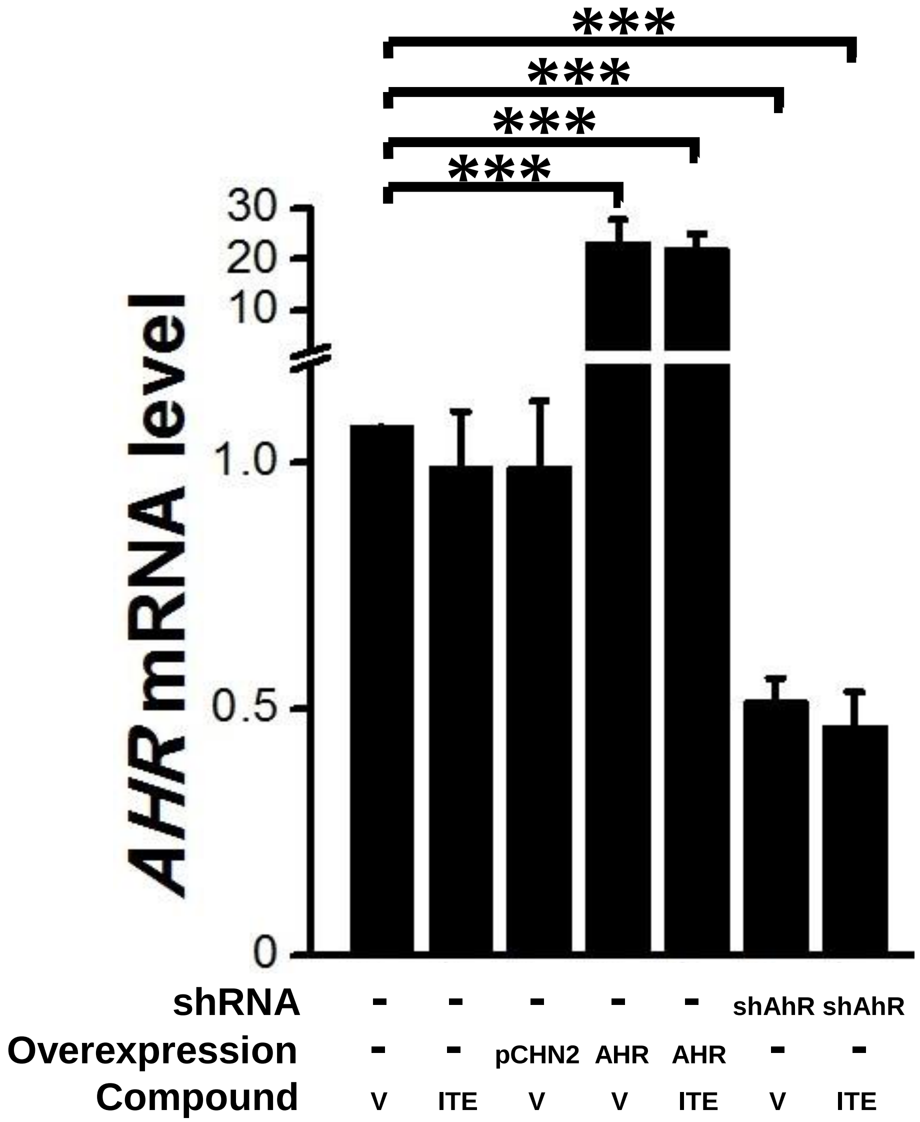

Supplementary Fig. 23. Effects of ITE on *AHR* mRNA levels in U87 cells.

U87 cells were infected with lentiviruses harboring an empty vector pCHN2 (pCHN2), the *Flag-AHR* (AHR) or an shRNA against AHR (shAhR) for 3 days, treated with vehicle (V) or 10 μM ITE (ITE) for 8 h, and analyzed by qRT-PCR for the mRNA levels of *AHR*. The data were expressed as mean ± SD of triplicate measurements from one of three independent experiments. ***P<0.001.

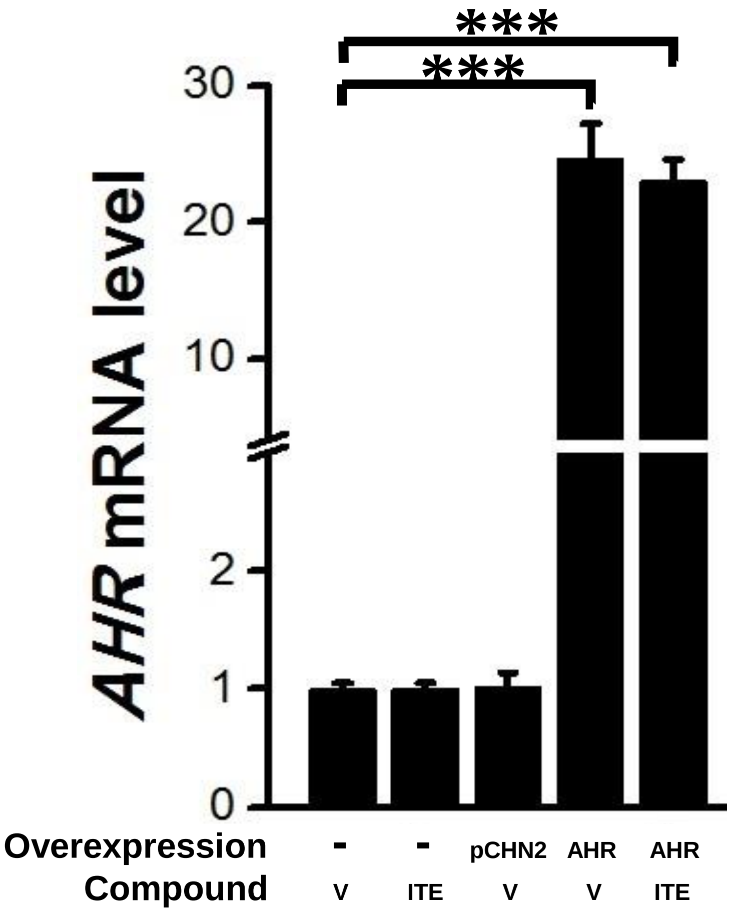

Supplementary Fig. 24. Effects of ITE on *AHR* mRNA levels in NCCIT cells.

NCCIT cells were infected with lentiviruses harboring an empty vector pCHN2 (pCHN2) or the *Flag-AHR* (AHR) for 3 days, treated with vehicle (V) or 10 μM ITE (ITE) for 2 h, and analyzed by qRT-PCR for the mRNA levels of the *AHR*. The data were expressed as mean ± SD of triplicate measurements from one of three independent experiments. ****P*<0.001.

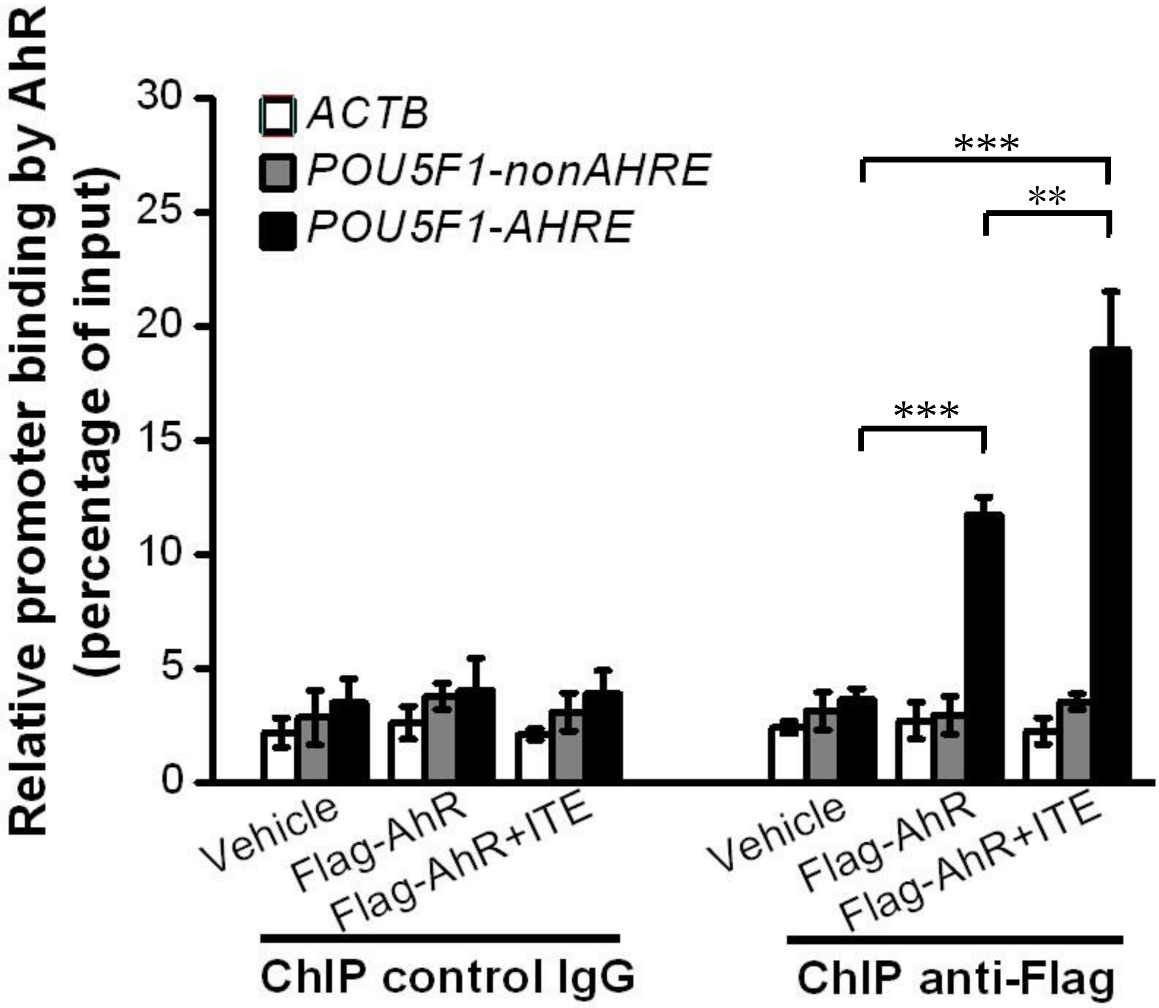

Supplementary Fig. 25. ITE promotes the binding of AhR to the *POU5F1* promoter as determined by ChIP. U87 cells were infected with lentiviruses harboring *Flag-AHR* for 3 days and treated with 10 μM ITE for 2 h. DNA fragments were immunoprecipitated with anti-Flag, amplified with primers described in Supplementary Fig. 9, and quantified by qPCR. The data were expressed as mean ± SD of triplicate measurements from one of three independent experiments. **P<0.01, ***P<0.001.

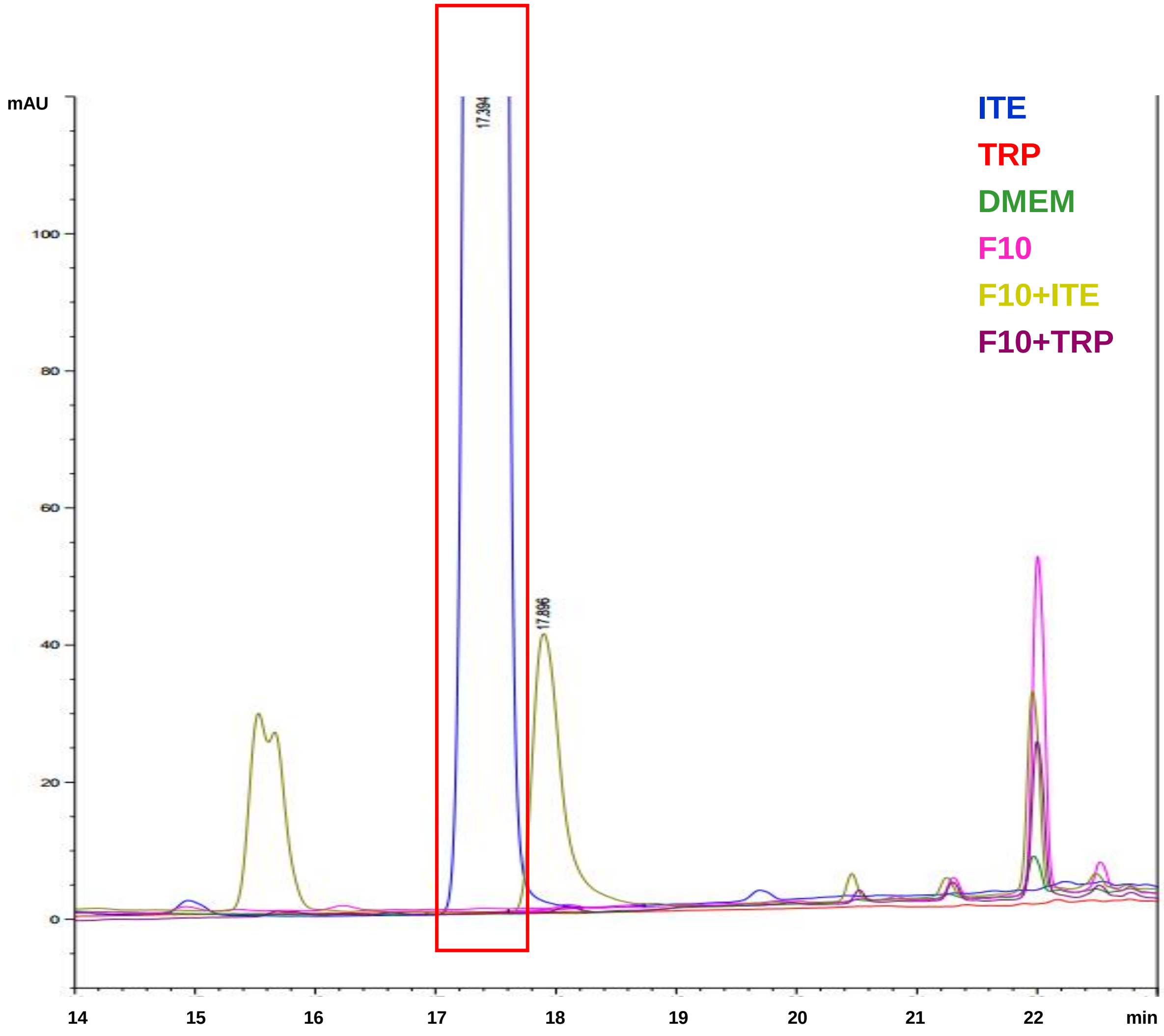

Supplementary Fig. 26. Identification of ITE and its derivatives in hypoxia-exposed U87 cell cultures.

Reverse phase HPLC elution profile of metabolites of U87 cells pre-exposed in 1% O_2 in DMEM for 24 hours followed by culture in 1% O_2 in either DMEM or F10 medium supplemented with vehicle, 10 μM ITE, or 1 μM tryptophan (TRP) for another 8 hours. Purified synthetic ITE (10 μM, blue line) was added as a reference sample. The peak at 17.4 min of retention time presumably corresponds to ITE.

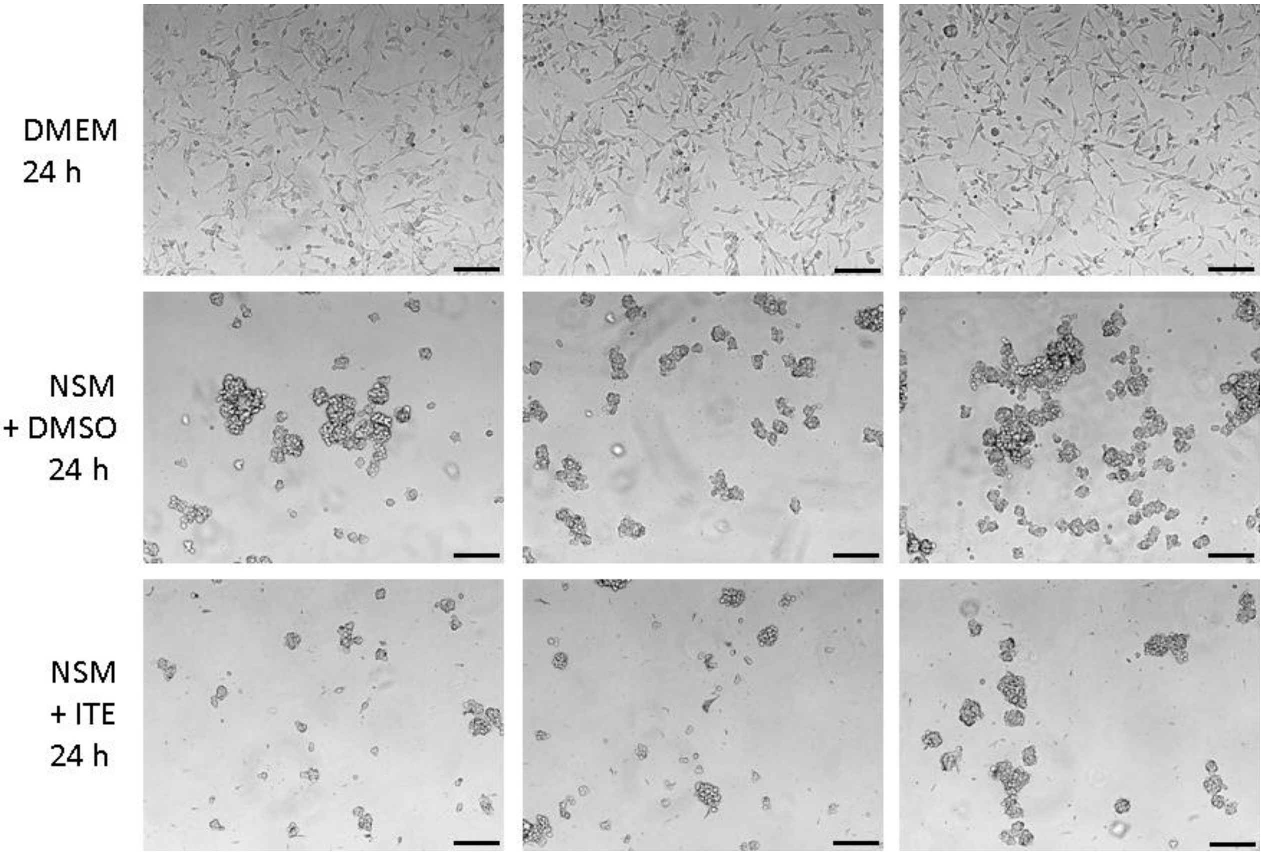

Supplementary Fig. 27. Morphologies of U87 cells grown in neural stem cell medium and treated with ITE.
U87 cells grown in DMEM and neural stem cell medium were treated with vehicle or 10 μM ITE for 24 hours, and the morphologies of those cells were shown. Scale bars, 100 μm.

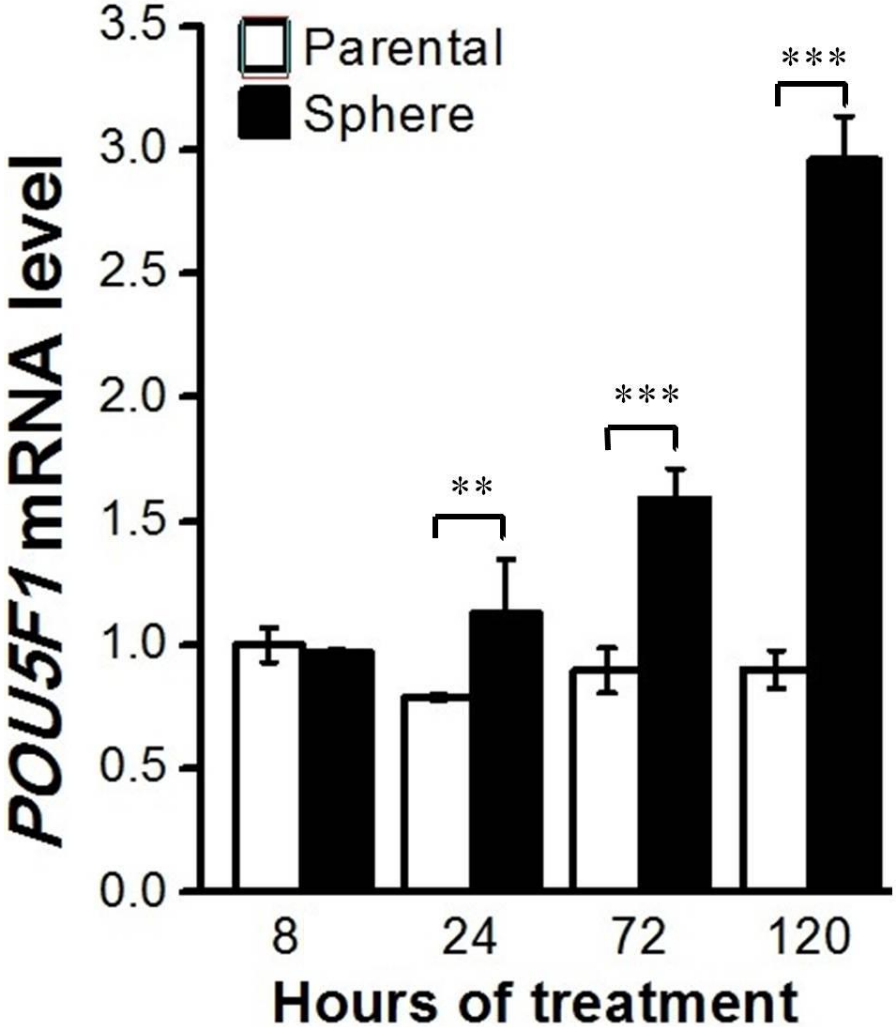

Supplementary Fig. 28. Time course of *POU5F1* mRNA levels during the formation of U87 tumor spheres.
POU5F1 mRNA abundance in parental U87 cells and U87 sphere cells 8, 24, 72, 120 hours after the formation of tumor spheres. *POU5F1* mRNA levels were determined by qRT-PCR and expressed relative to *GAPDH* mRNA. The data were analyzed by ANOVA, and expressed as mean ± SD of triplicate measurements from one of three independent experiments. ***P*<0.01, *** *P*<0.001.

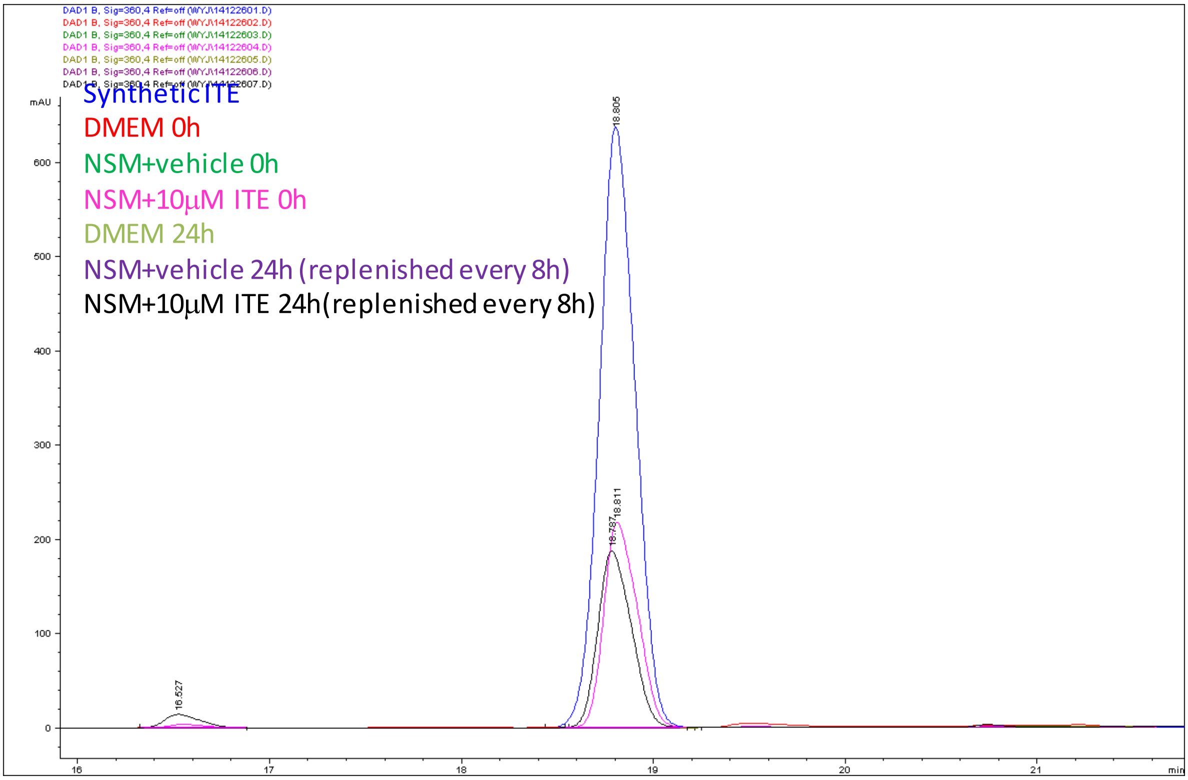

Supplementary Fig. 29. ITE levels in U87 tumor spheres. Reverse phase HPLC elution profile of U87 cells cultured in DMEM or neural stem cell medium (NSM) supplemented with vehicle (DMSO) or 10 μM ITE for 24 hours with the compounds being replenished every 8 hours. Purified synthetic ITE was added as a reference sample. The peak at 18.8 min of retention time presumably corresponds to ITE. The ITE peak area in NSM+ITE 0 h group (pink line) and in NSM+ITE 24 h (replenished every 8 h) group (black line) was approximately 1/3 and 1/4 of that of the synthetic ITE standard (10 μM, blue line). Since the recovery rate during HPLC sample preparation was ~ 40%, the actual ITE concentration in the two groups was therefore approximately 8 μM and 6 μM, respectively, suggesting that replenishing ITE every 8 h was able to effectively maintain its cellular level. Note there was no endogenous ITE peak detected in U87 cells grown in NSM+vehicle for 24 hours (purple line).

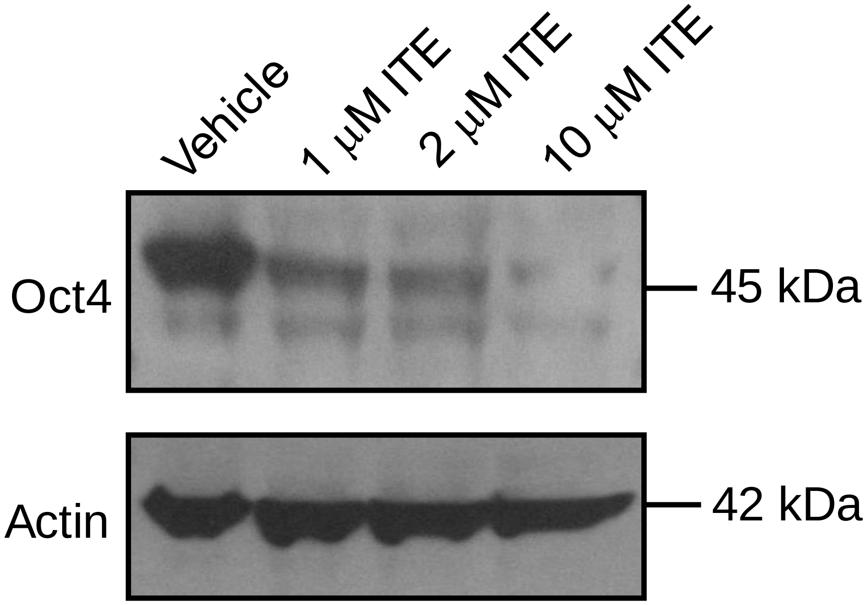

Supplementary Fig. 30. Effects of different concentrations of ITE on Oct4 protein levels in U87 tumor spheres.

Prolonged treatment of U87 sphere cells with varying concentrations of ITE. U87 tumor spheres were formed in NSC medium and 1 μM, 2 μM or 10 μM of ITE was added at an interval of 12 hours for 7 consecutive days. Cells were harvested and immunoblotted with an anti-Oct4 or an anti-Actin.

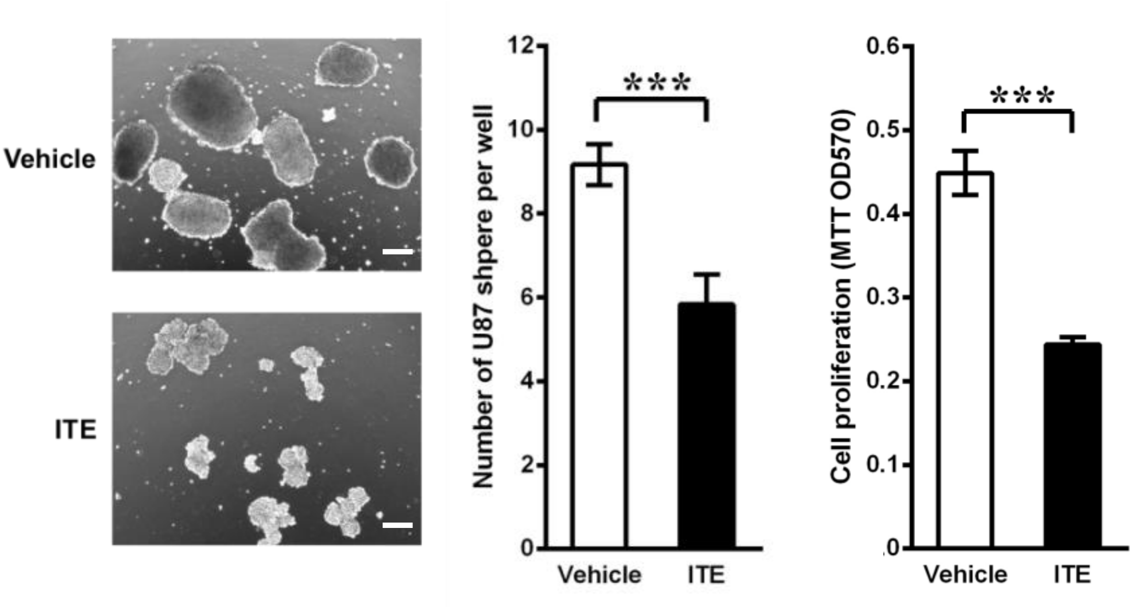

Supplementary Fig. 31. Effects of ITE on the expansion of U87 tumor spheres.

U87 cells grown in a neural stem cell medium were treated with vehicle (DMSO) or 10 μM ITE for 5 days, the numbers of tumor sphere in the two groups were counted, and the viable cell numbers were determined by MTT assay. Scale bars, 200 μm.

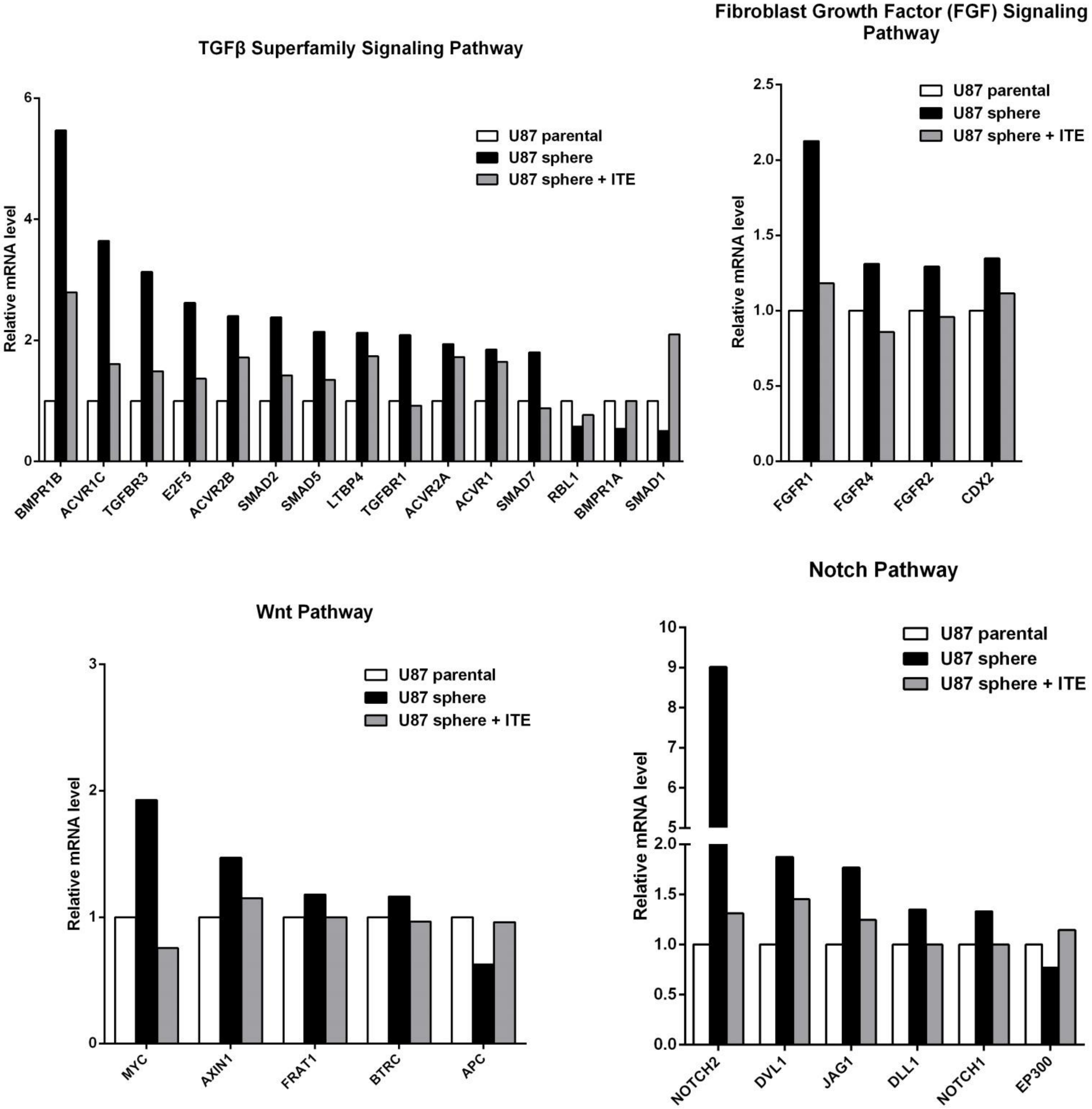

Supplementary Fig. 32. Signaling pathways up-regulated in U87 tumor spheres and suppressed by ITE.

Selected genes whose expression levels were altered significantly in U87 sphere cells over their parentals and rescued by ITE treatment. Four relevant signaling pathways were presented. Full information of these genes can be found in Supplementary Table 3.

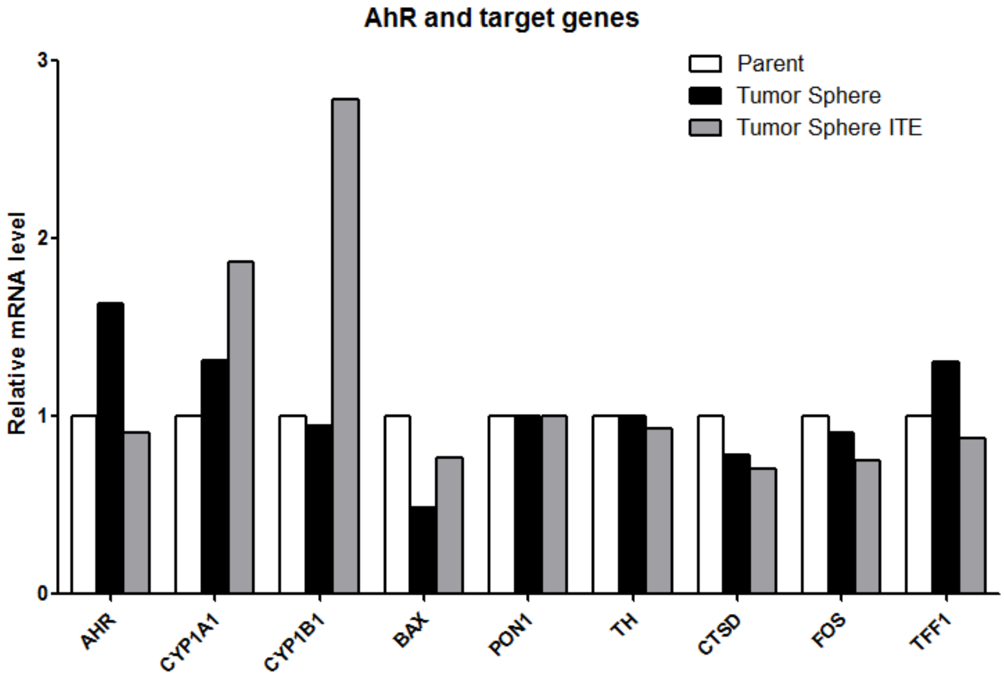

Supplementary Fig. 33. AhR pathway was not significantly affected during the formation of U87 tumor spheres.

The expression levels of key AhR target genes in U87 parentals and in U87 tumor sphere cells treated with or without ITE.

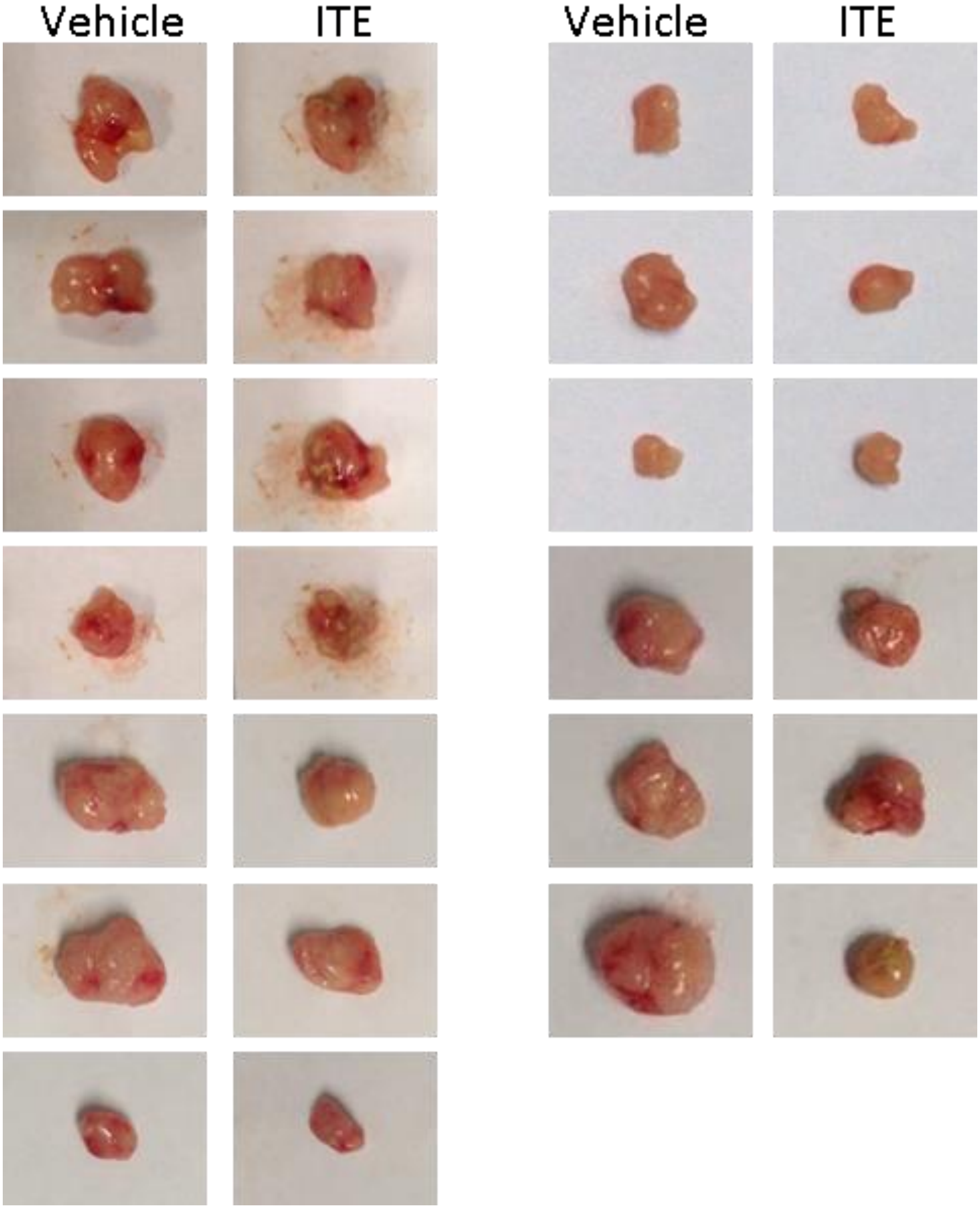

Supplementary Fig. 34. Effects of short-term ITE treatment on xenograft tumor sizes.

U87 cells were inoculated into 26 nude mice. After tumor formation, the mice were divided into 13 pairs based on the apparent tumor volume, and each pair was administered with vehicle (DMSO) or ITE intratumorally for 3 consecutive days. Shown were the tumors excised at day 3.

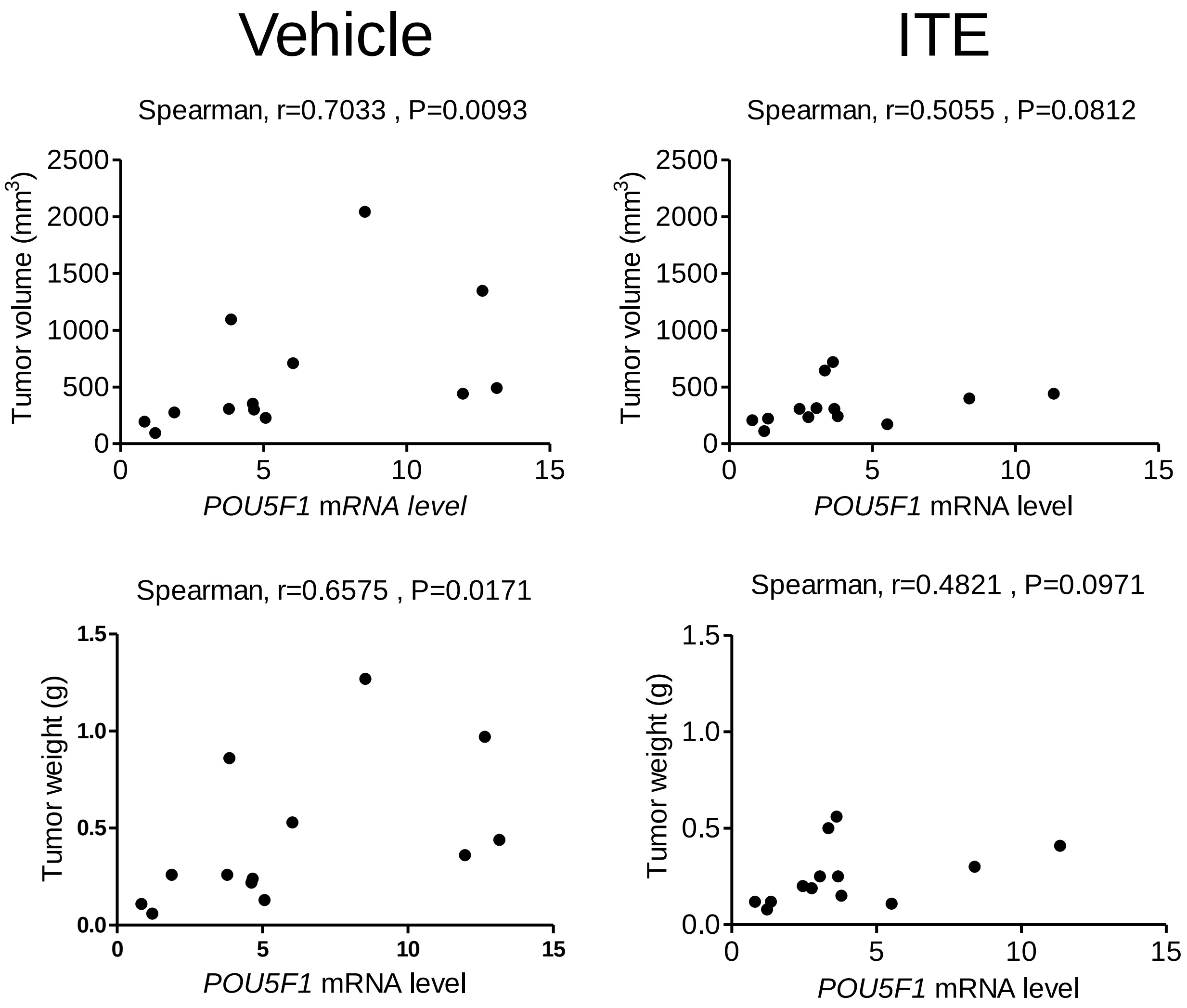

Supplementary Fig. 35. Correlations of *POU5F1* mRNA levels with xenograft tumor volumes and weights.

The excised tumors shown in Supplementary Fig. 34 were measured for tumor volumes and weights, and samples were assayed for *POU5F1* mRNA levels. The correlations between *POU5F1* mRNA level and tumor volume (upper panels), *POU5F1* mRNA level and tumor weight (lower panels) were analyzed with the Spearman's correlation analysis method using SPSS 19.0 statistical software package.

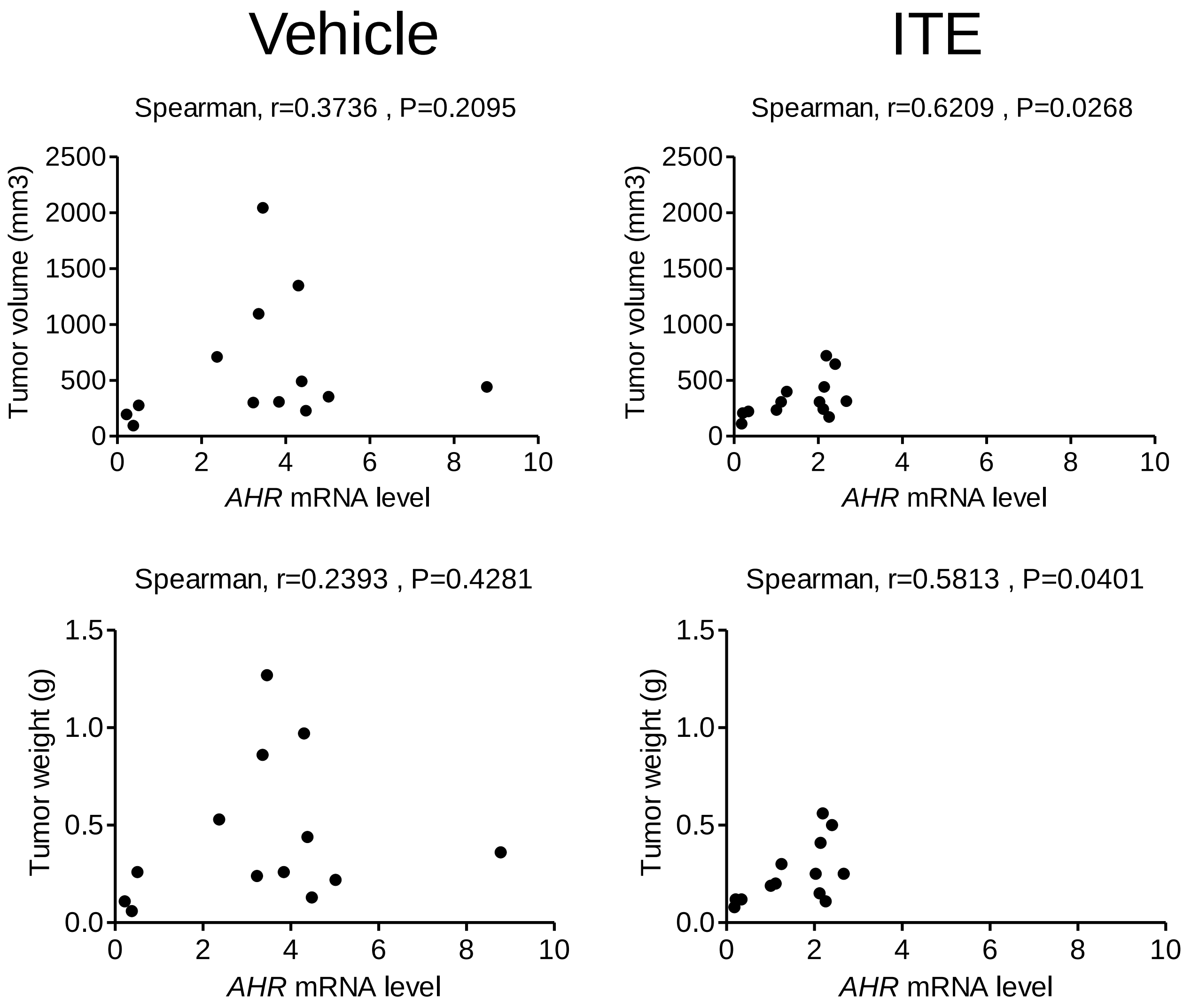

Supplementary Fig. 36. Correlations of *AHR* mRNA levels with xenograft tumor volumes and weights.

The excised tumors shown in Supplementary Fig. 34 were measured for tumor volumes and weights, and samples were assayed for *AHR* mRNA levels. The correlations between *AHR* mRNA level and tumor volume (upper panels), *AHR* mRNA level and tumor weight (lower panels) were analyzed with the Spearman's correlation analysis method using SPSS 19.0 statistical software package.

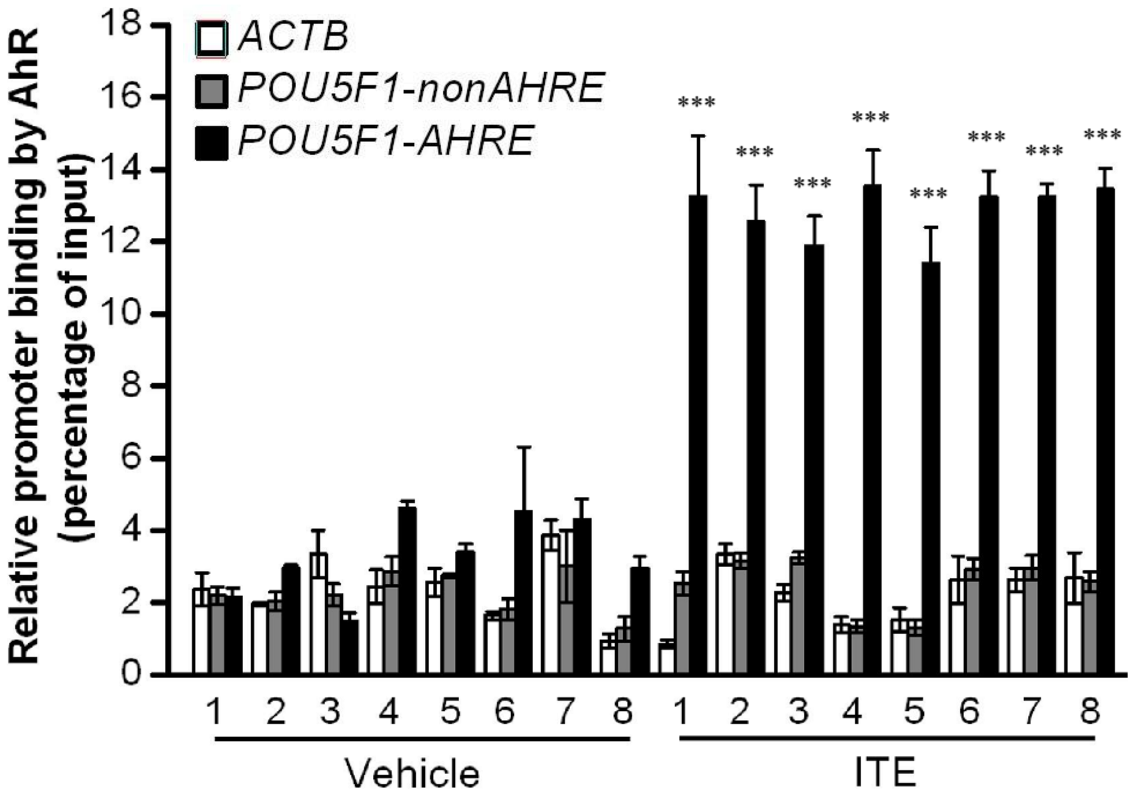

Supplementary Fig. 37. ITE promotes the binding of AhR to the *POU5F1* promoter in xenograft tumors as determined by ChIP.

U87 cells were inoculated subcutaneously into 16 nude mice. After tumor formation, the mice were randomly grouped and intraperitoneally injected with DMSO (vehicle) or ITE at a dose of 80 mg/kg/day for 18 consecutive days. The tumor tissues of the 8 mice in each group as described in Fig 4d were harvested, and the DNA fragments were immunoprecipitated with anti-AhR, amplified with primers described in Supplementary Fig. 9, and quantified by qPCR. The data were expressed as mean ± SD of triplicate measurements from one of three independent experiments. ***P<0.001.

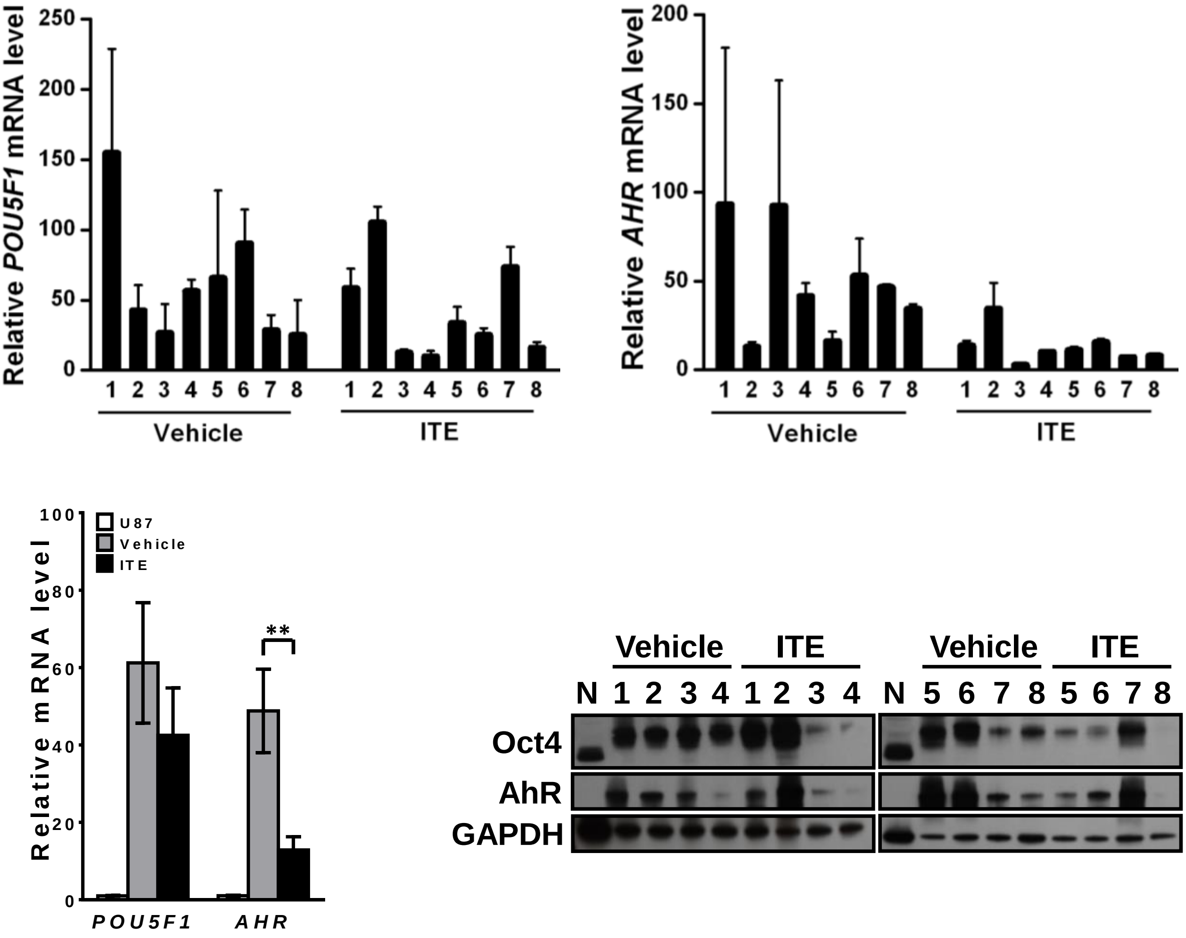

Supplementary Fig. 38. Effects of ITE on Oct4 mRNA and protein levels in U87 xenograft tumors.

The tumor tissues of the 8 mice in each group as described in Fig 4d were harvested, and analyzed by qRT-PCR for the mRNA levels of *POU5F1* (upper left) and *AHR* (upper right). The averaged mRNA levels of *POU5F1* and *AHR* in each group were normalized to those of the parental U87 cells with the latter being set as 1 (lower left). The data were expressed as mean ± SD of triplicate measurements from one of three independent experiments. ***P*<0.01 The tumor samples described in Fig. 4d together with NCCIT cells (N, the first lane) were lysed and analyzed by immunoblotting for indicated proteins (lower right).

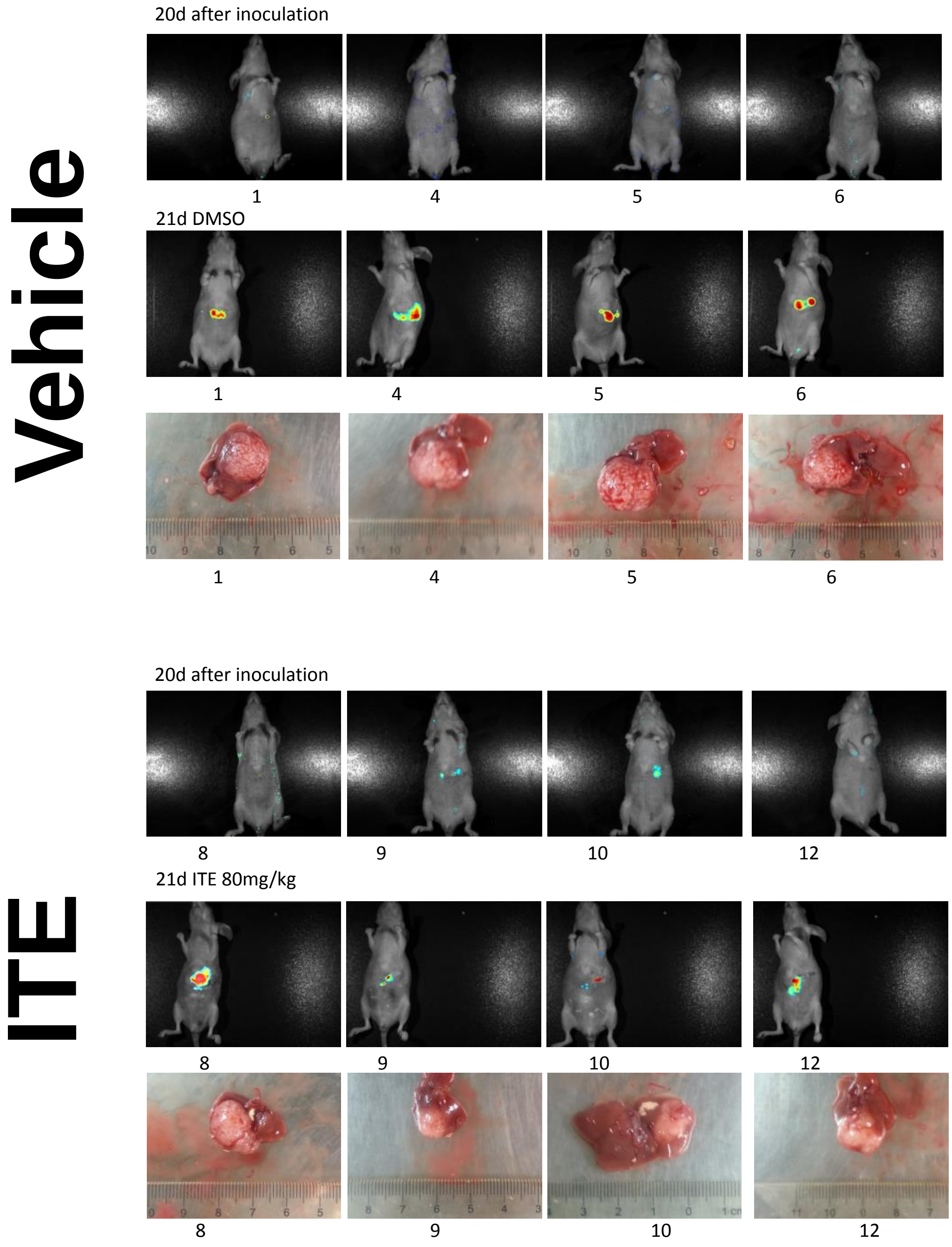

Supplementary Fig. 39. The *in vivo* imaging of mice bearing HCCLM3-RFP cell-derived tumors.

Images were acquired at 20 days after transplantation (top rows) and 21 days after vehicle (DMSO) and ITE treatment (middle rows). The excised tumors were shown in the bottom rows.

	Length(cm)	Width(cm)	Height(cm)	Volume(cm^3)
1	1.58	1.41	1.15	1.34
4	1.56	1.39	1.28	1.45
5	1.66	1.49	1.27	1.64
6	1.53	1.3	1.21	1.26
8	1.64	1.46	0.92	1.15
9	1.03	0.84	0.54	0.24
10	1.30	0.88	0.93	0.56
12	1.34	1.21	0.90	0.76

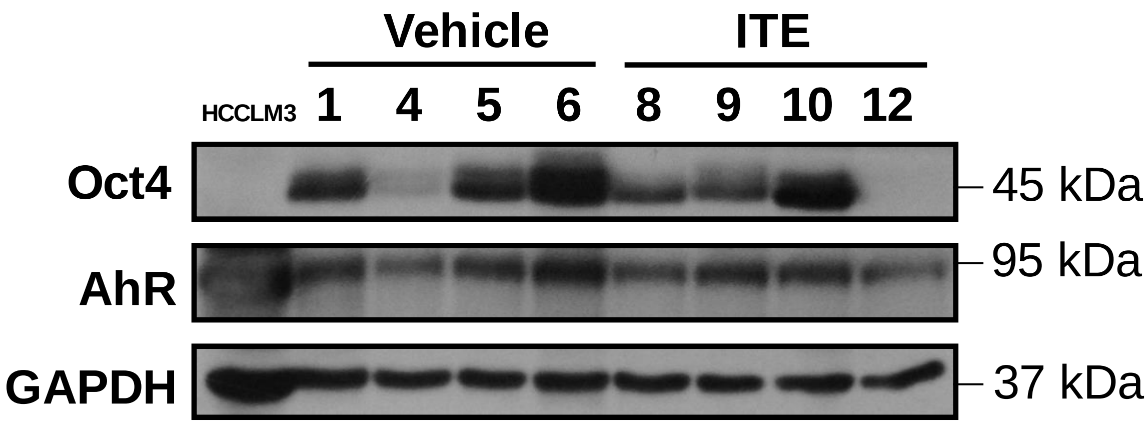

Supplementary Fig. 40. Characterization of HCCLM3-RFP cell-derived tumors.

The volumes of tumors described in Supplementary Fig. 39 were shown in the table (upper). The tumor samples together with the parental HCCLM3-RFP cells (the first lane) were lysed and analyzed by immunoblotting for indicated proteins (lower).

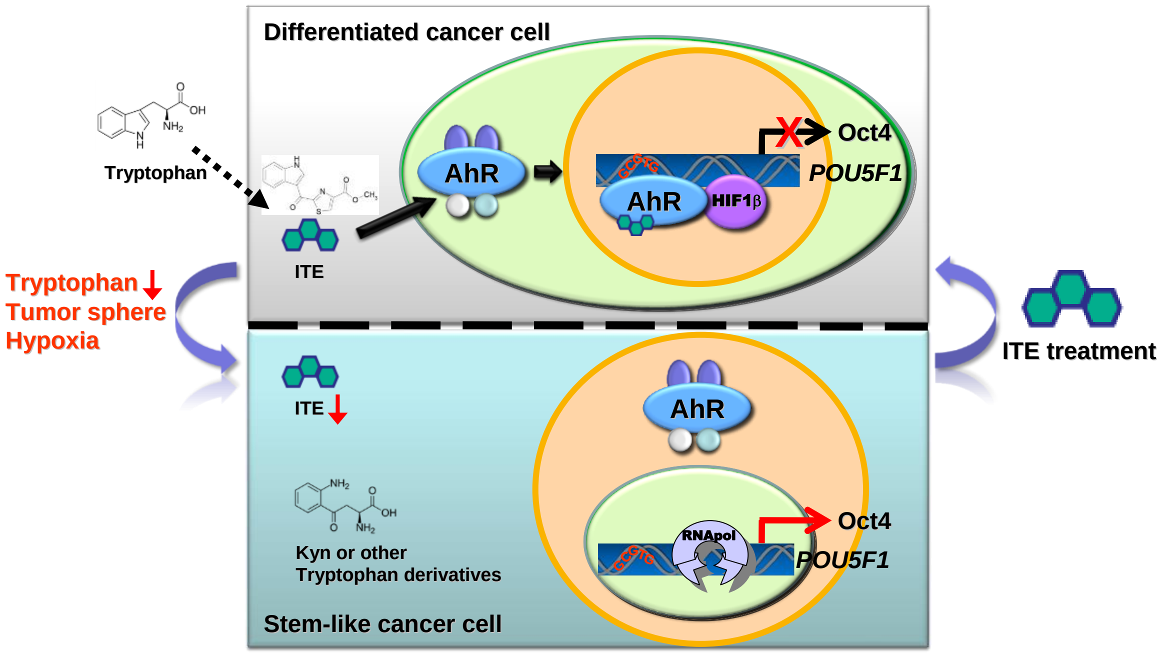

Supplementary Fig. 41. Schematic summary of key findings.

In differentiated cancer cells, endogenous ITE derived from tryptophan activates AhR and strengthens its binding to the AHRE motif (GCGTG, in red) at the *POU5F1* promoter. As a result, the transcription of Oct4 is suppressed. Reduction of endogenous ITE levels by tryptophan deprivation, hypoxia or the formation of tumor sphere cells can relief the suppression and lead to Oct4 expression. Administration of synthetic ITE *in vitro* and *in vivo* results in restored AhR-*POU5F1* promoter binding, the differentiation of tumor sphere cells and their reduced tumorigenic potential in xenograft tumor models.

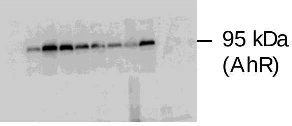

Fig. 1b

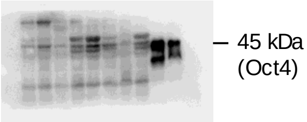

Fig. 1b

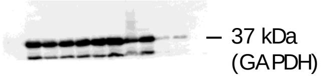

Fig. 1b

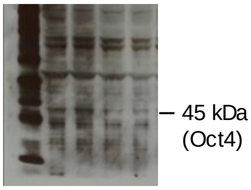

Fig. 2f

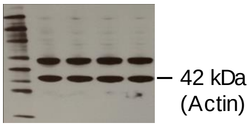

Fig. 2f

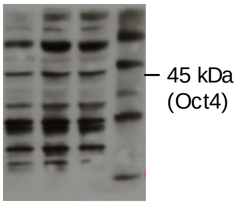

Fig. 3c

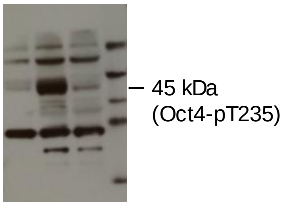

Fig. 3c

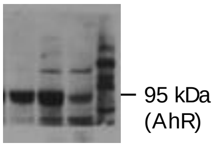

Fig. 3c

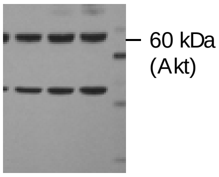

Fig. 3c

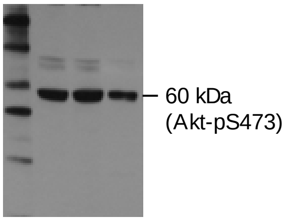

Fig. 3c

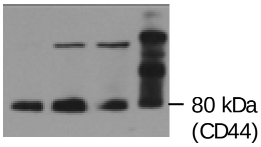

Fig. 3c

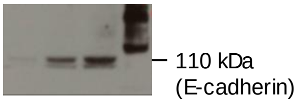

Fig. 3c

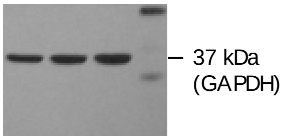

Fig. 3c

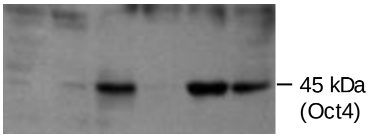

Fig. 3e

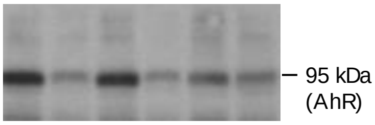

Fig. 3e

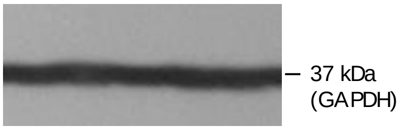

Fig. 3e

Supplementary Fig. 42. Uncropped immunoblots for main figures.

SUPPLEMENTARY TABLES

Supplementary Table 1: Primers for ChIP/qRT-PCR

Target gene (human)	Forward	Reverse
POU5F1-AHRE	GT*TGGGAGT*TGAAAGTTGGG	ATGGGGCCTTGCTATGTTAC
POU5F1-nonAHRE	GGGTCTCTCACATCTCCTAGGC	GTGGGAAAAGGGGCTCAAAC
ACTB	GCATAGCAGACATACAACGGAC	GCACCTTTTACCCTGGAGC

Supplementary Table 2: Primers for qRT-PCR

Target gene (human)	Forward	Reverse
AHR	ATTGTGCCGAGTCCCATATC	AAGCAGGCGTGCATTAGACT
POU5F1	GTGGAGGAAGCTGACAACAA	ATTCTCCAGGTTGCCTCTCA
CYP1A1	CTTGGACCTCTTTGGAGCT	GACCTGCCAATCACTGTG
GAPDH	GGGGAGCCAAAAGGGTCATCATCT	GAGGGGCCATCCACAGTCTTCT
NANOG	CCTGTGATTTGTGGGCCTG	GACAGTCTCCGTGTGAGGCAT
SOX2	GTATCAGGAGTTGTCAAGGCAGAG	TCCTAGTCTTAAAGAGGCAGCAAAC
EGFP	CACAAGTTCAGCGTGTCCG	AGTTCACCTTGATGCCGTTC
